# Sex and Diet Biased Effect of L‐DOPA on Iron Accumulation in the Ventral Midbrain

**DOI:** 10.1111/jnc.70389

**Published:** 2026-03-05

**Authors:** Rebecka O. Serpa, Emily Tufano, Kondaiah Palsa, Timothy B. Helmuth, Sara Mills‐Huffnagle, Mathias Kant, James R. Connor

**Affiliations:** ^1^ Department of Neurosurgery The Pennsylvania State University College of Medicine Hershey Pennsylvania USA; ^2^ Department of Neuroscience and Experimental Therapeutics Sciences The Pennsylvania State University College of Medicine Hershey Pennsylvania USA; ^3^ Penn State Neuroscience Institute The Pennsylvania State University College of Medicine Hershey Pennsylvania USA

**Keywords:** iron accumulation, iron deficiency, iron repletion, L‐DOPA, Parkinson's disease, ventral midbrain

## Abstract

Parkinson's disease (PD) is a progressive neurodegenerative disorder characterized by the loss of dopaminergic neurons in the substantia nigra, a region within the ventral midbrain known to accumulate iron. While L‐3,4‐dihydroxyphenylalanine (L‐DOPA) remains the gold standard treatment for PD, its impact on brain iron homeostasis, particularly under varying systemic iron conditions, remains poorly understood. In this study, we investigate how dietary iron status and anti‐PD treatments influence brain iron accumulation and regulation in the ventral midbrain, with a focus on sex‐specific differences. Male and female Long‐Evans rats were placed on iron‐adequate (IA), iron‐deficient (ID), or iron‐repletion (IR) diets from postnatal day (PND) 21 for eight weeks. In the final three weeks, animals received daily subcutaneous injections of L‐DOPA, selegiline, or vehicle. Our findings revealed that L‐DOPA treatment in IR males significantly increased brain iron levels in the ventral midbrain, whereas females showed no such effect. This sex‐specific accumulation was accompanied by the upregulation of iron uptake protein transferrin receptor 1 (TfR1), increased ferroportin (FPN1), and reduced expression of the iron storage protein ferritin heavy chain (FTH1), indicating disrupted iron homeostasis. Furthermore, L‐DOPA‐treated males on the IR diet exhibited elevated glial fibrillary acidic protein (GFAP) and lipocalin‐2 (LCN2), suggesting enhanced oxidative stress and astrocyte activation. Consistent with this, antioxidant enzymes catalase (CAT) and superoxide dismutase 2 (SOD2) were significantly decreased in L‐DOPA‐treated males on the IR diet, highlighting increased vulnerability to oxidative damage. In contrast, selegiline did not significantly alter brain iron levels or iron‐regulatory protein expression, regardless of diet or sex. These findings demonstrate that systemic iron repletion after deficiency sensitizes the male brain to L‐DOPA‐induced iron accumulation, potentially increasing susceptibility to neurodegeneration. This study highlights the importance of considering that both dietary iron status and biological sex may impact PD treatment strategies.

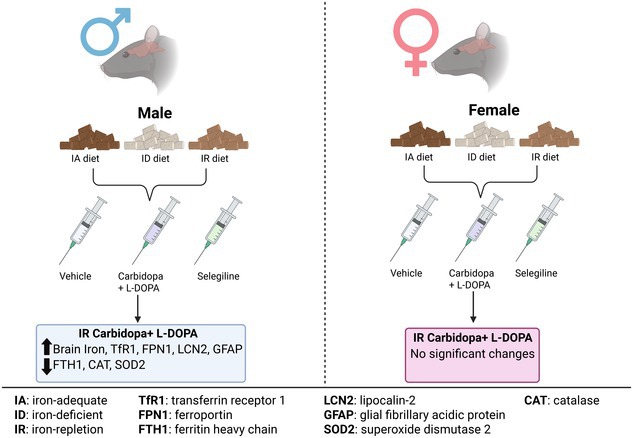

## Graphical abstract

Dietary iron status and antiparkinson treatments influence regulation in the ventral midbrain. Male and female Long‐Evans rats were placed on iron‐adequate (IA), iron‐deficient (ID), or iron‐repletion (IR) diets and treated with L‐DOPA, selegiline, or vehicle. Brain iron levels were significantly increased in only IR males treated with L‐DOPA, whereas females showed no such effect under any dietary condition. This sex‐specific accumulation was accompanied by the upregulation of iron uptake protein transferrin receptor 1 (TfR1), increased ferroportin (FPN1), reduced expression of the iron storage protein ferritin heavy chain (FTH1), and elevated glial fibrillary acidic protein (GFAP) and lipocalin‐2 (LCN2), alongside decreased antioxidant enzymes catalase (CAT) and superoxide dismutase 2 (SOD2), indicating disrupted iron homeostasis and heightened oxidative stress. In contrast, selegiline did not significantly alter brain iron levels. These findings demonstrate that iron repletion after deficiency sensitizes the male brain to L‐DOPA‐induced iron accumulation, potentially increasing susceptibility to neurodegeneration. This study highlights the importance of considering that both dietary iron status and biological sex may impact PD treatment strategies.

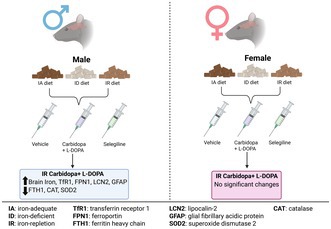

**Abbreviations**
BBB: blood–brain barrier
CAT: catalase
FPN1: ferroportin
FTH1: ferritin heavy chain
FTL: ferritin light chain
GFAP: glial fibrillary acidic protein
IA: iron‐adequate
ICP‐AES: inductively coupled plasma atomic emission spectroscopy
ID: iron‐deficient
IR: iron‐repletion
LCN2: lipocalin‐2
LCN‐R: lipocalin‐receptor
L‐DOPA: L‐3,4‐dihydroxyphenylalanine
MAO‐B: monoamine oxidase‐B
PD: Parkinson's disease
PND: postnatal day
RRID: Research Resource Identifier, see scicrunch.org
SEM: standard error of the mean
SOD2: superoxide dismutase 2
TBST: tris‐buffered saline with Tween
TfR1: transferrin receptor 1

## 1 Introduction

Iron plays a critical role in brain development and function, influencing processes such as neurotransmitter synthesis, neuronal development, and cellular function (Beard and Connor 2003; Levi et al. 2024; Piñero et al. 2000; Teh et al. 2024). Iron dysregulation, whether due to accumulation or deficiency, plays a critical role in Parkinson's disease (PD) pathology initiation and progression (Deng et al. 2017; Hong et al. 2016). Iron accumulation in the substantia nigra during PD diminishes dopamine function, contributing to the loss of motor function, cognition, and reward processing, characteristics of the disease (López‐Aguirre et al. 2025; Lozoff 2011). Additionally, the loss of dopamine in the ventral midbrain has been linked to the degeneration of the nigrostriatal pathway, particularly in the substantia nigra, further contributing to PD symptom (Lozoff 2011; Luo and Huang 2016).

L‐3,4‐dihydroxyphenylalanine (L‐DOPA), a dopamine precursor, remains the gold standard treatment for PD and replenishes striatal dopamine, improving motor symptoms (Billings et al. 2019). However, chronic L‐DOPA use has been associated with increased oxidative stress and iron accumulation, particularly in the ventral midbrain (Du et al. 2022; Zeng et al. 2024), ultimately promoting neurodegeneration (Hörmann et al. 2021; Stansley and Yamamoto 2013). To mitigate these side effects, monoamine oxidase‐B (MAO‐B) inhibitors, such as selegiline, are often prescribed by physicians as an initial monotherapy treatment to delay L‐DOPA initiation (Goldenberg 2008), reduce dopamine breakdown, and promote neuroprotection through potential antioxidant and anti‐inflammatory properties (Cho et al. 2021; Mizuno et al. 2019). Remarkably, a recent clinical study revealed that drug‐naïve PD patients had lower nigral iron levels compared to healthy controls, while those treated with L‐DOPA exhibited higher iron accumulation. Selegiline, in contrast, appeared to reduce iron accumulation in the ventral midbrain (Du et al. 2022).

While observational studies have linked iron supplementation to possible PD risk, the underlying mechanisms, particularly in the context of dopaminergic therapy, remain poorly understood (Takeuchi and Kawashima 2022; Zeidan et al. 2024). Many PD patients may also concurrently suffer from iron deficiency anemia, another risk factor for PD, and may be recommended or prescribed iron supplementation, creating a complex therapeutic scenario in which iron repletion occurs alongside L‐DOPA treatment (Hong et al. 2016; Kim et al. 2021; López‐Aguirre et al. 2025; Savica et al. 2009; Wang et al. 2021). However, the consequences of this interaction remain largely unexplored, even in preclinical models. Furthermore, emerging evidence also indicates that sex modulates iron metabolism and dopaminergic signaling, which may contribute to the observed differences in PD risk and treatment outcomes between males and females (Cerri et al. 2019; Dorsey et al. 2018; Rozani et al. 2019). Taken together, in the context of ongoing L‐DOPA treatment, the consequences of iron repletion, alongside the lesser‐understood sex‐specific differences in iron metabolism, there remains a significant and critical gap in patient care outcomes.

This current study aims to address these knowledge gaps by examining the combined effects of dietary iron status (iron adequacy (IA), iron deficiency (ID), and iron repletion (IR)), and anti‐PD treatments (L‐DOPA and selegiline) on brain iron regulation in the ventral midbrain. This study offers a model to further investigate prior clinical findings to determine how iron repletion administered concurrently with L‐DOPA influences brain iron accumulation, iron‐related protein expression, and oxidative stress. We hypothesized that both iron status and sex modulate brain iron homeostasis during L‐DOPA treatment. Specifically, we predicted that iron repletion in combination with L‐DOPA would exacerbate iron accumulation in the ventral midbrain. To test this, we measured brain iron concentration and evaluated expression levels of key iron‐related and neuroinflammatory markers including transferrin receptor 1 (TfR1), ferroportin (FPN1), ferritin heavy chain (FTH1), ferritin light chain (FTL), lipocalin‐2 (LCN2), its receptor (LCN‐R), glial fibrillary acidic protein (GFAP), catalase (CAT), and superoxide dismutase 2 (SOD2). By integrating measures of iron transport, storage, and oxidative stress, this study uncovers sex‐specific regulatory mechanisms governing brain iron metabolism during PD treatment. Our findings offer novel insights into how dietary iron and anti‐PD drugs interact to influence PD pathogenesis and may inform future optimized therapeutic strategies to address PD and iron deficiency.

## 2 Methods

All experimental protocols and procedures were conducted with approval by the Penn State University Institutional Animal Care and Use Committee (#PROTO202302586) and in compliance with the National Institutes of Health eighth edition *Guide for the Care and Use of Laboratory Animals*.

### 2.1 Animals

A total of 108 Long‐Evans (strain HsdBlu:LE) male (*n* = 54) and female (*n* = 54) rats were obtained from Inotiv Laboratory (Denver, PA, cat. no. 140) starting at postnatal day (PND) 21. The sample size was determined using G*Power 3.1 with *F* test; ANOVA; fixed effects, special, main effects, and interactions (significance level *α* = 0.05, power = 0.80), which indicated *n* = 6 rats per sex per diet per drug group (total *n* = 108; Faul et al. 2009). Before the start of the experiment, all animals were given one week to acclimate to their environment and were maintained in a standard temperature environment of 24°C ± 1°C and a 12‐h light/dark cycle with lights on at 07:00 h and off at 19:00 h. Animals were housed in pairs in standard polycarbonate ventilated cages with ad libitum access to food and water. If an animal exceeded 500 g during the study, it was singly housed to maintain adequate space and comfort. Experimenters were blinded to conditions.

### 2.2 Diets & Experimental Procedure

**FIGURE 1:**
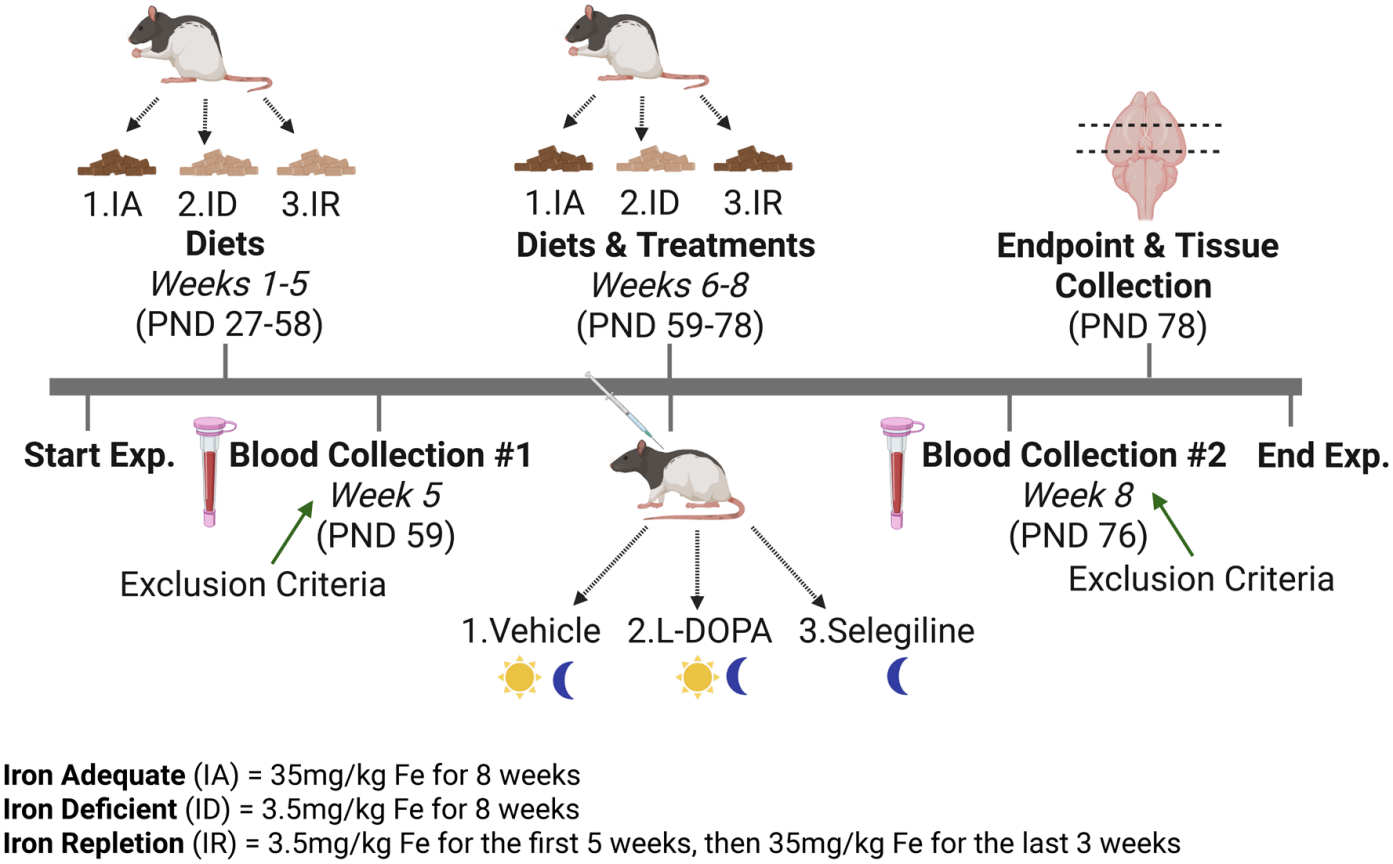
Experimental timeline for dietary iron and drug treatment. Starting on postnatal day (PND) 20, rats were assigned one of three diet groups: 1. iron‐adequate (IA) controls (35 mg/kg Fe, AIN‐93G, *n* = 36), 2. iron‐deficient (ID) diet (3.5 mg/kg Fe, #AIN‐93, *n* = 36), or iron repletion (IR) diet (3.5 mg/kg Fe, #AIN‐93 for 5 weeks followed by 35 mg/kg diet, AIN‐93G for 3 weeks, *n* = 36). After 5 weeks (PND59), hemoglobin and hematocrit were measured to confirm iron status. Pre‐determined exclusion criteria required that animals assigned to the ID or IR group exhibit hematological evidence of iron deficiency by PND59 and repletion by PND76. Animals failing to meet this threshold would have been excluded. No animals were excluded. Animals were then randomized to begin 20 days of drug treatment (PND59‐78): 1. vehicle (*n* = 6 rats per group), 2. carbidopa+L‐DOPA (*n* = 6 rats per group), or 3. selegiline (*n* = 6 rats per group). Vehicle and carbidopa+L‐DOPA were administered every 12 h (carbidopa was given 30 min before L‐DOPA), while selegiline was administered every 24 h during the dark cycle. Animals remained on their assigned diets throughout the treatment, except for the IR group, which switched to the IA diet once iron deficiency was confirmed.

Dietary iron deficiency and repletion protocols followed established methods (Bianco et al. 2008). On PND27, animals were assigned to diet groups and treatments (Table 1) using a simple randomization procedure. Upon arrival, each rat was marked with a nontoxic tail ID code and assigned a unique number (#1‐#108) in the order in which they were weighed on PND27. A randomization list was then generated on Microsoft Excel. Animals were sorted according to their randomly generated numbers, resulting in three diet groups of equal size (*n* = 36): 1. iron‐adequate (IA, *n* = 36; 35 mg/kg Fe, AIN‐93G, Inotiv, cat. no. TD.94045), 2. iron‐deficient (ID, *n* = 36; 3.5 mg/kg Fe, #AIN‐93, Inotiv, cat. no. TD.10210), or 3. iron repletion (IR, *n* = 36; 3.5 mg/kg Fe, #AIN‐93 for 5 weeks followed by 35 mg/kg diet, AIN‐93G for 3 weeks). After diet assignment, cage placements were made, and animals were pair‐housed with a randomly assigned drug‐treatment partner of the same sex and same diet. This approach ensured unbiased allocation and equal group sizes across all conditions. At the start of the experiment, the average body weight was 73.87 ± 1.54 g for males and 66.35 ± 1.21 g for females. All diets were formulated based on the guidelines set by the American Institute of Nutrition as described by Reeves et al. (1993). Nutritional requirements were met except for the iron content in the iron‐deficient diet. Standard formulations of chow can vary from 130 to 400 mg/kg of iron, therefore, to remove variation and to reflect a realistic human consumption of iron, the iron‐adequate diet was selected as the control diet. At five (PND59) and eight (PND76) weeks, blood samples were collected to evaluate hemoglobin and hematocrit levels to confirm ID or IA. Pre‐determined exclusion criteria required that animals assigned to the ID or IR group exhibit hematological evidence of iron deficiency by PND59 and repletion by PND76. Animals failing to meet this threshold would have been excluded. No animals met the exclusion criteria, no animals died during the experiment, and no replacements were made. All animals were also randomized to start drug treatments on PND59 for 20 days (Figure 1). Animals on the IA or ID diet continued the diet for 20 days, concurrent with treatment regimens as described below. Within the IR group, once deficiency was confirmed on PND59, animals began the IA diet to represent an IR group for 20 days. At the start of treatments, the average body weight was 279.89 ± 3.86 g for males and 195.58 ± 2.06 g for females.

**TABLE 1:** Experimental design: Dietary conditions, drug treatments, doses, & group sizes.

Treatments	Treatment dose	Treatment timing	Diets	Males	Females
Vehicle	0 mg/kg	Every 12 h	Iron Adequate (IA)	*N* = 6	*N* = 6
			Iron Deficient (ID)	*N* = 6	*N* = 6
			Iron Repletion (IR)	*N* = 6	*N* = 6
Carbidopa+L‐DOPA	5 + 20 mg/kg	Every 12 h	IA	*N* = 6	*N* = 6
			ID	*N* = 6	*N* = 6
			IR	*N* = 6	*N* = 6
Selegiline	0.3 mg/kg	Every 24 h	IA	*N* = 6	*N* = 6
			ID	*N* = 6	*N* = 6
			IR	*N* = 6	*N* = 6
			Total	*N* = 54	*N* = 54

*Note:* The table summarizes the full experimental layout, including diet, drug treatments, dosing schedules, and sample sizes for each group. Animals were maintained on one of three diets: 1. iron‐adequate (IA) controls (35 mg/kg Fe), 2. iron‐deficient (ID) diet (3.5 mg/kg Fe), or iron repletion (IR) diet (3.5 mg/kg Fe for 5 weeks followed by 35 mg/kg diet for 3 weeks). Rats received one of three treatment conditions: 1. vehicle (0 mg/kg, every 12 h), 2. carbidopa+L‐DOPA (5 + 20 mg/kg, every 12 h), or 3. selegiline (0.3 mg/kg, every 24 h). Each diet × treatment × sex condition included *n* = 6 rats per group, resulting in *n* = 54 males and *n* = 54 females.

### 2.3 Pharmaceutical Treatments

On PND59, animals within each diet group were all given treatments via subcutaneous injections. Vehicle (0.01% ascorbic acid, vitamin C, Sigma Aldrich, cat. no. 95210) made with 0.9% saline (Cytiva, cat. no. Z1377) was injected every 12 h. The selected doses were based on established studies proven to demonstrate an effect (Dankyi et al. 2020; Okano et al. 2019; Xie et al. 2014). Carbidopa (5 mg/kg, Sigma Aldrich, cat. no. 1095506) was administered, followed by 20 mg/kg of L‐DOPA (Sigma Aldrich, cat. no. D9628) 30 min later, and was injected every 12 h. Carbidopa was given to inhibit the decarboxylation of L‐DOPA. Selegiline (0.3 mg/kg, Millipore Sigma, cat. no. PHR3134) was administered every 24 h during the dark cycle of the room. Ascorbic acid (0.01%) was made fresh every 5 days and kept in an amber bottle at 4°C. All other drug treatments were made fresh daily, dissolved in 0.01% ascorbic acid, and kept in an amber bottle at 4°C.

### 2.4 Hematology

At five (PND59) and eight (PND76) weeks, 100 μL blood samples were collected in the morning at 09:00 via the jugular vein under 5% isoflurane anesthesia to assess hemoglobin and hematocrit levels to confirm ID or IA by standard protocols (Palsa et al. 2024). Blood was collected using anticoagulant Dipotassium (K2) EDTA to prevent clotting (RAM Scientific Safe‐T‐Fill Capillary Blood Collection, cat. no. 14‐915‐50). Hemoglobin and hematocrit were analyzed using the Heska HT5 Veterinary Hematology Analyzer (Heska Antech Company, Loveland, CO, USA).

### 2.5 End Point

Animals were euthanized on PND78 between 09:00 and 16:00. Subjects were anesthetized with ketamine (80 mg/kg) and xylazine (8 mg/kg), administered intraperitoneally, followed by transcardiac perfusion with 4°C 0.1 M phosphate‐buffered saline (~pH 7.4). Animals were then decapitated with a guillotine, followed by rapid brain collection and removal over ice. The ventral midbrain, which contains the ventral tegmentum and substantia nigra, was dissected using a standardized dissection protocol to ensure consistent tissue volume across all animals. Samples were immediately frozen on dry ice and stored at −80°C for subsequent brain iron concentrations and Western blot analysis.

### 2.6 Brain Iron Analysis

The ventral midbrain tissue was digested overnight as previously described in 1 mL of 70% trace metal grade nitric acid at 60°C followed by a 25‐fold dilution with MilliQ water (Kondaiah et al. 2021). Iron concentration (μg/g) was calculated from the total elemental iron detected in the digestion sample and normalized to protein content following complete tissue digestion. Iron concentration was determined by inductively coupled plasma atomic emission spectroscopy (ICP‐AES) (Thermo Fisher Scientific iCAP, 7400). Results reflect normalized values independent of initial tissue weight.

### 2.7 Western Blot Analysis

Western blot analysis was performed as described previously (Ferguson et al. 2021; Palsa et al. 2023). Ventral midbrain tissue homogenates were lysed in NP‐40 buffer (50 mM Tris–HCL (pH 7.4), 150 mM NaCl, 1% NP‐40, and 5 mM EDTA) and incubated on ice for 20 min before being sonicated and centrifuged at 12 000 rpm for 10 min at 4°C. The protein content of the supernatant was estimated using the micro‐BCA kit method. An equal amount of protein (30 μg) was fractionated on 4%–20% SDS‐gels under reducing conditions and transblotted onto PVDF membranes. The blots were blocked with 5% fat‐free dry milk made in TBST (tris‐buffered saline with Tween; 20 mM Tris–HCL, 150 mM NaCl, 0.1% Tween‐20) and probed overnight at 4°C for the following primary antibodies: transferrin receptor/CD71 (TfR1/CD71) (1:250; RRID:AB_1120670, Santa Cruz Biotechnology; cat. no. SC‐65882), ferroportin‐1/IREG1 (FPN1) (1:1000; RRID:AB_1619475, Alpha Diagnostics, cat. no. MTP11‐S), ferritin heavy chain (FTH1) (1:1000; Cell Signaling Technology, cat. no. 4393S), ferritin light chain (FTL) (1:1000; RRID:AB_1523609, Abcam, cat. no. ab69090), lipocalin‐2 (LCN2)/NGAL (1:200; RRID:AB_2609008, Thermo Fisher Scientific, cat. no. PA5‐46938), lipocalin‐receptor/SLC22A17 (LCN‐R) (1:1000; RRID:AB_2813262, Thermo Fisher Scientific, cat. no. PA5‐98649), glial fibrillary acidic protein (GFAP) (1:5000; RRID:AB_2792404, Thermo Fisher Scientific, cat. no. PA5‐85261), catalase (CAT) (1:2000; RRID:AB_3086611, Abcam, cat. no. ab209211), superoxide dismutase 2 (SOD2) (1:1000; RRID:AB_2191814, Santa Cruz Biotechnology; cat. no. SC‐133134), and β‐actin (1:1000; RRID:AB_476744, Sigma‐Aldrich, cat. no. A5441). Membranes were washed in TBST, and a corresponding secondary antibody conjugated to HRP was incubated for 1 h at room temperature (1:5000, RRID:AB_772210, anti‐mouse, Cytiva, cat. no. NA931; 1:5000, RRID:AB_772206, anti‐rabbit, Cytiva, cat. no. NA934). Bands were visualized using ECL reagents (RRID:AB_10188880, Santa Cruz Biotechnology, cat. no. SC‐2048) on an Amersham Imager 600 (RRID:SCR_021853, GE Amersham). The samples were run on two separate membranes, both processed and imaged concurrently to maintain consistent conditions. Identical exposure settings were used for male and female samples for each target protein. To confirm comparability between membranes, β‐actin intensities were examined across blots and were highly consistent. The images were quantified using ImageJ software (RRID:SCR_003070, NIH, Bethesda, MD, USA) and normalized to the respective loading control, β‐actin.

### 2.8 Statistical Analysis

Statistical analysis was performed using Prism software version 10.4.1 (GraphPad Software LLC., San Diego, CA, USA; RRID:SCR_002798). Data results were expressed as mean ± standard error of the mean (SEM). No outlier tests were conducted, and no data points were excluded from any analysis. All datasets were assessed for normal distribution using the Shapiro–Wilk test and met the assumption of normality, *α* = 0.05 or greater. Hemoglobin and hematocrit were analyzed using two‐way ANOVA for sex and diet as factors. Brain iron concentration was analyzed using two‐way ANOVA for sex and diet as factors as well as sex and drug. Western blot analysis was analyzed using two‐way ANOVA for sex and drug as factors with Tukey post hoc analysis to evaluate statistical differences; a *p* < 0.05 was considered significant. Full statistical results are provided in Table S1.

## 3 Results

### 3.1 Physiological Consequences of Iron Deficiency and Repletion in Male and Female Rats

**FIGURE 2:**
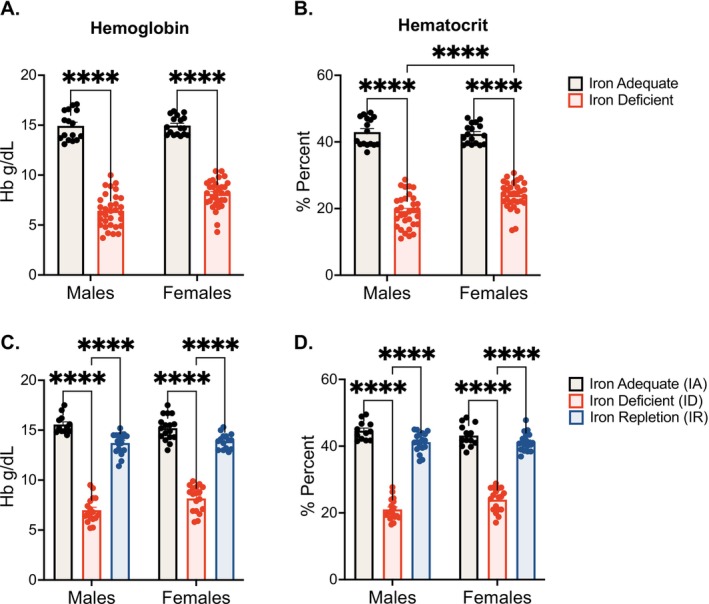
Iron deficiency and restoration confirmation via hemoglobin and hematocrit levels. (A) Mean hemoglobin levels (±SEM): Following an iron‐deficient (ID) diet, iron deficiency was confirmed in male (****p < 0.0001) and female (****p < 0.0001) rats by significantly reduced hemoglobin. (B) Mean hematocrit levels (±SEM): Iron deficiency was further confirmed in male (****p < 0.0001) and female (****p < 0.0001) rats by significantly reduced hematocrit levels. (C) Mean hemoglobin levels (±SEM): Hemoglobin levels confirmed iron restoration in the subset of rats assigned to the iron‐repletion (IR) diet conditions (male IR, *p* = 0.0740; female IR, *p* = 0.0885). (D) Mean hematocrit levels (±SEM): Hematocrit levels further confirmed iron restoration in rats assigned to the IR‐diet conditions (male IR, *p* = 0.1426; female IR *p* = 0.4266). *N* = 18 rats per group. Data were evaluated for statistical significance using two‐way ANOVA with Tukey's posttest for significance. Two‐way ANOVA results for each panel are as follows: (A: Diet *F*(1,104) = 705.3, *p* < 0.0001; sex *F*(1,104) = 6.896, *p* = 0.0099; interaction *F*(1,104) = 10.44, *p* = 0.0016. (B) Diet *F*(1,104) = 645.3, *p* < 0.0001; sex *F*(1,104) = 7.641, *p* = 0.0068; interaction *F*(1,104) = 8.321, *p* = 0.0048. (C) Diet *F*(2,102) = 502.4, *p* < 0.0001; sex *F*(1,102) = 3.515, *p* = 0.0637; interaction *F*(1,102) = 4.701, *p* = 0.0111. (D): Diet *F*(2,102) = 525.0, *p* < 0.0001; sex *F*(1,102) = 2.035, *p* = 0.1567; interaction *F*(1,102) = 3.882, *p* = 0.0237).

To investigate the effects of L‐DOPA treatment in IA, ID, and IR groups, we first confirmed ID by measuring hemoglobin and hematocrit levels. After 5 weeks (PND59) on ID diet, both male and female animals exhibited significantly reduced hemoglobin (main effect of diet: *F*(1,104) = 705.3, *p* < 0.0001) and hematocrit (main effect of diet: *F*(1,104) = 645.3, *p* < 0.0001) levels compared to the IA group, confirming deficiency (Figure 2A,B). After 3 weeks of either maintaining an IA, ID, or switching to an IR diet, animals in the IR group showed restored hemoglobin (male IR, *p* = 0.08; female IR, *p* = 0.09) and hematocrit (male IR, *p* = 0.50; female IR, *p* = 0.52) levels with measurements comparable to control levels. In contrast, hemoglobin and hematocrit values in the IR group were significantly higher than those in the ID group for both sexes (all *p* < 0.0001), indicating effective iron repletion (Figure 2C,D). A two‐way ANOVA performed on post‐treatment values confirmed a strong main effect of diet on hemoglobin (*F*(2,102) = 502.4, *p* < 0.0001) and hematocrit (*F*(2,102) = 525.0, *p* < 0.0001) during the repletion phase.

### 3.2 Ventral Midbrain Iron Concentration

**FIGURE 3:**
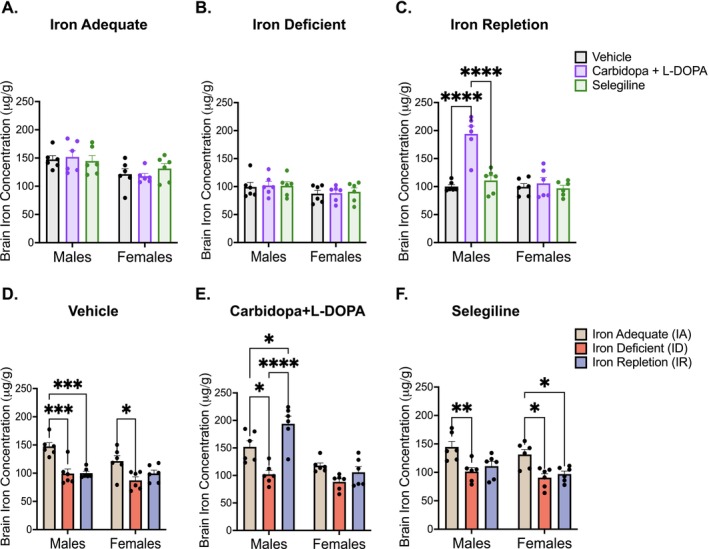
Ventral midbrain iron concentrations following antiparkinson treatments and diet conditions. (A) Mean iron concentration levels in the iron‐adequate (IA) group (± SEM): Iron concentration levels in the ventral midbrain remained consistent across all treatment groups, stratified by sex. (B) Mean iron concentration levels in the iron‐deficient (ID) group (±SEM): Iron concentration levels in the ventral midbrain remained consistent across all treatment groups, stratified by sex. (C) Mean iron concentration levels in the iron‐repletion (IR) group (±SEM): Within the male group, rats treated with carbidopa+L‐DOPA had significantly elevated brain iron concentration when compared to vehicle and selegiline‐treated groups (****p < 0.0001). (D) Mean iron concentration levels in the vehicle treatment group (±SEM): After treatment initiation, males in the IA group had significantly elevated brain iron concentration in the ventral midbrain compared to males in the ID and IR groups (***p < 0.001). Females in the IA group also had elevated brain iron concentration levels in the ventral midbrain compared to females in the ID group (*p < 0.05) but not the IR group. (E) Mean iron concentration levels in the carbidopa+L‐DOPA treatment group (±SEM): After treatment initiation, males in the IA group had significantly elevated brain iron concentration in the ventral midbrain compared to males in the ID group (*p < 0.05), whereas males in the IR group had significantly elevated brain iron concentration in the ventral midbrain compared to males in the IA group (*p < 0.05) and the ID group (****p < 0.0001). There were no differences among the female groups. (F) Mean iron concentration levels in the selegiline treatment group (±SEM): After treatment initiation, males in IA group had significantly elevated brain iron concentrations in the ventral midbrain compared to the males in the ID group (**p < 0.01). Within the females, the IA group had significantly elevated levels of brain iron concentration in the ventral midbrain compared to the ID group (*p < 0.05) and the IR group (*p < 0.05). *n* = 6 rats per group. Data were evaluated for statistical significance using two‐way ANOVA with Tukey's posttest for significance. Two‐way ANOVA results for each panel are as follows: (A: Drug *F*(2,30) = 0.0976, *p* = 0.9073; sex *F*(1,30) = 11.42, *p* = 0.002; interaction *F*(2,30) = 0.7011, *p* = 0.504. (B) Drug *F*(2,30) = 0.0797, *p* = 0.9235; sex *F*(1,30) = 4.399, *p* = 0.0445; interaction *F*(2,30) = 0.0219, *p* = 0.9784. (C) Drug *F*(2,30) = 19.94, *p* < 0.0001; sex *F*(1,30) = 22.95, *p* < 0.0001; interaction *F*(2,30) = 14.44, *p* < 0.0001. (D) Diet *F*(2,30) = 19.20, *p* < 0.0001; sex *F*(1,30) = 4.903, *p* = 0.0346; interaction *F*(2,30) = 1.586, *p* = 0.2214. (E) Diet *F*(2,30) = 17.53, *p* < 0.0001; sex *F*(1,30) = 33.92, *p* < 0.0001; interaction *F*(2,30) = 8.166, *p* = 0.0015. (F) Diet *F*(2,30) = 15.57, *p* < 0.0001; sex *F*(1,30) = 3.774, *p* = 0.0615; interaction *F*(2,30) = 0.0234, *p* = 0.9769).

To determine how brain iron content is altered by L‐DOPA and selegiline treatment under varying iron dietary conditions, total iron concentration in the ventral midbrain was quantified by ICP‐AES. A two‐way ANOVA revealed a significant effect of drug × sex in the IR group (*F*(2,30) = 14.44, *p* < 0.0001) but not in the IA (*F*(2,30) = 0.070, *p* = 0.504) and ID groups (*F*(2,30) = 0.022, *p* = 0.978). In the IA group, L‐DOPA and selegiline administration did not significantly affect brain iron levels compared to the vehicle‐treated controls (Figure 3A). In the ID group, overall brain iron content was reduced relative to the IA group in Figure 3A, but treatment condition did not further influence ventral midbrain iron content (Figure 3B). In contrast, L‐DOPA treatment in the IR group led to a significant increase in brain iron concentrations in males (*p* < 0.0001) but not in females (*p* = 0.99) (Figure 3C). When examining dietary effects within each drug treatment group, both ID and IR diets yielded reduced levels relative to IA in vehicle (*F*(2,30) = 19.20, *p* < 0.0001) and selegiline groups (*F*(2,30) = 15.57, *p* < 0.0001) for both sexes (Figure 3D,F).

A significant diet × sex interaction was observed only in the carbidopa+L‐DOPA group (*F*(2,30) = 8.17, *p* = 0.0015). Post hoc analysis revealed that male animals administered L‐DOPA on the IR diet exhibited a significant elevation in brain iron concentration, compared to their IA (*p* = 0.04) and ID (*p* < 0.0001) counterparts, whereas females did not exhibit a similar increase (Figure 3E) Consistent with this, the L‐DOPA condition also showed a main effect of diet (*F*(2,30) = 17.53, *p* < 0.0001) and sex: *F*(1,30) = 33.92, *p* < 0.0001.

### 3.3 Iron Transporter Expression

**FIGURE 4:**
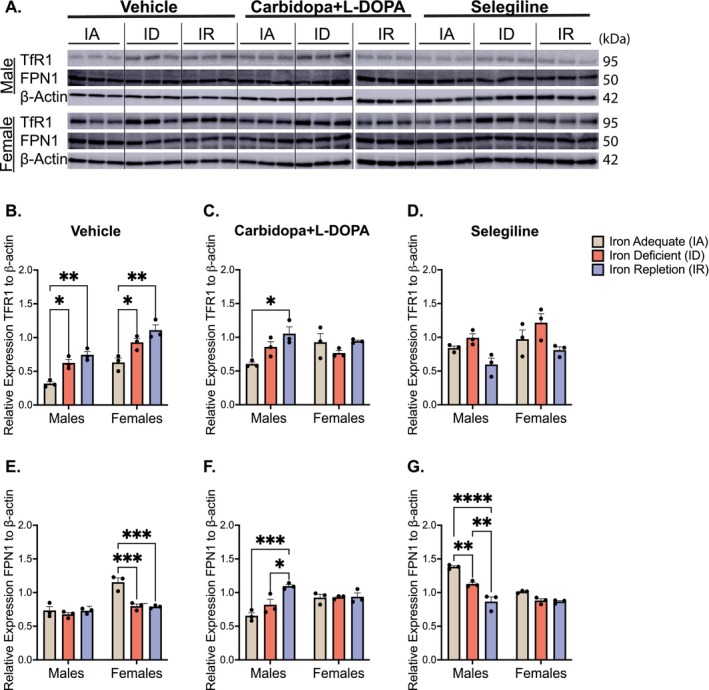
Expression of transferrin receptor 1 (TfR1) and ferroportin (FPN1) in the ventral midbrain. (A) Representative immunoblots of TfR1 and FPN1: Due to space limitations, samples were run on two separate membranes; a dotted line indicates the junction between blots. (B) Mean relative expression of TfR1 in vehicle‐treated rats (± SEM): Within the males, the iron‐adequate (IA) group had significantly less TfR1 relative expression when compared to the iron‐deficient (ID) group (*p < 0.05) and the iron‐repletion (IR) group (**p < 0.01). Within the females, the IA group also had significantly less TfR1 relative expression when compared to the ID group (*p < 0.05) and the IR group (**p < 0.01). (C) Mean relative expression of TfR1 in carbidopa+L‐DOPA treated rats (±SEM): Within the males, the IA group had significantly less TfR1 expression when compared to the IR group (*p < 0.05). There were no differences within the female groups. (D) Mean relative expression of TfR1 in selegiline treated rats (±SEM): No differences emerged across the dietary groups, stratified by sex. (E) Mean relative expression of FPN1 in vehicle‐treated rats (±SEM): Within the males, no differences emerged across the different dietary groups. Within the females, the IA group had significantly higher levels of FPN1 when compared to the ID and IR groups (***p < 0.001). (F) Mean relative expression of FPN1 in carbidopa+L‐DOPA treated rats (±SEM): Within the males, the IA group had significantly less expression of FPN1 when compared to the IR group (***p < 0.001). Additionally, the ID group had significantly less FPN1 expression when compared to the IR group (*p < 0.05). Within the females, no differences emerged. (G) Mean relative expression of FPN1 in selegiline treated rats (±SEM): Within the males, the IA group had significantly elevated levels of FPN1 expression when compared to the ID group (**p < 0.01), and the IR group (****p < 0.0001). Additionally, the ID group had high levels of FPN1 expression when compared IR group (**p < 0.01). There were no differences within the female groups. *n* = 3 rats per group and data is normalized to β‐actin. Data were evaluated for statistical significance using two‐way ANOVA with Tukey's posttest for significance. Two‐way ANOVA results for each panel are as follows: (B: Diet *F*(2,12) = 30.48, *p* < 0.0001; sex *F*(1,12) = 46.25, *p* < 0.0001; interaction *F*(2,12) = 0.162, *p* = 0.852. (C) Diet *F*(2,12) = 4.950, *p* = 0.0271; sex *F*(1,12) = 0.388, *p* = 0.545; interaction *F*(2,12) = 4.821, *p* = 0.0291. (D) Diet *F*(2,12) = 8.838, *p* = 0.0044; sex *F*(1,12) = 5.909, *p* = 0.0317; interaction *F*(2,12) = 0.143, *p* = 0.8685. (E) Diet *F*(2,12) = 14.77, *p* = 0.0006; sex *F*(1,12) = 34.86, *p* < 0.0001; interaction *F*(2,12) = 10.57, *p* = 0.0023. (F) Diet *F*(2,12) = 9.730, *p* = 0.0031; sex *F*(1,12) = 3.086, *p* = 0.104; interaction *F*(2,12) = 8.676, *p* = 0.0047. (G) Diet *F*(2,12) = 45.97, *p* < 0.0001; sex *F*(1,12) = 52.34, *p* < 0.0001; interaction *F*(2,12) = 14.80, *p* = 0.0006).

To interrogate iron uptake and export, the expression of TfR1 and FPN1, key proteins in iron uptake and export, was examined (Figure 4A). In the vehicle‐treated animals, TfR1 was significantly upregulated in the ID and IR groups compared to the IA group in both males (ID: *p* = 0.02; IR: *p* = 0.0027) and females (ID: *p* = 0.03; IR: *p* = 0.001), consistent with compensatory responses to iron deficiency and revealing a strong effect of diet (*F*(2,12) = 30.48, *p* < 0.0001; Figure 4B).

In L‐DOPA‐treated males, TfR1 expression was elevated in the animals on IR diet relative to IA, reaching statistical significance (*p* = 0.01), whereas females showed no such change (Figure 4C), diet effect: (*F*(2,12) = 4.95, *p* = 0.027). Selegiline did not alter TfR1 expression in either sex (Figure 4D). A two‐way ANOVA showed a diet × sex interaction for carbidopa+L‐DOPA (*F*(2,12) = 4.82, *p* = 0.03), whereas no interaction was detected for vehicle (*F*(2,12) = 0.162, *p* = 0.852), and selegiline (*F*(2,12) = 0.1426, *p* = 0.868).

Similarly, FPN1 expression showed sex‐ and treatment‐specific modulation. In the vehicle‐treated females, FPN1 expression was significantly reduced in ID (*p* = 0.0007) and IR (*p* = 0.0005) diets compared to IA, with no differences observed in males (Figure 4E, diet effect: *F*(2,12) = 14.77, *p* = 0.0006). In the L‐DOPA‐treated males, FPN1 was significantly increased in the IR group relative to IA (*p* = 0.0007) and ID (*p* = 0.02), while females showed no significant changes (Figure 4F, diet effect: *F*(2,12) = 9.73, *p* = 0.0031). Selegiline treatment led to reduced FPN1 expression in ID (*p* = 0.0024) and IR (*p* < 0.0001) male rats, but not in females (Figure 4G, diet effect: *F*(2,12) = 45.97, *p* < 0.0001). Consistent with these patterns, a two‐way ANOVA showed a diet × sex interaction for vehicle (*F*(2,12) = 10.57, *p* = 0.0023), carbidopa+L‐DOPA (*F*(2,12) = 8.68, *p* = 0.0047), and selegiline (*F*(2,12) = 14.80, *p* = 0.0006).

### 3.4 Iron Storage Expression in Response to L‐DOPA Treatment

**FIGURE 5:**
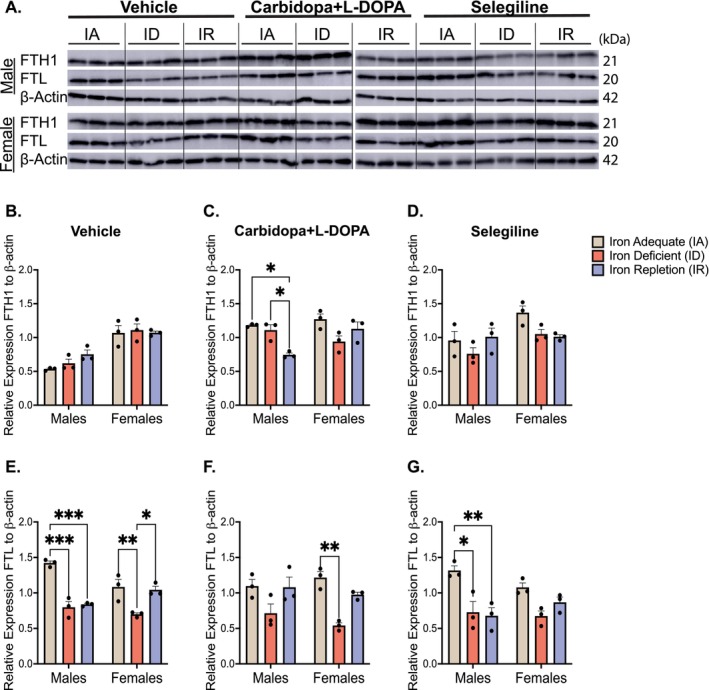
Expression of ferritin heavy chain (FTH1) and ferritin light chain (FTL) in the ventral midbrain. (A) Representative immunoblots of FTH1 and FTL: Due to space limitations, samples were run on two separate membranes; a dotted line indicates the junction between blots. (B) Mean relative expression of FTH1 in vehicle‐treated rats (±SEM): Among the male and female groups, there were no differences in FTH1 expression across the dietary conditions. (C) Mean relative expression of FTH1 in carbidopa+L‐DOPA treated rats (±SEM): Within the males, the iron‐adequate (IA) group had elevated levels of FTH1 in the ventral midbrain when compared to the iron‐repletion (IR) group (*p < 0.05). Additionally, the iron‐deficient (ID) group had elevated levels of FTH1 when compared to the IR group (*p < 0.05). There were no differences within the female groups. (D) Mean relative expression of FTH1 in selegiline treated rats (±SEM): There were no differences among the dietary groups for males or females. (E) Mean relative expression of FTL in vehicle‐treated rats (±SEM): Within the males, the IA group had elevated relative expression of FTL when compared to the ID and IR groups (***p < 0.001). Within the females, the IA group had a higher level of expression of FTL when compared to the ID group (**p < 0.01), and the IR group also had a higher level of FTL expression when compared to the ID group (*p < 0.05). (F) Mean relative expression of FTL in carbidopa+L‐DOPA treated rats (±SEM): Within the males, differences among the three different dietary groups did not emerge. Within the females, the IA group had significantly elevated levels of FTL expression when compared to the ID group (**p < 0.001). (G) Mean relative expression of FTL in selegiline treated rats (±SEM): Within the males, the IA group had a higher level of FTL expression when compared to the ID (*p < 0.05) and IR groups (**p < 0.01). There were no differences within the female groups. *n* = 3 rats per group and data is normalized to β‐actin. Data were evaluated for statistical significance using two‐way ANOVA with Tukey's posttest for significance. Two‐way ANOVA results for each panel are as follows: (B: Diet *F*(2,12) = 1.21, *p* = 0.332; sex *F*(1,12) = 59.84, *p* < 0.0001; interaction *F*(2,12) = 1.29, *p* = 0.3108. (C) Diet *F*(2,12) = 8.379, *p* = 0.0053; sex *F*(1,12) = 2.8676, *p* = 0.1157; interaction *F*(2,12) = 7.274, *p* = 0.0085. (D) Diet *F*(2,12) = 3.285, *p* = 0.0728; sex *F*(1,12) = 8.198, *p* = 0.0143; interaction *F*(2,12) = 2.20, *p* = 0.1535. (E) Diet *F*(2,12) = 34.88, *p* < 0.0001; sex *F*(1,12) = 2.415, *p* = 0.1461; interaction *F*(2,12) = 9.953, *p* = 0.0028. (F): Diet *F*(2,12) = 15.92, *p* = 0.0004; sex *F*(1,12) = 0.438, *p* = 0.5206; interaction *F*(2,12) = 1.224, *p* = 0.328. (G) Diet *F*(2,12) = 15.41, *p* = 0.0005; sex *F*(1,12) = 0.192, *p* = 0.6689; interaction *F*(2,12) = 2.479, *p* = 0.1256).

FTH1 and FTL were quantified as indicators of intracellular iron storage (Figure 5A). While FTH1 catalyzes the ferroxidase reaction to convert Fe^2+^ to Fe^3+^ for safe sequestration, FTL stabilizes the ferritin nanocage and increases its overall iron‐loading capacity, becoming an important determinant of the cell's ability to store iron and buffer increases in labile iron. In the vehicle‐ and selegiline‐treated groups, FTH1 expression remained stable across diets in both sexes, showing no effect of diet (Figure 5B,D). However, L‐DOPA‐treated males on the IR diet showed significantly reduced FTH1 compared to IA (*p* = 0.01) and ID (*p* = 0.03) groups (Figure 5C, diet effect: *F*(2,12) = 8.38, *p* = 0.0053). Notably, IA males treated with either L‐DOPA or selegiline (Figure 5C,D) displayed approximately twice the FTH1 expression observed in the vehicle‐treated IA males (Figure 5B). A two‐way ANOVA revealed a significant diet × sex interaction for carbidopa+L‐DOPA (*F*(2,12) = 7.27, *p* = 0.0085), but no interaction for vehicle (*F*(2,12) = 1.29, *p* = 0.311) and selegiline (*F*(2,12) = 2.20, *p* = 0.154).

For FTL, vehicle‐treated males and females on the ID diet showed reduced expression versus IA. Interestingly, IR males had lower FTL than IA (*p* = 0.0002), while IR females exhibited increased FTL compared to ID (*p* = 0.02; Figure 5E). In L‐DOPA‐treated females, ID diet was associated with reduced FTL expression versus IA (*p* = 0.0039), with no differences observed in males (Figure 5F). In the selegiline treatment group, FTL expression was significantly lower in ID (*p* = 0.01) and IR (*p* = 0.0055) males, while female expression remained unaffected (Figure 5G). A two‐way ANOVA confirmed a significant diet × sex interaction for vehicle (*F*(2,12) = 9.95, *p* = 0.0028), whereas no interaction was detected for carbidopa+L‐DOPA (*F*(2,12) = 1.22, *p* = 0.328) and selegiline (*F*(2,12) = 2.48, *p* = 0.126).

### 3.5 Lipocalin‐2 Pathway and Astrocyte Reactivity

**FIGURE 6:**
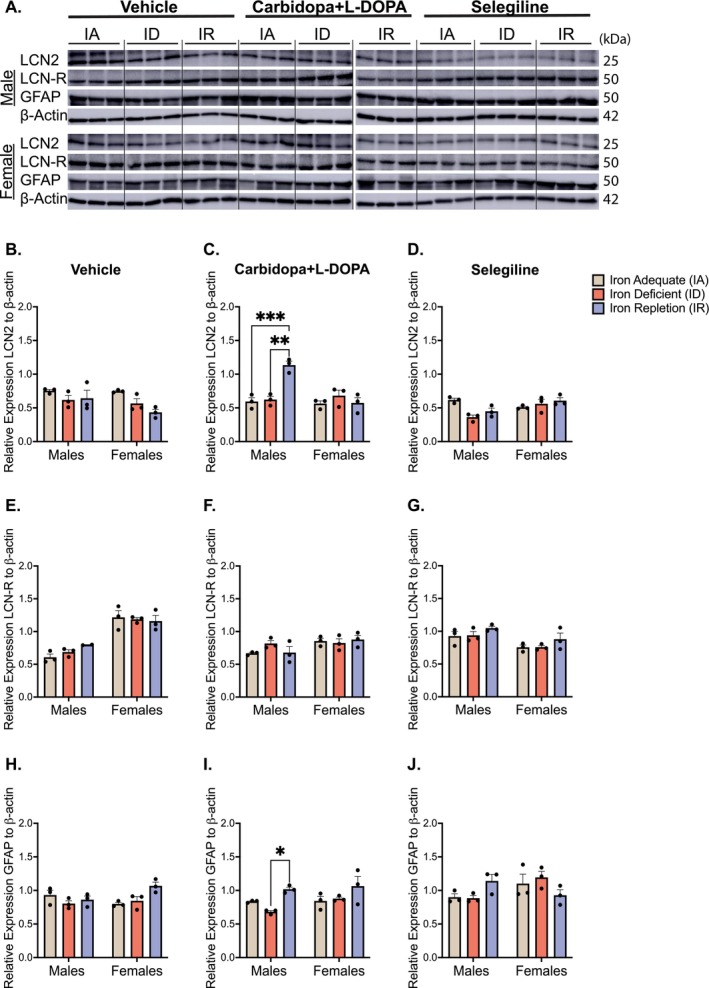
Expression of lipocalin‐2 (LCN2), lipocalin‐receptor/SLC22A17 (LCN‐R), and glial fibrillary acidic protein (GFAP) in the ventral midbrain. (A) Representative immunoblots of LCN2, LCN‐R, and GFAP: Due to space limitations, samples were run on two separate membranes; a dotted line indicates the junction between blots. (B) Mean relative expression of LCN2 in vehicle‐treated rats (±SEM): There were no differences within the male and female groups. (C) Mean relative expression of LCN2 in carbidopa+L‐DOPA treated rats (±SEM): Within the males, the iron‐repletion (IR) group had a higher level of LCN2 expression when compared to the iron‐adequate (IA) (***p < 0.001) and iron‐deficient (ID) (**p < 0.01) groups. There were no differences within the females. (D) Mean relative expression of LCN2 in selegiline treated rats (±SEM): No differences emerged across the male or female dietary groups. (E–G) Mean relative expression of LCN‐R in vehicle, carbidopa+L‐DOPA, or selegiline treated rats, respectively (±SEM): No differences emerged between the male or female dietary groups across any treatment condition. (H) Mean relative expression of GFAP in vehicle‐treated rats (±SEM): There were no differences in GFAP expression within males or females, regardless of dietary condition. (I) Mean relative expression of GFAP in carbidopa+L‐DOPA treated rats: Within the males, the IR group had significantly higher levels of GFAP expression when compared to the ID group (*p < 0.05). There were no differences among the female groups. (J) Mean relative expression of GFAP in selegiline treated rats: There were no differences within the male or female groups across the dietary conditions. *n* = 3 rats per group and data is normalized to β‐actin. Data were evaluated for statistical significance using two‐way ANOVA with Tukey's posttest for significance. Two‐way ANOVA results for each panel are as follows: (B: Diet *F*(2,12) = 5.437, *p* = 0.0208; sex *F*(1,12) = 2.653, *p* = 0.1293; interaction *F*(2,12) = 1.251, *p* = 0.3211. (C) Diet *F*(2,12) = 9.851, *p* = 0.0029; sex *F*(1,12) = 11.43, *p* = 0.0055; interaction *F*(2,12) = 13.60, *p* = 0.0008. (D) Diet *F*(2,12) = 2.916, *p* = 0.0929; sex *F*(1,12) = 5.501, *p* = 0.037; interaction *F*(2,12) = 7.49, *p* = 0.0077. (E) Diet *F*(2,12) = 0.4633, *p* = 0.6409; sex *F*(1,12) = 76.76, *p* < 0.0001; interaction *F*(2,12) = 1.58, *p* = 0.2494. (F) Diet *F*(2,12) = 0.5209, *p* = 0.6068; sex *F*(1,12) = 7.114, *p* = 0.0205; interaction *F*(2,12) = 1.594, *p* = 0.2433. (G) Diet *F*(2,12) = 2.686, *p* = 0.1086; sex *F*(1,12) = 12.60, *p* = 0.004; interaction *F*(2,12) = 0.005, *p* = 0.9946. (H) Diet *F*(2,12) = 3.324, *p* = 0.071; sex *F*(1,12) = 0.7235, *p* = 0.4117; interaction *F*(2,12) = 4.559, *p* = 0.0337. (I) Diet *F*(2,12) = 7.761, *p* = 0.0069; sex *F*(1,12) = 2.119, *p* = 0.1711; interaction *F*(2,12) = 1.045, *p* = 0.3816. (J) Diet *F*(2,12) = 0.1137, *p* = 0.8935; sex *F*(1,12) = 1.802, *p* = 0.2043; interaction *F*(2,12) = 4.628, *p* = 0.0324).

LCN2 expression was unchanged in both vehicle and selegiline‐treated animals (Figure 6B,D). However, males on the IR diet in the L‐DOPA‐treated group showed a significant increase in LCN2 expression (*p* = 0.0007; Figure 6C). A two‐way ANOVA confirmed a significant diet × sex interaction exclusively for carbidopa+L‐DOPA (*F*(2,12) = 13.60, *p* = 0.0008). Despite LCN2 elevation, its receptor SLC22A17, also named LCN‐receptor (LCN‐R), showed no significant differences across any treatment or dietary groups (Figure 6E–G), and no diet × sex interaction was detected. Additionally, since astrocytes are the primary producers of LCN2, GFAP expression was examined and found to be significantly elevated in L‐DOPA‐treated males on the IR diet (*p* = 0.04), suggesting astrocytic activation in this group (Figure 6I), although no diet × sex interaction was observed.

### 3.6 Oxidative Stress in Response to L‐DOPA Treatment

**FIGURE 7:**
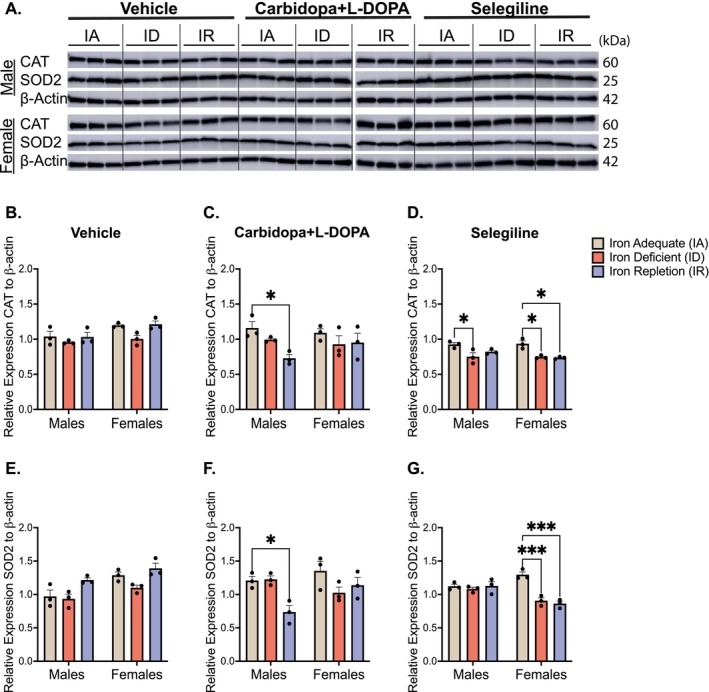
Expression of catalase (CAT) and superoxide dismutase 2 (SOD2) in the ventral midbrain. (A) Representative immunoblots of CAT and SOD2: Due to space limitations, samples were run on two separate membranes; a dotted line indicates the junction between blots. (B) Mean relative expression of CAT in vehicle‐treated rats (±SEM): There were no differences among the dietary groups, stratified by sex. (C) Mean relative expression of CAT in carbidopa+L‐DOPA treated rats (±SEM): Within the males, the iron‐adequate (IA) group had significantly higher expression of CAT when compared to the iron‐repletion (IR) group (*p < 0.05). There were no differences within the female groups. (D) Mean relative expression of CAT in selegiline treated rats (±SEM): Within the males, the IA group had significantly elevated CAT expression when compared to the iron‐deficient (ID) group (*p < 0.05). Within the females, the IA group had significantly elevated CAT expression when compared to the ID and IR groups (*p < 0.05). (E) Mean relative expression of SOD2 in vehicle‐treated rats (±SEM): There were no differences within males or females, regardless of dietary condition. (F) Mean relative expression of SOD2 in carbidopa+L‐DOPA treated rats (±SEM): Within the males, the IA group had significantly higher expression of SOD2 when compared to the IR group (*p < 0.05). There were no differences within the female groups. (G) Mean relative expression of SOD2 in the selegiline treated rats (±SEM): There were no differences within the male groups. Within the females, the IA group had significantly higher expression of SOD2 when compared to the ID and IR groups (***p < 0.001). *n* = 3 rats per group and data is normalized to β‐actin. Data were evaluated for statistical significance using two‐way ANOVA with Tukey's posttest for significance. Two‐way ANOVA results for each panel are as follows: (B: Diet *F*(2,12) = 4.931, *p* = 0.0274; sex *F*(1,12) = 9.427, *p* = 0.0097; interaction *F*(2,12) = 0.968, *p* = 0.4076. (C) Diet *F*(2,12) = 4.979, *p* = 0.0266; sex *F*(1,12) = 1.567, *p* = 0.6992; interaction *F*(2,12) = 1.714, *p* = 0.2214. (D) Diet *F*(2,12) = 17.27, *p* = 0.0003; sex *F*(1,12) = 0.9561, *p* = 0.3475; interaction *F*(2,12) = 1.238, *p* = 0.3246. (E) Diet *F*(2,12) = 9.673, *p* = 0.0031; sex *F*(1,12) = 16.79, *p* = 0.0015; interaction *F*(2,12) = 0.8255, *p* = 0.4514. (F) Diet *F*(2,12) = 6.027, *p* = 0.0154; sex *F*(1,12) = 2.041, *p* = 0.1786; interaction *F*(2,12) = 4.69, *p* = 0.0313. (G) Diet *F*(2,12) = 16.35, *p* = 0.0004; sex *F*(1,12) = 6.42, *p* = 0.0262; interaction *F*(2,12) = 14.25, *p* = 0.0007).

To evaluate oxidative stress, CAT and SOD2 expression were assessed (Figure 7A). Neither enzyme was altered in vehicle‐treated groups (Figure 7B,E). In contrast, L‐DOPA‐treated males on an IR diet exhibited significantly decreased CAT (*p* = 0.05) and SOD2 (*p* = 0.05) levels relative to IA controls, while females remained unaffected (Figure 7C), diet effect: (*F*(2,12) = 4.97, *p* = 0.027); Figure 7F, diet effect: (*F*(2,12) = 6.03, *p* = 0.015). In the selegiline group, both CAT and SOD2 were significantly reduced in ID (CAT, *p* = 0.02; SOD2, *p* = 0.0004) and IR (CAT, *p* = 0.01; SOD2, *p* = 0.0001) females, while CAT was reduced in ID males (*p* = 0.03; Figure 7D, diet effect: *F*(2,12) = 17.27, *p* = 0.0003); Figure 7G, diet effect: (*F*(2,12) = 16.35, *p* = 0.0004). A two‐way ANOVA revealed no significant diet × sex interaction for CAT. However, for SOD2, a significant diet × sex interaction was detected for carbidopa+L‐DOPA (*F*(2,12) = 4.69, *p* = 0.03) and selegiline groups (*F*(2,12) = 14.25, *p* = 0.0007).

## 4 Discussion

This study demonstrates that L‐DOPA treatment under IR conditions results in increased iron accumulation in the ventral midbrain, specifically in male rats, accompanied by dysregulation of iron transport and storage proteins, elevated astrocyte reactivity, and oxidative stress. In contrast, selegiline did not significantly affect brain iron levels across dietary conditions. These findings provide a mechanistic framework linking systemic iron status to brain iron homeostasis during dopaminergic treatment and uncover critical sex‐specific vulnerabilities.

Our findings expand upon prior clinical and pre‐clinical work, suggesting a complex relationship between iron metabolism and L‐DOPA. Earlier studies showed that PD patients exhibited elevated iron in the substantia nigra, but only following long‐term L‐DOPA therapy (Du et al. 2022; Song et al. 2021). Additionally, a history of iron deficiency anemia is more common in PD patients and has been strongly associated with an increased risk of developing PD, with ID individuals showing a 1.5‐fold higher risk relative to matched controls (Hong et al. 2016; Savica et al. 2009). Our data corroborate these observations in a translational animal model but suggest that iron accumulation may be limited to male patients with iron deficiency who are subsequently repleted for their ID while being treated with L‐DOPA. A critical point is that the IR groups do not represent a steady‐state iron‐adequate condition. Instead, they reflect a post‐deficiency compensatory phase in which iron metabolism is fundamentally altered due to the preceding 5‐week iron deficiency period. As a result, IR animals maintain an iron‐handling profile that is distinct from the IA animals. In male L‐DOPA‐treated animals, the regulatory systems do not return to baseline after repletion but instead show altered iron‐handling proteins with upregulation of FPN1 and LCN2, and reduced expression of FTH1 and diminished antioxidant capacity (CAT and SOD2) relative to the IA males. These changes indicate that L‐DOPA interacts and disrupts the sensitized IR brain, which remains in a heightened “iron demand” state, even after the dietary iron has been normalized. Importantly, while iron deficiency reduced brain iron levels, repletion combined with L‐DOPA selectively increased iron accumulation in males, a phenomenon not observed in females. In females, the IA‐ID difference was narrower, possibly reducing the stimulus for overcorrection that was observed in males. This indicates that females maintain a tighter physiological control of ventral midbrain iron, which may make them less sensitive to L‐DOPA‐induced iron accumulation after repletion, highlighting a male and female difference in iron regulation. This sex‐dependent susceptibility aligns with epidemiological data reporting higher PD prevalence and iron‐related oxidative damage in males (Cerri et al. 2019; Dorsey et al. 2018; Rozani et al. 2019).

Mechanistically, the iron accumulation appears to be mediated by altered regulation of key iron transport proteins. We found that L‐DOPA treatment in IR males increased expression of TfR1 and FPN1, suggesting an imbalance between iron uptake and export. Previous studies reported that TfR1 is upregulated in response to neuronal iron demand and oxidative stress (Leitner and Connor 2012; Palsa et al. 2023; Petralla et al. 2024), and our findings support this in a context where dopamine synthesis, which is dependent on iron via tyrosine hydroxylase, is pharmacologically stimulated via administration of L‐DOPA. Elevated FPN1 expression may reflect a compensatory attempt to limit intracellular iron toxicity (Troadec et al. 2010). In males, selegiline reduced FPN1 expression in both ID and IR groups despite no increase in brain iron concentration. This pattern suggests that selegiline modulates FPN1 indirectly through astrocytic iron management rather than by altering neuronal iron overload. Astrocytes express high levels of FPN1 in the midbrain and can downregulate FPN1 to retain iron when buffering oxidative stress or supporting neighboring neurons (Dong‐Chen et al. 2023). Selegiline is known to promote neuroprotective features and reduce astrocyte‐derived oxidative stress, which may shift astrocytes toward greater iron sequestration (You et al. 2022). Thus, FPN1 reduction likely reflects a compensatory astrocyte‐driven adjustment in iron handling rather than pathological accumulation, supporting selegiline's overall preservation of iron homeostasis across dietary conditions. Furthermore, reduced expression of FTH1 in L‐DOPA‐treated IR males suggests impaired iron storage capacity, compounding intracellular iron availability and toxicity. Since FTH1 is predominantly expressed in neurons, its reduction likely reflects dopaminergic neuronal dysregulation (Chiou et al. 2020; Connor et al. 1995). One possible explanation is that dopamine‐induced oxidative stress, exacerbated by iron, may suppress FTH1 expression or destabilize ferritin, thereby reducing the ability to buffer excess iron (Buoso et al. 2024; Tian et al. 2020). These data align with earlier reports that low ferritin expression exacerbates iron‐mediated oxidative stress and neuronal death (Connor et al. 1995; Han et al. 2000; Koziorowski et al. 2007).

Interestingly, LCN2 is often implicated in neuroinflammation and iron trafficking during neurodegenerative stress (Fan et al. 2024; Ferreira et al. 2018; Kim et al. 2016). Our study shows L‐DOPA significantly increased LCN2 expression only in IR males, consistent with a profile that would support neurodegeneration (Dekens et al. 2021; Kim et al. 2016; Liu et al. 2024). Moreover, the lack of change in its receptor SLC22A17/LCN‐R suggests a possible saturation or downregulation of LCN2‐mediated iron import, potentially due to chronic ligand exposure or receptor in response to elevated extracellular LCN2. LCN2 is known to be produced by astrocytes in response to elevated iron levels and oxidative stress and acts by binding siderophores to regulate iron availability (Schröder et al. 2023; Yang et al. 2002). Increased GFAP expression in this group further suggests that astrocyte activation is an early marker of iron‐induced stress, consistent with previous studies that show reactive astrocytes release pro‐inflammatory mediators in response to iron overload and contribute to neuroinflammation (Kim et al. 2016; Macco et al. 2013). Oxidative stress markers CAT and SOD2 were significantly decreased in L‐DOPA‐treated IR males, indicating impaired antioxidant defenses and increased vulnerability to oxidative damage. This impaired defense may reflect mitochondrial dysfunction, dopaminergic neurotoxicity, or transcriptional repression in the context of excessive iron and dopamine metabolism. Both of which can exacerbate oxidative damage through Fenton chemistry and the formation of toxic byproducts when dopamine is oxidized into reactive quinones (Melin et al. 2015; Zhou et al. 2010). These findings are consistent with reports that long‐term L‐DOPA treatment exacerbates oxidative damage in iron‐rich brain regions (Hörmann et al. 2021; Stansley and Yamamoto 2013). This response was sex‐specific, with L‐DOPA‐treated IR males showing pronounced oxidative stress, whereas females exhibited more stable iron regulation and antioxidant expression. The more stable regulation of iron uptake is consistent with reports of sex‐bias uptake following dietary iron restriction during development (Hahn et al. 2009; Palsa et al. 2024). The factors involved in this regulation have yet to be elucidated, but it is clear that females do not lose brain iron when there is a dietary challenge. Estrogen or other sex‐linked regulatory factors may underlie these protective effects in females (Lee et al. 2019; Siani et al. 2017). While this hypothesis remains speculative, supporting evidence comes from a study showing increased iron depositions in the brain in estrogen‐deficient mice (Shin et al. 2020). In humans, pre‐menopausal women who have undergone a hysterectomy and post‐menopausal women are at higher risk for developing PD compared to their control counterparts (Brinton et al. 2015; Currie et al. 2004; Ibrahim et al. 2022), suggesting that estrogen may play a critical role in maintaining iron homeostasis and preventing its dysregulation.

The concurrent downregulation of FTH1, CAT, and SOD2 suggests a collapse in multiple protective pathways. FTH1, which stores iron in a non‐reactive form, is critical for buffering intracellular iron. Its reduction implies that neurons and potentially astrocytes are failing to sequester excess iron. This failure likely amplifies redox stress, depleting antioxidant reserves. Notably, astrocytes, which typically play a key role in iron buffering and neuroprotection, may become overwhelmed in this context. Prior studies have shown that astrocytes upregulate iron‐handling proteins and antioxidant enzymes in response to iron overload (Macco et al. 2013; Pelizzoni et al. 2013), but excessive or sustained iron influx, especially when combined with dopamine metabolism, can impair astrocytic function (Wise et al. 2022). In our model, the combination of iron repletion and L‐DOPA may push astrocytes beyond their regulatory capacity, resulting in inadequate support for neighboring neurons. Moreover, chronic exposure to iron and dopamine oxidation products may compromise transcriptional and translational control of antioxidant enzymes, further weakening cellular defenses.

A key strength of this study is the use of a physiologically relevant animal model that reflects a clinically common scenario of PD patients with coexisting iron deficiency who receive iron supplementation concurrently with dopaminergic therapy. This study design allowed us to isolate the interaction between systemic iron status and anti‐PD treatments, particularly L‐DOPA and selegiline, in shaping brain iron homeostasis. By including both sexes and examining multiple iron‐related and neuroinflammatory proteins, our data suggest previously underrecognized sex‐specific regulatory mechanisms that influence brain iron handling in the ventral midbrain. Future work will integrate this dietary paradigm with PD models to examine behavioral outcomes and determine whether iron accumulation modifies the functional response to anti‐PD treatments. Such studies will help bridge the mechanistic insights presented here with clinically relevant motor features. While our findings provide novel insights, several limitations must be acknowledged. Notably, our animal model did not include an induced Parkinsonian phenotype. Although this allowed us to isolate the effects of iron status and treatment on brain iron metabolism without the confounding influence of neurodegeneration, it does limit the direct translational applicability to PD pathology. However, while existing genetic mouse models offer valuable insights into PD mechanisms, they possess notable limitations and often fail to fully replicate the hallmark neuropathology of PD, including selective dopaminergic neuron loss, α‐synuclein aggregation, and robust iron accumulation in the substantia nigra. When iron accumulation does occur, it tends to be modest and often requires additional stressors such as neurotoxins or aging to elicit PD‐like pathology (Benskey et al. 2016; Dovonou et al. 2023; Jia et al. 2018; Mochizuki and Yasuda 2012; Trancikova et al. 2011). The limitations highlight the need for improved preclinical models that reliably capture iron‐related pathology. Our dietary model addresses this gap by enabling investigation of iron supplementation and L‐DOPA treatment in the context of altered system iron status.

In conclusion, our study reveals that dietary iron repletion during L‐DOPA treatment promotes sex‐specific dysregulation of brain iron homeostasis. These results offer mechanistic insights into how iron supplementation may exacerbate neurodegeneration in PD and underscore the importance of considering iron status and sex when designing therapeutic strategies. Selegiline by contrast, appears to preserve iron homeostasis and may offer neuroprotective advantages in iron‐compromised individuals. These findings have significant implications for personalized clinical strategies in PD, including managing iron deficiency in PD patients and support the development of sex and iron status therapeutic interventions.

## Author Contributions

**Rebecka O. Serpa:** conceptualization, formal analysis, funding acquisition, investigation, methodology, project administration, visualization, writing – original draft, writing – review and editing. **Emily Tufano:** formal analysis, investigation, visualization, writing – review and editing. **Kondaiah Palsa:** investigation, methodology, writing – review and editing. **Timothy B. Helmuth:** investigation, writing – review and editing. **Sara Mills‐Huffnagle:** investigation, writing – review and editing. **Mathias Kant:** investigation, writing – review and editing. **James R. Connor:** conceptualization, funding acquisition, methodology, writing – original draft, writing – review and editing.

## Funding

This work was supported by National Institute of Neurological Disorders and Stroke, F31‐NS137783, R01‐NS113912‐05. National Center for Advancing Translational Sciences, TL1‐TR002016.

## Conflicts of Interest

The authors declare no conflicts of interest.

## Supporting information


**Appendix S1:** jnc70389‐sup‐0001‐AppendixS1.pdf.

**Table S1:** jnc70389‐sup‐0002‐TableS1.xlsx.

## Data Availability

The data that support the findings of this study are available from the corresponding author upon reasonable request.
