# Supplementary material for: Sex and Diet Biased Effect of L‐DOPA on Iron Accumulation in the Ventral Midbrain
Source: J Neurochem. 2026 Mar 5;170(3):e70389. doi: 10.1111/jnc.70389 (PMC12963950; doi:10.1111/jnc.70389)
Supplement: Supplementary file 1 — Appendix S1: jnc70389‐sup‐0001‐AppendixS1.pdf. [file JNC-170-0-s001.pdf]

Sex and Diet Biased Effect of L-DOPA on Iron Accumulation in the Ventral Midbrain

Rebecka O. Serpa[1,2,3*], Emily Tufano[1], Kondaiah Palsa[1], Timothy B. Helmuth[1], Sara Mills-Huffnagle[2,3], Mathias Kant[1], & James R. Connor[1,2,3*]

[1] Department of Neurosurgery, The Pennsylvania State University College of Medicine, Hershey, PA, USA
[2] Department of Neuroscience and Experimental Therapeutics Sciences, The Pennsylvania State University College of Medicine, Hershey, PA, USA
[3] Penn State Neuroscience Institute, The Pennsylvania State University College of Medicine, Hershey, PA, USA

***Co-corresponding Authors:**
James R. Connor, PhD
jconnor@pennstatehealth.psu.edu

Rebecka O. Serpa
rserpa@pennstatehealth.psu.edu

Uncropped Western Blot Data: For all images (Figures 4-7), the first half of the samples are shown on the top membrane (membrane 1) and the second half on the bottom (membrane 2).

Figure 4: Ferroportin (FPN1) & Transferrin Receptor 1 (TfR1) Images

Figure 4A: Female TfR1

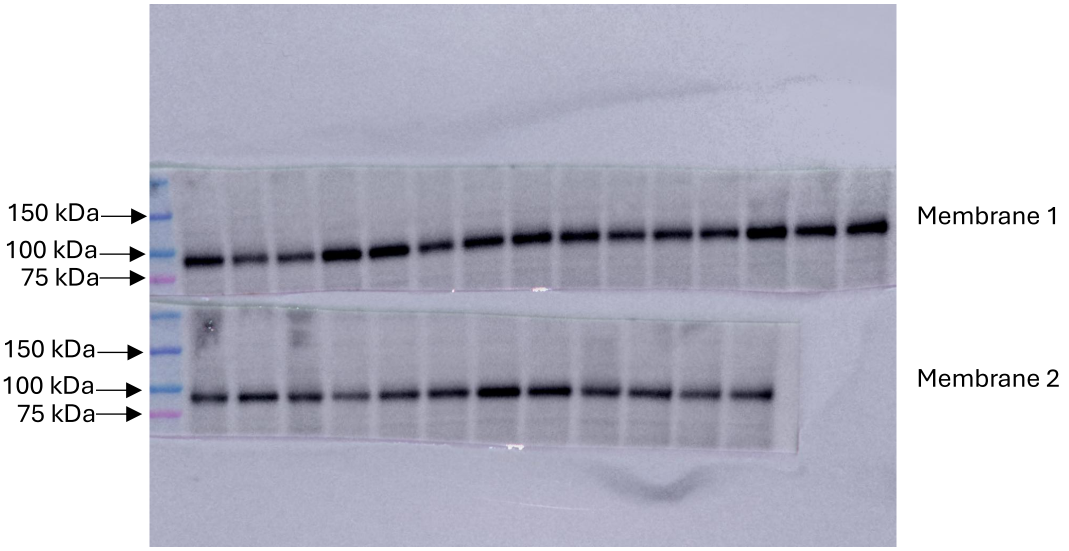

Figure 4A: Female FPN1

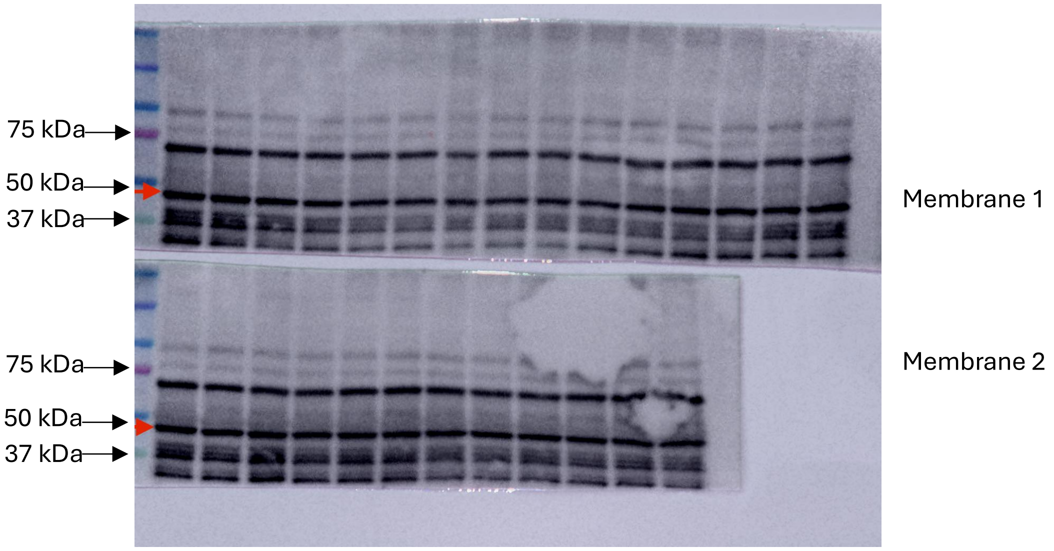

Figure 4A: Female ß-actin

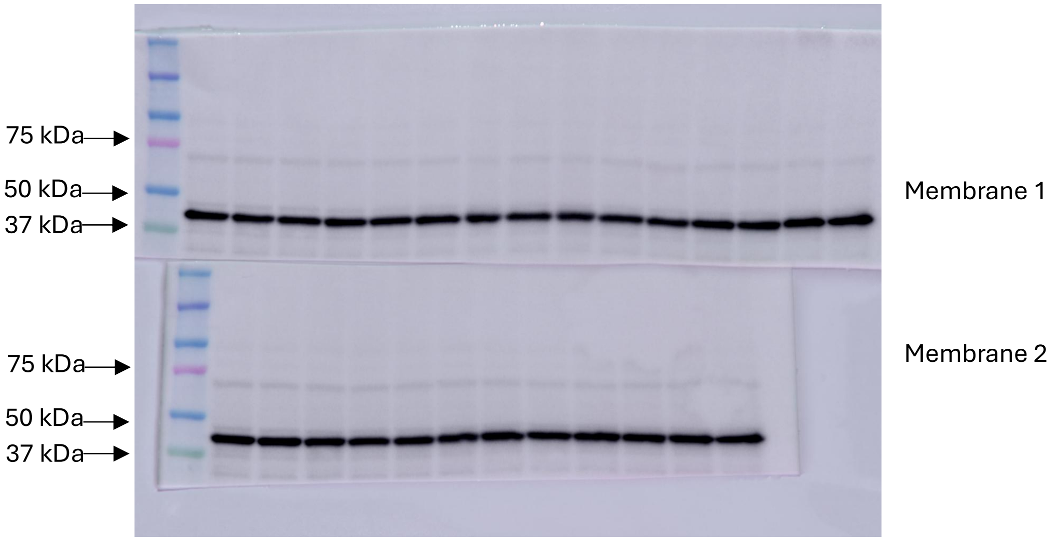

Figure 4A: Male TfR1

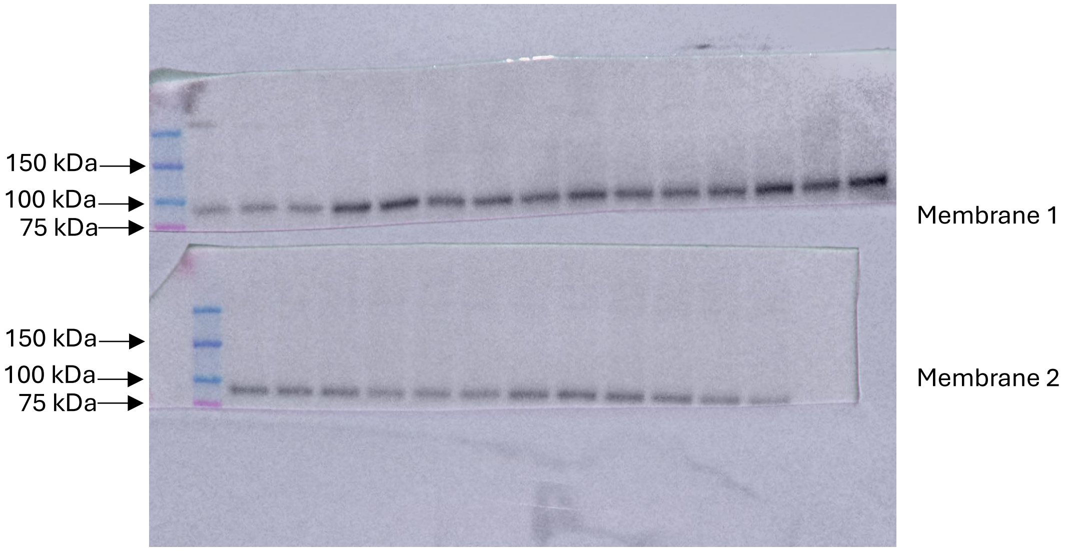

Figure 4A: Male FPN1

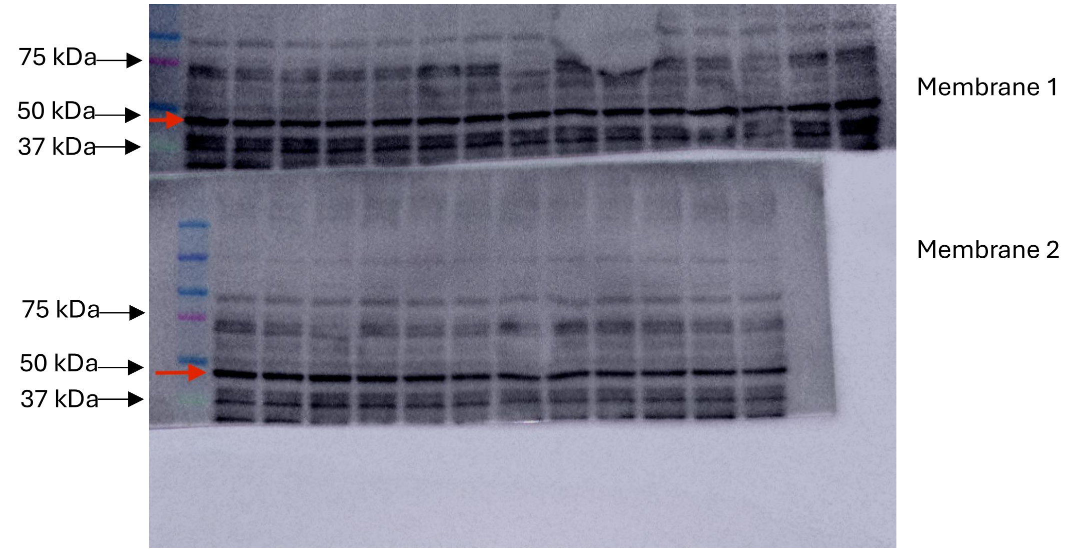

Figure 4A: Male ß-actin

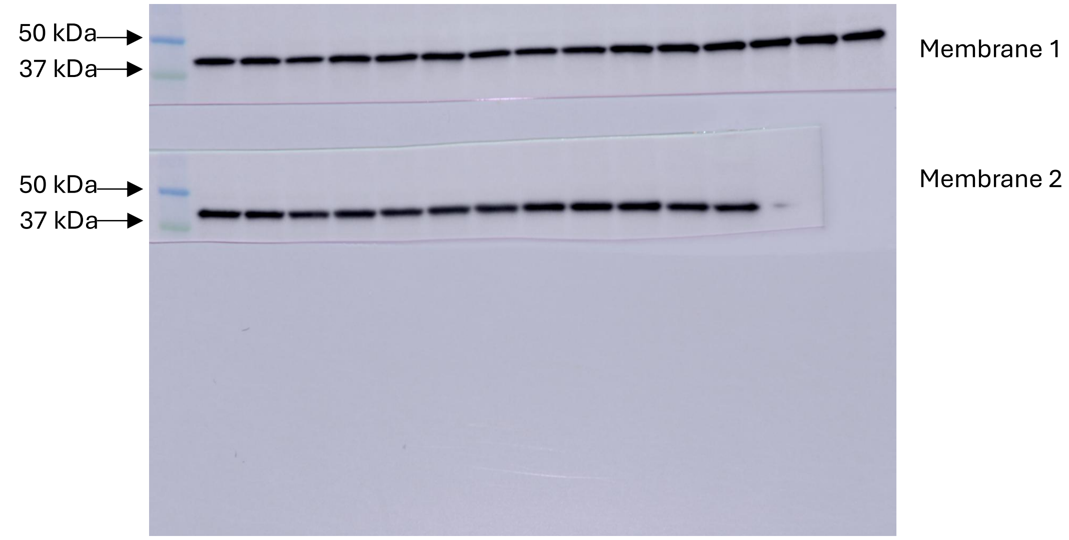

Figure 5: Ferritin Heavy Chain (FTH1) & Ferritin Light Chain (FTL) Images

Figure 5A: Female FTH1

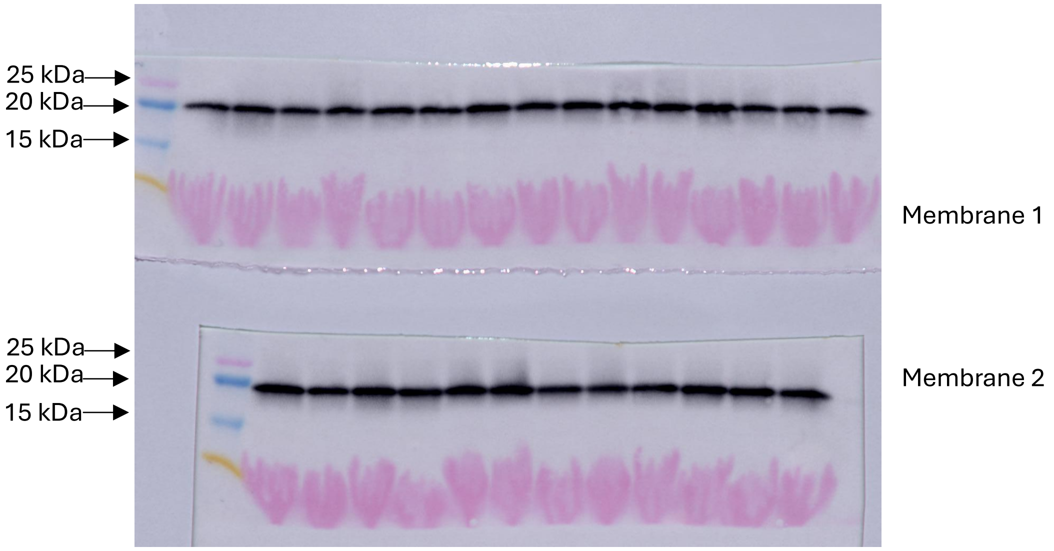

Figure 5A: Female FPN1

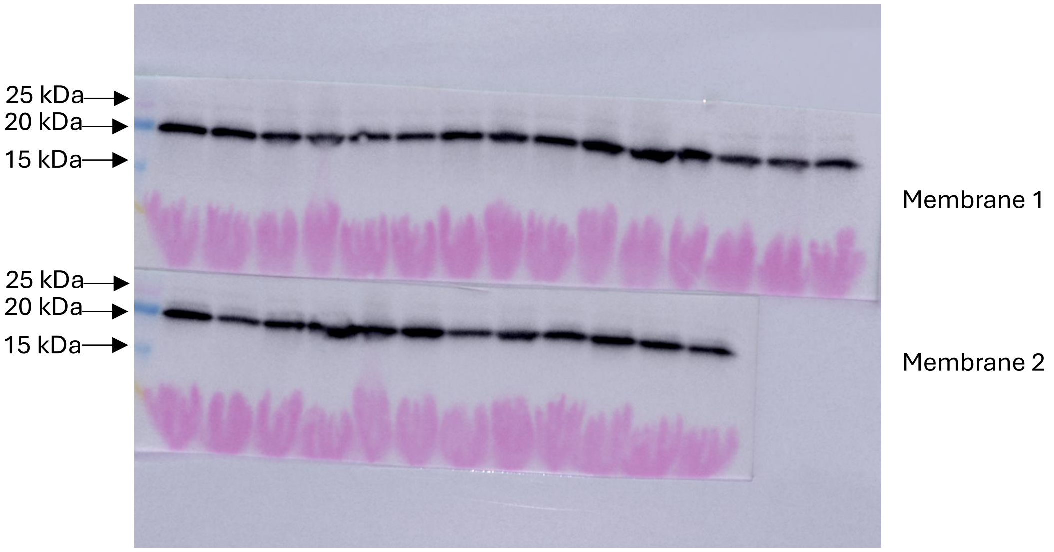

Figure 5A: Female ß-actin

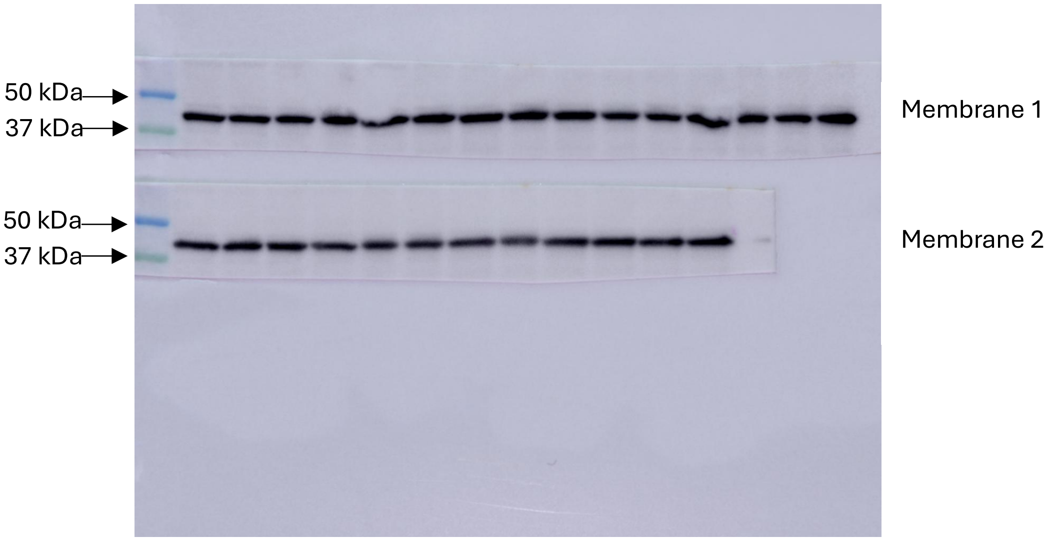

Figure 5A: Male FTH1

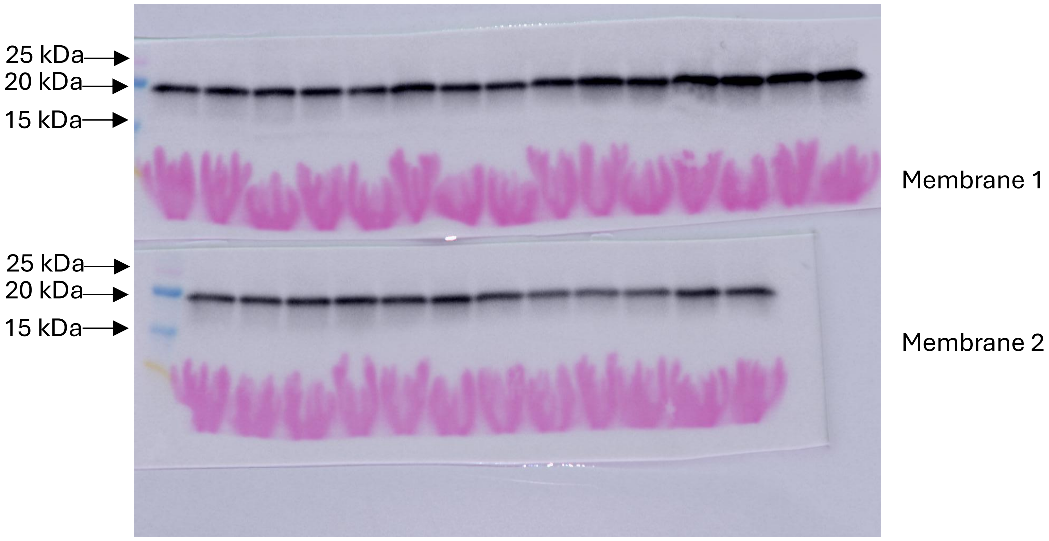

Figure 5A: Male FTL

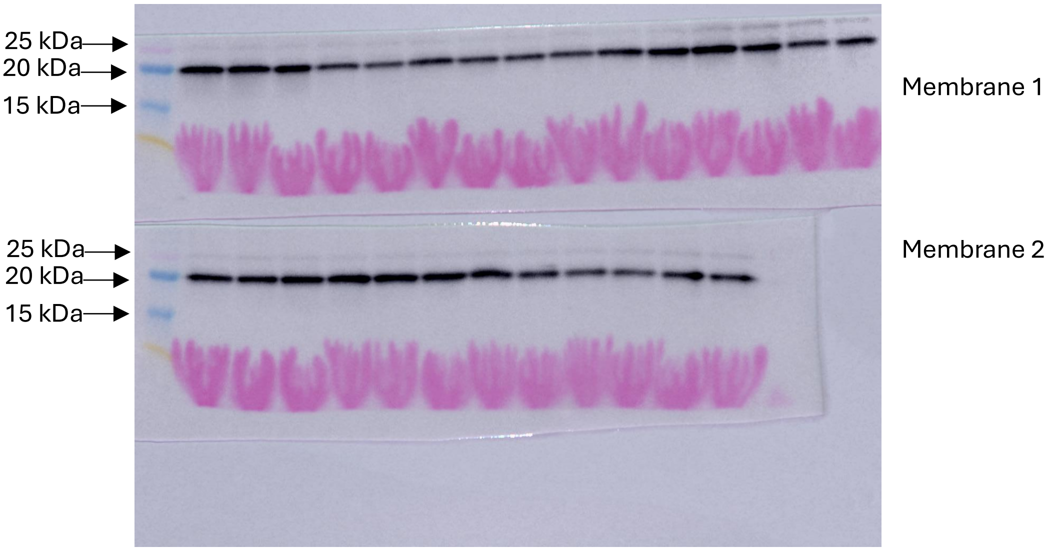

Figure 5A: Male β-actin

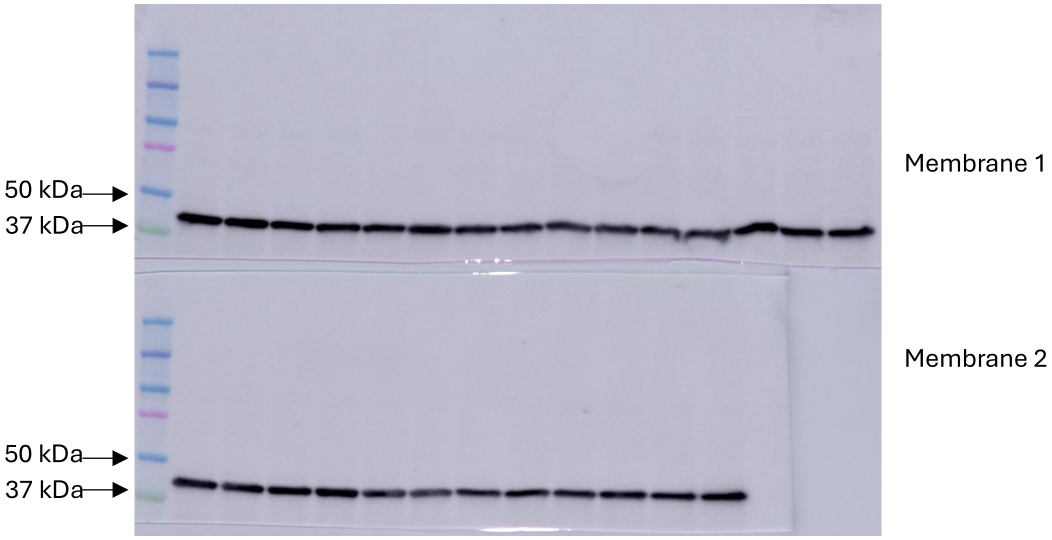

Figure 6: Lipocalin-2 (LCN2), Lipocalin-Receptor (LCN-R), & Glial Fibrillary Acidic Protein (GFAP) Images

Figure 6A: Female LCN2

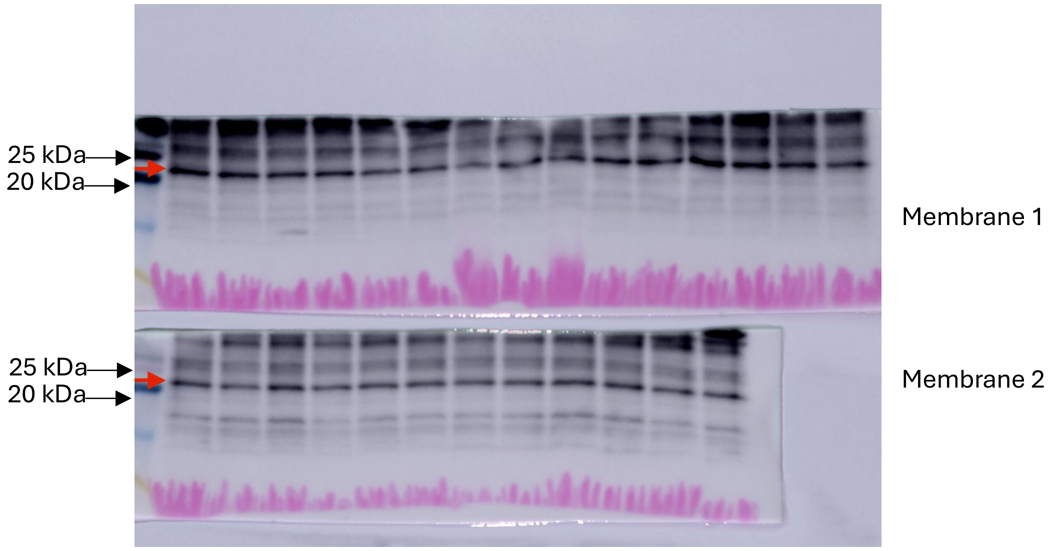

Figure 6A: Female LCN-R

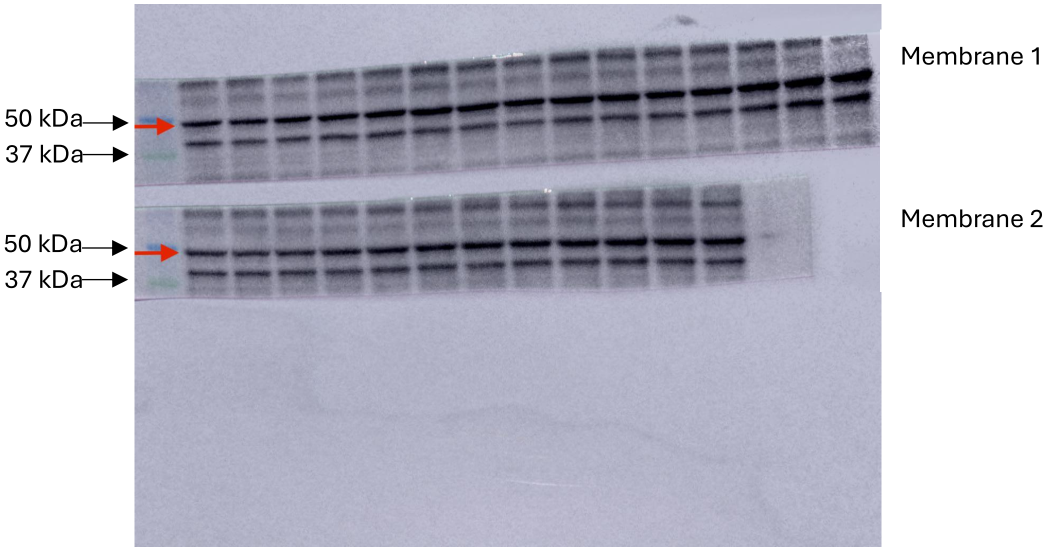

Figure 6A: Female GFAP

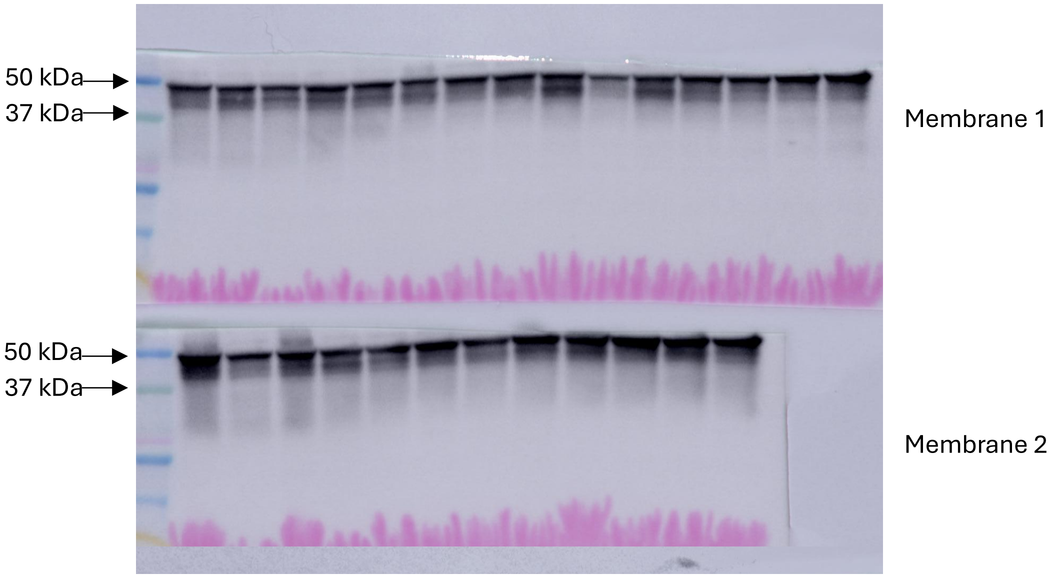

Figure 6A: Female ß-actin

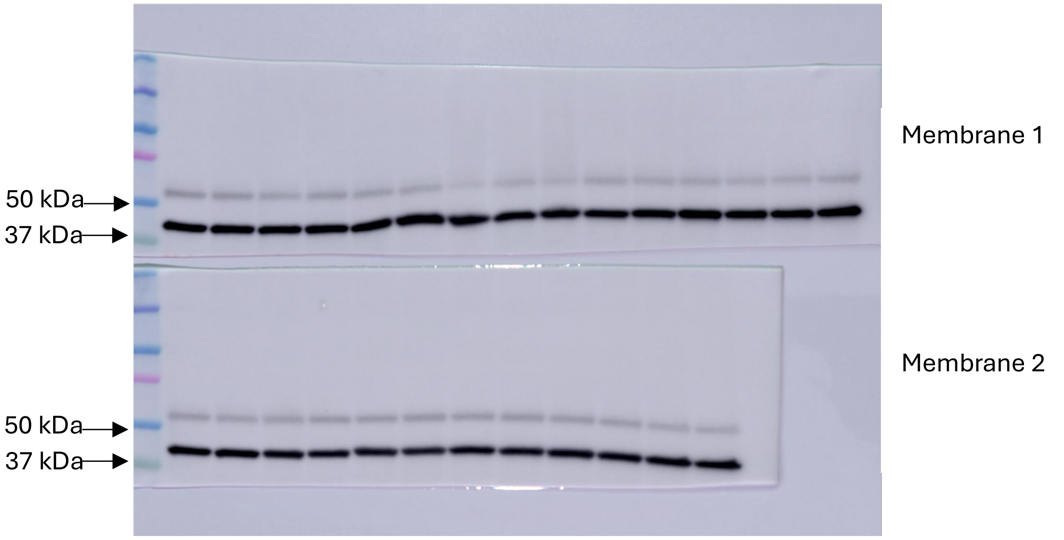

Figure 6A: Male LCN2

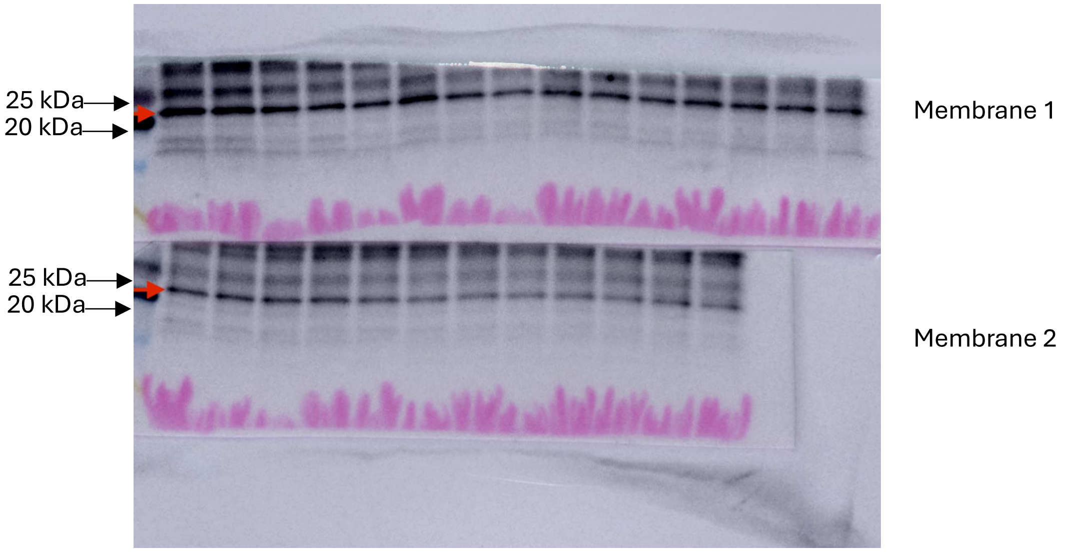

Figure 6A: Male LCN-R

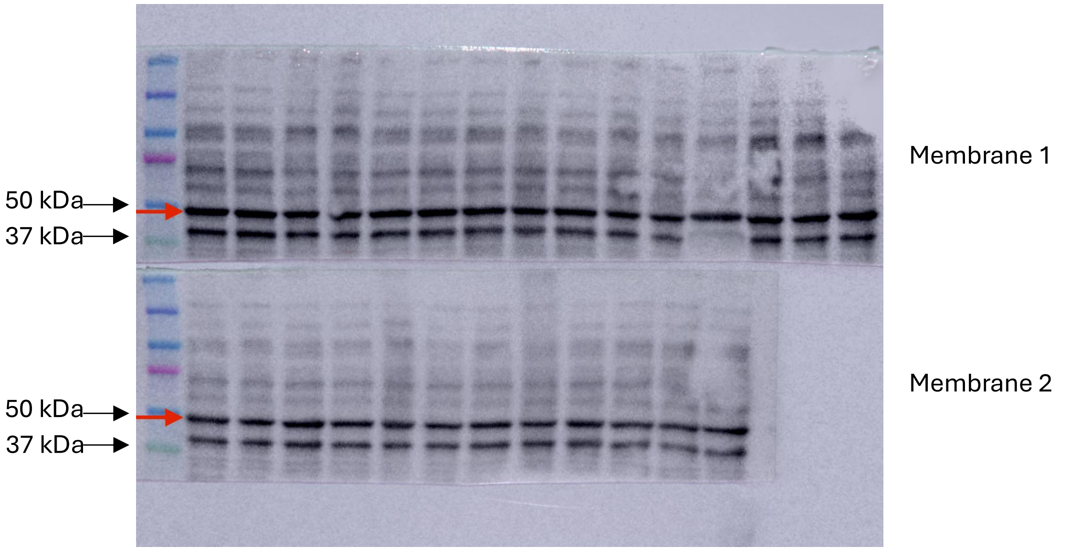

Figure 6A: Male GFAP

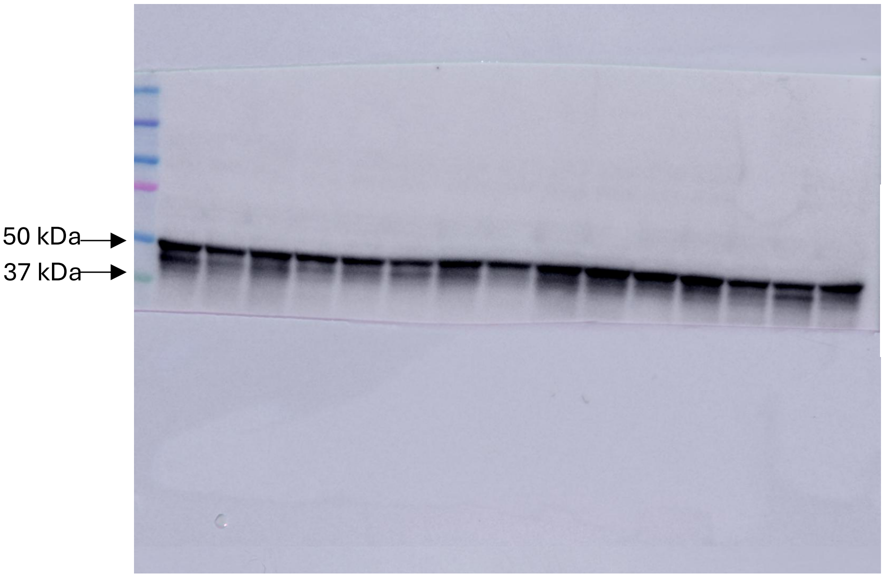

Figure 6A: Male GFAP

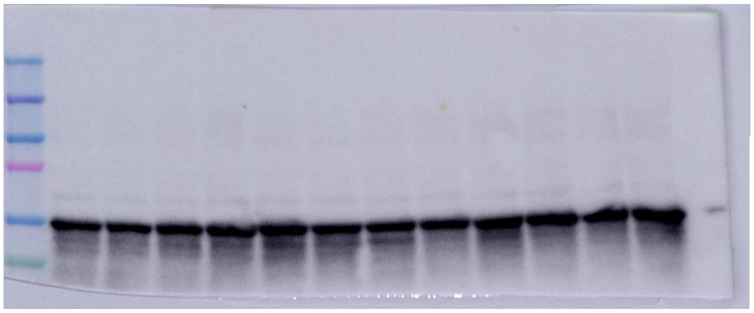

Figure 6A: Male ß-actin

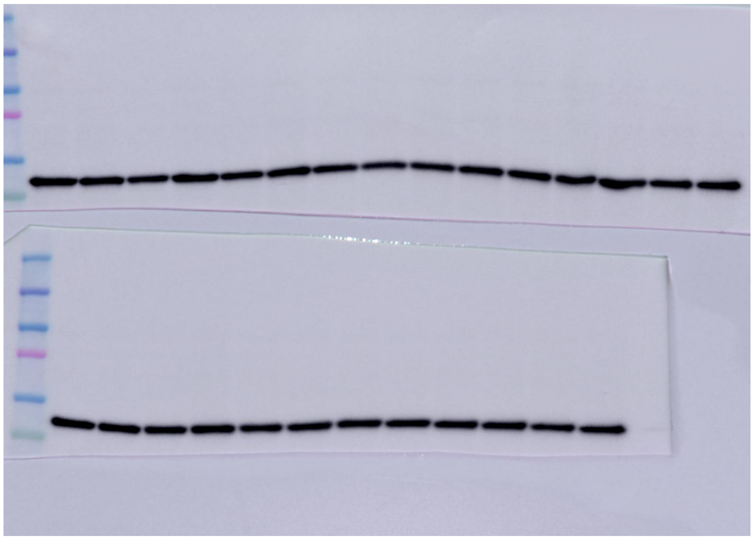

Figure 7: Catalase (CAT) & Superoxide Dismutase 2 (SOD2)

Figure 7A: Female CAT

75 kDa
50 kDa
37 kDa

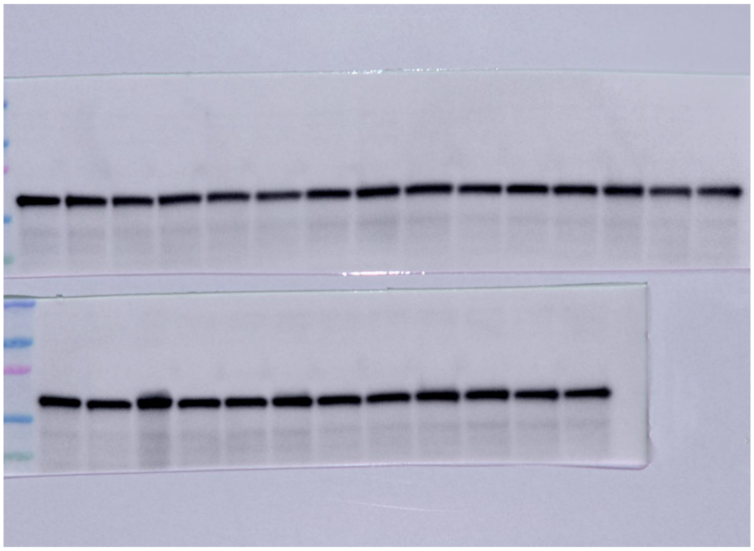

Membrane 1

75 kDa
50 kDa
37 kDa

Membrane 2

Figure 7A: Female SOD2

25 kDa
20 kDa
15 kDa

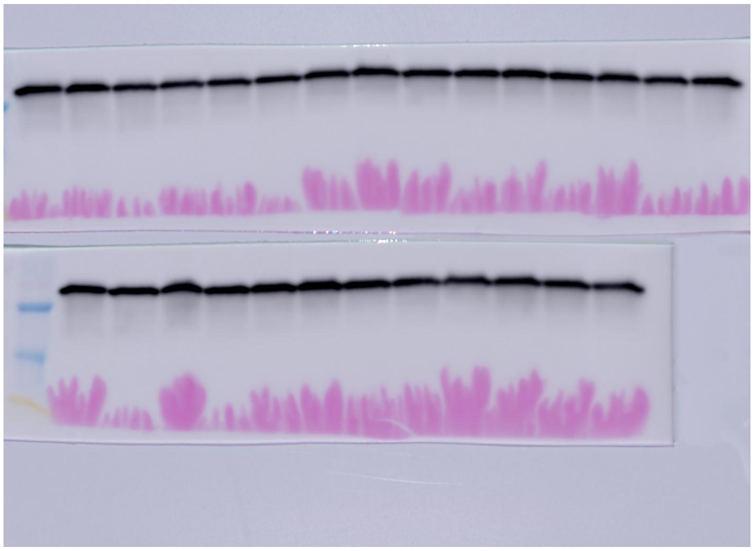

Membrane 1

25 kDa
20 kDa
15 kDa

Membrane 2

Figure 7A: Female ß-actin

50 kDa
37 kDa

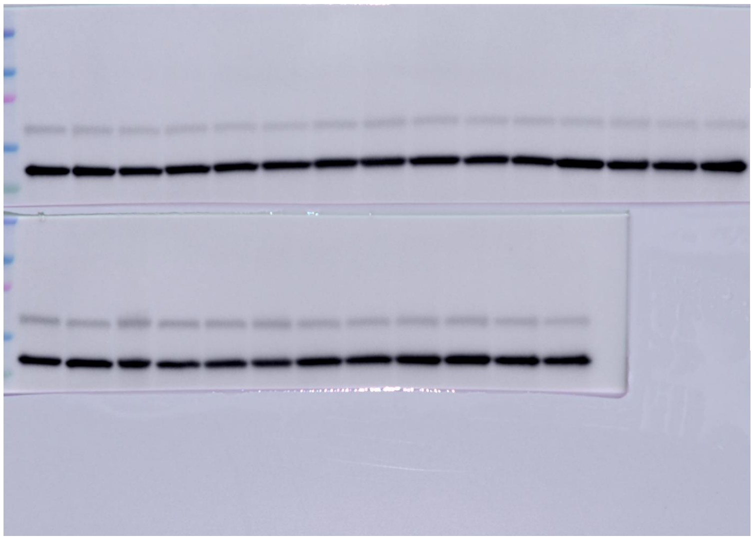

Membrane 1

50 kDa
37 kDa

Membrane 2

Figure 7A: Male CAT

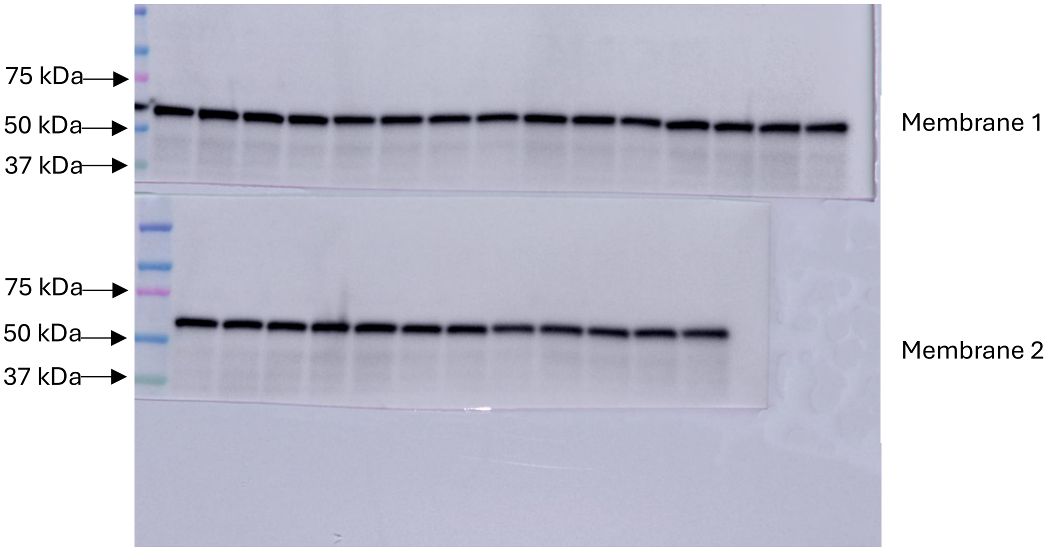

Figure 7A: Male SOD2

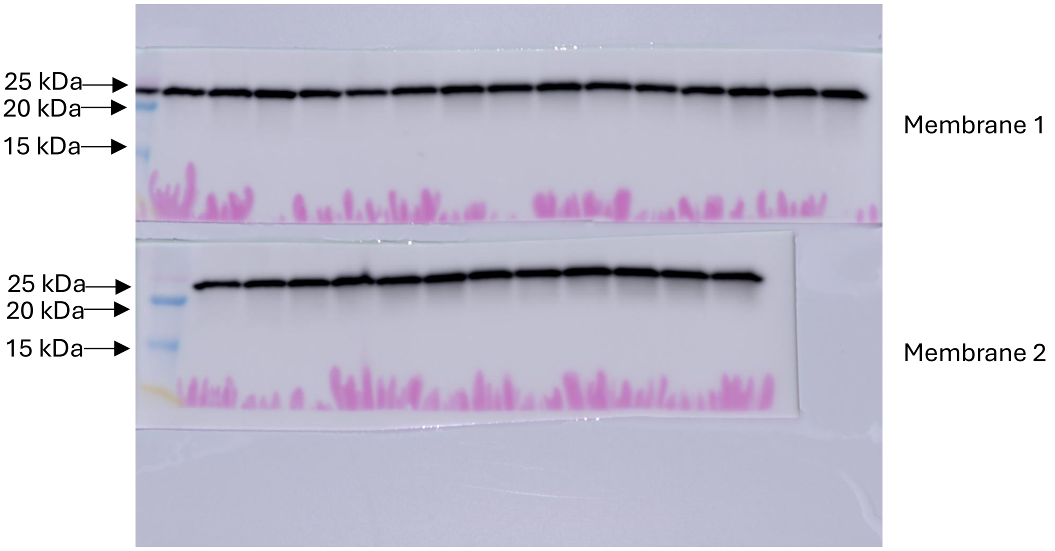

Figure 7A: Male ß-actin

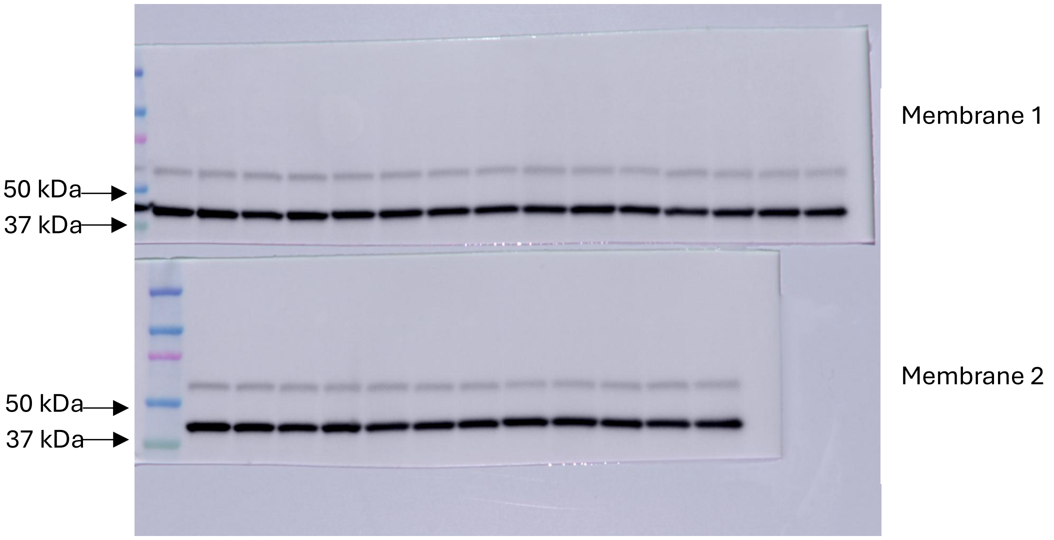

Raw Statistical Data: For all images (Figures 2-7)

Figure 2A

Table Analyzed	**Figure 2A** Hb Pre-treatment							
Two-way ANOVA	Ordinary							
Alpha	0.05							
Source of Variation	% of total variation	P value	P value summary	Significant?				
Interaction	1.25	0.0016	**	Yes				
Sex	0.8257	0.0099	**	Yes				
Diet	84.45	<0.0001	****	Yes				
ANOVA table	SS (Type III)	DF	MS	F (DFn, DFd)	P value			
Interaction	21.6	1	21.6	F (1, 104) = 10.44	P=0.0016			
Sex	14.26	1	14.26	F (1, 104) = 6.896	P=0.0099			
Diet	1459	1	1459	F (1, 104) = 705.3	P<0.0001			
Residual	215.1	104	2.068					
Difference between column means								
Predicted (LS) mean of Iron Adequate	14.98							
Predicted (LS) mean of Iron Deficient	7.188							
Difference between predicted means	7.796							
SE of difference	0.2935							
95% CI of difference	7.214 to 8.378							
Difference between row means								
Predicted (LS) mean of Males	10.7							
Predicted (LS) mean of Females	11.47							
Difference between predicted means	-0.7708							
SE of difference	0.2935							
95% CI of difference	-1.353 to -0.1887							
Interaction CI								
Mean diff, A1 - B1	8.744							
Mean diff, A2 - B2	6.847							
(A1 -B1) - (A2 - B2)	1.897							
95% CI of difference	0.7330 to 3.061							
(B1 - A1) - (B2 - A2)	-1.897							
95% CI of difference	-3.061 to -0.7330							
Data summary								
Number of columns (Diet)	2							
Number of rows (Sex)	2							
Number of values	108							
Number of families	1							
Number of comparisons per family	6							
Alpha	0.05							
Uncorrected Fisher's LSD	Predicted (LS) mea	95.00% CI of diff.	Below threshold?	Summary	Individual P Value			
Males:Iron Adequate vs. Males:Iron Deficient	8.744	7.921 to 9.568	Yes	****	<0.0001			
Males:Iron Adequate vs. Females:Iron Adequate	0.1778	-0.7728 to 1.128	No	ns	0.7115			
Males:Iron Adequate vs. Females:Iron Deficient	7.025	6.202 to 7.848	Yes	****	<0.0001			
Males:Iron Deficient vs. Females:Iron Adequate	-8.567	-9.390 to -7.743	Yes	****	<0.0001			
Males:Iron Deficient vs. Females:Iron Deficient	-1.719	-2.392 to -1.047	Yes	****	<0.0001			
Females:Iron Adequate vs. Females:Iron Deficient	6.847	6.024 to 7.670	Yes	****	<0.0001			
Test details	Predicted (LS) mea	Predicted (LS) mea	Predicted (LS) mea	SE of diff.	N1	N2	t	DF
Males:Iron Adequate vs. Males:Iron Deficient	15.07	6.328	8.744	0.4151	18	36	21.06	104
Males:Iron Adequate vs. Females:Iron Adequate	15.07	14.89	0.1778	0.4793	18	18	0.3709	104
Males:Iron Adequate vs. Females:Iron Deficient	15.07	8.047	7.025	0.4151	18	36	16.92	104
Males:Iron Deficient vs. Females:Iron Adequate	6.328	14.89	-8.567	0.4151	36	18	20.64	104
Males:Iron Deficient vs. Females:Iron Deficient	6.328	8.047	-1.719	0.3389	36	36	5.073	104
Females:Iron Adequate vs. Females:Iron Deficient	14.89	8.047	6.847	0.4151	18	36	16.49	104

Figure 2B

Table Analyzed	**Figure 2B** HCT Pre-treatment							
Two-way ANOVA	Ordinary							
Alpha	0.05							
Source of Variation	% of total variation	P value	P value summary	Significant?				
Interaction	1.076	0.0048	**	Yes				
Sex	0.9882	0.0068	**	Yes				
Diet	83.45	<0.0001	****	Yes				
ANOVA table	SS (Type III)	DF	MS	F (DFn, DFd)	P value			
Interaction	137.9	1	137.9	F (1, 104) = 8.321	P=0.0048			
Sex	126.7	1	126.7	F (1, 104) = 7.641	P=0.0068			
Diet	10696	1	10696	F (1, 104) = 645.3	P<0.0001			
Residual	1724	104	16.58					
Difference between column means								
Predicted (LS) mean of Iron Adequate	42.54							
Predicted (LS) mean of Iron Deficient	21.43							
Difference between predicted means	21.11							
SE of difference	0.831							
95% CI of difference	19.46 to 22.76							
Difference between row means								
Predicted (LS) mean of Males	30.84							
Predicted (LS) mean of Females	33.14							
Difference between predicted means	-2.297							
SE of difference	0.831							
95% CI of difference	-3.945 to -0.6492							
Interaction CI								
Mean diff, A1 - B1	23.51							
Mean diff, A2 - B2	18.71							
(A1 -B1) - (A2 - B2)	4.794							
95% CI of difference	1.498 to 8.090							
(B1 - A1) - (B2 - A2)	-4.794							
95% CI of difference	-8.090 to -1.498							
Data summary								
Number of columns (Diet)	2							
Number of rows (Sex)	2							
Number of values	108							
Number of families	1							
Number of comparisons per family	6							
Alpha	0.05							
Tukey's multiple comparisons test	Predicted (LS) mean	95.00% CI of diff.	Below threshold?	Summary	Adjusted P Value			
Males:Iron Adequate vs. Males:Iron Deficient	23.51	20.44 to 26.58	Yes	****	<0.0001			
Males:Iron Adequate vs. Females:Iron Adequate	0.1	-3.443 to 3.643	No	ns	0.9999			
Males:Iron Adequate vs. Females:Iron Deficient	18.81	15.75 to 21.88	Yes	****	<0.0001			
Males:Iron Deficient vs. Females:Iron Adequate	-23.41	-26.48 to -20.34	Yes	****	<0.0001			
Males:Iron Deficient vs. Females:Iron Deficient	-4.694	-7.200 to -2.189	Yes	****	<0.0001			
Females:Iron Adequate vs. Females:Iron Deficient	18.71	15.65 to 21.78	Yes	****	<0.0001			
Test details	Predicted (LS) mean	Predicted (LS) mea	Predicted (LS) mea	SE of diff.	N1	N2	q	DF
Males:Iron Adequate vs. Males:Iron Deficient	42.59	19.09	23.51	1.175	18	36	28.29	104
Males:Iron Adequate vs. Females:Iron Adequate	42.59	42.49	0.1	1.357	18	18	0.1042	104
Males:Iron Adequate vs. Females:Iron Deficient	42.59	23.78	18.81	1.175	18	36	22.64	104
Males:Iron Deficient vs. Females:Iron Adequate	19.09	42.49	-23.41	1.175	36	18	28.17	104
Males:Iron Deficient vs. Females:Iron Deficient	19.09	23.78	-4.694	0.9596	36	36	6.918	104
Females:Iron Adequate vs. Females:Iron Deficient	42.49	23.78	18.71	1.175	18	36	22.52	104

Figure 2C

Table Analyzed	**Figure 2C** Hb Post-treatment				
Two-way ANOVA	Ordinary				
Alpha	0.05				
Source of Variation	% of total variation	P value	P value summary	Significant?	
Interaction	0.8418	0.0111	*	Yes	
Sex	0.3147	0.0637	ns	No	
Diet	89.71	<0.0001	****	Yes	
ANOVA table	SS	DF	MS	F (DFn, DFd)	P value
Interaction	11.72	2	5.859	F (2, 102) = 4.701	P=0.0111
Sex	4.38	1	4.38	F (1, 102) = 3.515	P=0.0637
Diet	1249	2	624.4	F (2, 102) = 501.0	P<0.0001
Residual	127.1	102	1.246		
Difference between row means					
Mean of Males	12.04				
Mean of Females	12.44				
Difference between means	-0.4028				
SE of difference	0.2148				
95% CI of difference	-0.8289 to 0.02336				
Data summary					
Number of columns (Diet)	3				
Number of rows (Sex)	2				
Number of values	108				
Number of families	1				
Number of comparisons per family	15				
Alpha	0.05				

Tukey's multiple comparisons test	Mean Diff.	95.00% CI of di	Below threshold?	Summary	Adjusted P Value
Males:Iron Adequate vs. Males:Iron Deficient	8.658	7.577 to 9.739	Yes	****	<0.0001
Males:Iron Adequate vs. Males:Iron Repletion	1.817	0.7358 to 2.898	Yes	****	<0.0001
Males:Iron Adequate vs. Females:Iron Adequate	0.2833	-0.7975 to 1.36	No	ns	0.9733
Males:Iron Adequate vs. Females:Iron Deficient	7.367	6.286 to 8.448	Yes	****	<0.0001
Males:Iron Adequate vs. Females:Iron Repletion	1.617	0.5358 to 2.698	Yes	***	0.0005
Males:Iron Deficient vs. Males:Iron Repletion	-6.842	-7.923 to -5.761	Yes	****	<0.0001
Males:Iron Deficient vs. Females:Iron Adequate	-8.375	-9.456 to -7.294	Yes	****	<0.0001
Males:Iron Deficient vs. Females:Iron Deficient	-1.292	-2.373 to -0.210	Yes	**	0.0097
Males:Iron Deficient vs. Females:Iron Repletion	-7.042	-8.123 to -5.961	Yes	****	<0.0001
Males:Iron Repletion vs. Females:Iron Adequate	-1.533	-2.614 to -0.452	Yes	**	0.0011
Males:Iron Repletion vs. Females:Iron Deficient	5.55	4.469 to 6.631	Yes	****	<0.0001
Males:Iron Repletion vs. Females:Iron Repletion	-0.2	-1.281 to 0.880	No	ns	0.9945
Females:Iron Adequate vs. Females:Iron Deficient	7.083	6.002 to 8.164	Yes	****	<0.0001
Females:Iron Adequate vs. Females:Iron Repletion	1.333	0.2525 to 2.414	Yes	**	0.0067
Females:Iron Deficient vs. Females:Iron Repletion	-5.75	-6.831 to -4.669	Yes	****	<0.0001

Test details	Mean 1	Mean 2	Mean Diff.	SE of diff.	N1	N2	q	DF
Males:Iron Adequate vs. Males:Iron Deficient	15.53	6.869	8.658	0.3721	18	18	32.91	102
Males:Iron Adequate vs. Males:Iron Repletion	15.53	13.71	1.817	0.3721	18	18	6.904	102
Males:Iron Adequate vs. Females:Iron Adequate	15.53	15.24	0.2833	0.3721	18	18	1.077	102
Males:Iron Adequate vs. Females:Iron Deficient	15.53	8.161	7.367	0.3721	18	18	28	102
Males:Iron Adequate vs. Females:Iron Repletion	15.53	13.91	1.617	0.3721	18	18	6.144	102
Males:Iron Deficient vs. Males:Iron Repletion	6.869	13.71	-6.842	0.3721	18	18	26	102
Males:Iron Deficient vs. Females:Iron Adequate	6.869	15.24	-8.375	0.3721	18	18	31.83	102
Males:Iron Deficient vs. Females:Iron Deficient	6.869	8.161	-1.292	0.3721	18	18	4.909	102
Males:Iron Deficient vs. Females:Iron Repletion	6.869	13.91	-7.042	0.3721	18	18	26.76	102
Males:Iron Repletion vs. Females:Iron Adequate	13.71	15.24	-1.533	0.3721	18	18	5.827	102
Males:Iron Repletion vs. Females:Iron Deficient	13.71	8.161	5.55	0.3721	18	18	21.09	102
Males:Iron Repletion vs. Females:Iron Repletion	13.71	13.91	-0.2	0.3721	18	18	0.7601	102
Females:Iron Adequate vs. Females:Iron Deficient	15.24	8.161	7.083	0.3721	18	18	26.92	102
Females:Iron Adequate vs. Females:Iron Repletion	15.24	13.91	1.333	0.3721	18	18	5.067	102
Females:Iron Deficient vs. Females:Iron Repletion	8.161	13.91	-5.75	0.3721	18	18	21.85	102

Figure 2D

Table Analyzed	**Figure 2D** HCT Post-treatment

Two-way ANOVA	Ordinary
Alpha	0.05

Source of Variation	% of total variation	P value	P value summary	Significant?
Interaction	0.6684	0.0237	*	Yes
Sex	0.1752	0.1567	ns	No
Diet	90.38	<0.0001	****	Yes

ANOVA table	SS	DF	MS	F (DFn, DFd)	P value
Interaction	70.9	2	35.45	F (2, 102) = 3.882	P=0.0237
Sex	18.58	1	18.58	F (1, 102) = 2.035	P=0.1567
Diet	9586	2	4793	F (2, 102) = 525.0	P<0.0001
Residual	931.3	102	9.131		

Difference between row means	
Mean of Males	35.25
Mean of Females	36.08
Difference between means	-0.8296
SE of difference	0.5815
95% CI of difference	-1.983 to 0.3238

Data summary	
Number of columns (Diet)	3
Number of rows (Sex)	2
Number of values	108

Number of families	1
Number of comparisons per family	15
Alpha	0.05

Tukey's multiple comparisons test	Mean Diff.	95.00% CI of diff.	Below threshold?	Summary	Adjusted P Value
Males:Iron Adequate vs. Males:Iron Deficient	22.84	19.91 to 25.76	Yes	****	<0.0001
Males:Iron Adequate vs. Males:Iron Repletion	2.489	-0.4367 to 5.414	No	ns	0.1426
Males:Iron Adequate vs. Females:Iron Adequate	0.6056	-2.320 to 3.531	No	ns	0.9907
Males:Iron Adequate vs. Females:Iron Deficient	19.74	16.82 to 22.67	Yes	****	<0.0001
Males:Iron Adequate vs. Females:Iron Repletion	2.489	-0.4367 to 5.414	No	ns	0.1426
Males:Iron Deficient vs. Males:Iron Repletion	-20.35	-23.28 to -17.42	Yes	****	<0.0001
Males:Iron Deficient vs. Females:Iron Adequate	-22.23	-25.16 to -19.31	Yes	****	<0.0001
Males:Iron Deficient vs. Females:Iron Deficient	-3.094	-6.020 to -0.1688	Yes	*	0.0317
Males:Iron Deficient vs. Females:Iron Repletion	-20.35	-23.28 to -17.42	Yes	****	<0.0001
Males:Iron Repletion vs. Females:Iron Adequate	-1.883	-4.809 to 1.042	No	ns	0.4266
Males:Iron Repletion vs. Females:Iron Deficient	17.26	14.33 to 20.18	Yes	****	<0.0001
Males:Iron Repletion vs. Females:Iron Repletion	0	-2.926 to 2.926	No	ns	>0.9999
Females:Iron Adequate vs. Females:Iron Deficient	19.14	16.21 to 22.06	Yes	****	<0.0001
Females:Iron Adequate vs. Females:Iron Repletion	1.883	-1.042 to 4.809	No	ns	0.4266
Females:Iron Deficient vs. Females:Iron Repletion	-17.26	-20.18 to -14.33	Yes	****	<0.0001

Test details	Mean 1	Mean 2	Mean Diff.	SE of diff.	N1	N2	q	DF
Males:Iron Adequate vs. Males:Iron Deficient	43.69	20.86	22.84	1.007	18	18	32.07	102
Males:Iron Adequate vs. Males:Iron Repletion	43.69	41.21	2.489	1.007	18	18	3.495	102
Males:Iron Adequate vs. Females:Iron Adequate	43.69	43.09	0.6056	1.007	18	18	0.8502	102
Males:Iron Adequate vs. Females:Iron Deficient	43.69	23.95	19.74	1.007	18	18	27.72	102
Males:Iron Adequate vs. Females:Iron Repletion	43.69	41.21	2.489	1.007	18	18	3.495	102
Males:Iron Deficient vs. Males:Iron Repletion	20.86	41.21	-20.35	1.007	18	18	28.57	102
Males:Iron Deficient vs. Females:Iron Adequate	20.86	43.09	-22.23	1.007	18	18	31.22	102
Males:Iron Deficient vs. Females:Iron Deficient	20.86	23.95	-3.094	1.007	18	18	4.345	102
Males:Iron Deficient vs. Females:Iron Repletion	20.86	41.21	-20.35	1.007	18	18	28.57	102
Males:Iron Repletion vs. Females:Iron Adequate	41.21	43.09	-1.883	1.007	18	18	2.644	102
Males:Iron Repletion vs. Females:Iron Deficient	41.21	23.95	17.26	1.007	18	18	24.23	102
Males:Iron Repletion vs. Females:Iron Repletion	41.21	41.21	0	1.007	18	18	0	102
Females:Iron Adequate vs. Females:Iron Deficient	43.09	23.95	19.14	1.007	18	18	26.87	102
Females:Iron Adequate vs. Females:Iron Repletion	43.09	41.21	1.883	1.007	18	18	2.644	102
Females:Iron Deficient vs. Females:Iron Repletion	23.95	41.21	-17.26	1.007	18	18	24.23	102

Figure 3A

Table Analyzed	**Figure 3A** IA X drug							
Two-way ANOVA	Ordinary							
Alpha	0.05							
Source of Variation	% of total variation	P value	P value sun	Significant?				
Interaction	3.259	0.504	ns	No				
Sex	26.55	0.002	**	Yes				
Drug	0.454	0.9073	ns	No				
ANOVA table	SS	DF	MS	F (DFn, DFd)	P value			
Interaction	662.1	2	331.1	F (2, 30) = 0.7011	P=0.5040			
Sex	5394	1	5394	F (1, 30) = 11.42	P=0.0020			
Drug	92.22	2	46.11	F (2, 30) = 0.09765	P=0.9073			
Residual	14165	30	472.2					
Difference between row means								
Mean of Males	147.8							
Mean of Females	123.3							
Difference between means	24.48							
SE of difference	7.243							
95% CI of difference	9.689 to 39.27							
Data summary								
Number of columns (Drug)	3							
Number of rows (Sex)	2							
Number of values	36							
Number of families	1							
Number of comparisons per family	15							
Alpha	0.05							
Tukey's multiple comparisons test	Mean Diff.	95.00% CI of diff.	Below thres	Summary	Adjusted P Value			
Males:Vehicle vs. Males:Carbidopa + L-DOPA	-4.433	-42.59 to 33.73	No	ns	0.9992			
Males:Vehicle vs. Males:Selegiline	2.82	-35.34 to 40.98	No	ns	>0.9999			
Males:Vehicle vs. Females:Vehicle	26.09	-12.07 to 64.25	No	ns	0.3246			
Males:Vehicle vs. Females:Carbidopa + L-DOPA	29.66	-8.500 to 67.82	No	ns	0.2012			
Males:Vehicle vs. Females:Selegiline	16.09	-22.07 to 54.25	No	ns	0.7921			
Males:Carbidopa + L-DOPA vs. Males:Selegiline	7.253	-30.91 to 45.41	No	ns	0.9918			
Males:Carbidopa + L-DOPA vs. Females:Vehicle	30.52	-7.639 to 68.68	No	ns	0.1773			
Males:Carbidopa + L-DOPA vs. Females:Carbidopa + L-DOI	34.09	-4.067 to 72.25	No	ns	0.1012			
Males:Carbidopa + L-DOPA vs. Females:Selegiline	20.52	-17.64 to 58.68	No	ns	0.5826			
Males:Selegiline vs. Females:Vehicle	23.27	-14.89 to 61.43	No	ns	0.4482			
Males:Selegiline vs. Females:Carbidopa + L-DOPA	26.84	-11.32 to 65.00	No	ns	0.2953			
Males:Selegiline vs. Females:Selegiline	13.27	-24.89 to 51.43	No	ns	0.8941			
Females:Vehicle vs. Females:Carbidopa + L-DOPA	3.572	-34.59 to 41.73	No	ns	0.9997			
Females:Vehicle vs. Females:Selegiline	-10	-48.16 to 28.16	No	ns	0.9658			
Females:Carbidopa + L-DOPA vs. Females:Selegiline	-13.57	-51.73 to 24.59	No	ns	0.8848			
Test details	Mean 1	Mean 2	Mean Diff.	SE of diff.	N1	N2	q	DF
Males:Vehicle vs. Males:Carbidopa + L-DOPA	147.3	151.7	-4.433	12.55	6	6	0.4997	30
Males:Vehicle vs. Males:Selegiline	147.3	144.5	2.82	12.55	6	6	0.3179	30
Males:Vehicle vs. Females:Vehicle	147.3	121.2	26.09	12.55	6	6	2.941	30
Males:Vehicle vs. Females:Carbidopa + L-DOPA	147.3	117.6	29.66	12.55	6	6	3.343	30
Males:Vehicle vs. Females:Selegiline	147.3	131.2	16.09	12.55	6	6	1.813	30
Males:Carbidopa + L-DOPA vs. Males:Selegiline	151.7	144.5	7.253	12.55	6	6	0.8176	30
Males:Carbidopa + L-DOPA vs. Females:Vehicle	151.7	121.2	30.52	12.55	6	6	3.44	30
Males:Carbidopa + L-DOPA vs. Females:Carbidopa + L-DOI	151.7	117.6	34.09	12.55	6	6	3.843	30
Males:Carbidopa + L-DOPA vs. Females:Selegiline	151.7	131.2	20.52	12.55	6	6	2.313	30
Males:Selegiline vs. Females:Vehicle	144.5	121.2	23.27	12.55	6	6	2.623	30
Males:Selegiline vs. Females:Carbidopa + L-DOPA	144.5	117.6	26.84	12.55	6	6	3.025	30
Males:Selegiline vs. Females:Selegiline	144.5	131.2	13.27	12.55	6	6	1.495	30
Females:Vehicle vs. Females:Carbidopa + L-DOPA	121.2	117.6	3.572	12.55	6	6	0.4026	30
Females:Vehicle vs. Females:Selegiline	121.2	131.2	-10	12.55	6	6	1.127	30
Females:Carbidopa + L-DOPA vs. Females:Selegiline	117.6	131.2	-13.57	12.55	6	6	1.53	30

Figure 3B

Table Analyzed	**Figure 3B** ID X drug								
Two-way ANOVA	Ordinary								
Alpha	0.05								
Source of Variation	% of total variation	P value	P value summary	Significant?					
Interaction	0.1264	0.9784	ns	No					
Sex	12.71	0.0445	*	Yes					
Drug	0.461	0.9235	ns	No					
ANOVA table	SS	DF	MS	F (DFn, DFd)	P value				
Interaction	13.16	2	6.582	F (2, 30) = 0.02188	P=0.9784				
Sex	1323	1	1323	F (1, 30) = 4.399	P=0.0445				
Drug	47.99	2	23.99	F (2, 30) = 0.07976	P=0.9235				
Residual	9025	30	300.8						
Difference between row means									
Mean of Males	100.7								
Mean of Females	88.56								
Difference between means	12.13								
SE of difference	5.782								
95% CI of difference	0.3185 to 23.93								
Data summary									
Number of columns (Drug)	3								
Number of rows (Sex)	2								
Number of values	36								
Number of families	1								
Number of comparisons per family	15								
Alpha	0.05								
Tukey's multiple comparisons test	Mean Diff.	95.00% CI of diff.	Below threshold?	Summary	Adjusted P Value				
Males:Vehicle vs. Males:Carbidopa + L-DOP/	-2.712	-33.17 to 27.75	No	ns	0.9998				
Males:Vehicle vs. Males:Selegiline	-2.165	-32.62 to 28.29	No	ns	>0.9999				
Males:Vehicle vs. Females:Vehicle	11.96	-18.50 to 42.42	No	ns	0.836				
Males:Vehicle vs. Females:Carbidopa + L-DC	10.97	-19.49 to 41.43	No	ns	0.8793				
Males:Vehicle vs. Females:Selegiline	8.57	-21.89 to 39.03	No	ns	0.954				
Males:Carbidopa + L-DOPA vs. Males:Selegil	0.5467	-29.91 to 31.01	No	ns	>0.9999				
Males:Carbidopa + L-DOPA vs. Females:Veh	14.67	-15.79 to 45.13	No	ns	0.6881				
Males:Carbidopa + L-DOPA vs. Females:Carl	13.68	-16.78 to 44.14	No	ns	0.746				
Males:Carbidopa + L-DOPA vs. Females:Sele	11.28	-19.18 to 41.74	No	ns	0.8664				
Males:Selegiline vs. Females:Vehicle	14.13	-16.33 to 44.58	No	ns	0.7205				
Males:Selegiline vs. Females:Carbidopa + L-	13.14	-17.32 to 43.60	No	ns	0.7763				
Males:Selegiline vs. Females:Selegiline	10.74	-19.72 to 41.19	No	ns	0.8886				
Females:Vehicle vs. Females:Carbidopa + L-	-0.9883	-31.45 to 29.47	No	ns	>0.9999				
Females:Vehicle vs. Females:Selegiline	-3.39	-33.85 to 27.07	No	ns	0.9993				
Females:Carbidopa + L-DOPA vs. Females:S	-2.402	-32.86 to 28.06	No	ns	0.9999				
Test details	Mean 1	Mean 2	Mean Diff.	SE of diff.	N1	N2	q	DF	
Males:Vehicle vs. Males:Carbidopa + L-DOP/	99.06	101.8	-2.712	10.01	6	6	0.3829	30	
Males:Vehicle vs. Males:Selegiline	99.06	101.2	-2.165	10.01	6	6	0.3057	30	
Males:Vehicle vs. Females:Vehicle	99.06	87.1	11.96	10.01	6	6	1.689	30	
Males:Vehicle vs. Females:Carbidopa + L-DC	99.06	88.09	10.97	10.01	6	6	1.549	30	
Males:Vehicle vs. Females:Selegiline	99.06	90.49	8.57	10.01	6	6	1.21	30	
Males:Carbidopa + L-DOPA vs. Males:Selegil	101.8	101.2	0.5467	10.01	6	6	0.0772	30	
Males:Carbidopa + L-DOPA vs. Females:Veh	101.8	87.1	14.67	10.01	6	6	2.072	30	
Males:Carbidopa + L-DOPA vs. Females:Carl	101.8	88.09	13.68	10.01	6	6	1.932	30	
Males:Carbidopa + L-DOPA vs. Females:Sele	101.8	90.49	11.28	10.01	6	6	1.593	30	
Males:Selegiline vs. Females:Vehicle	101.2	87.1	14.13	10.01	6	6	1.995	30	
Males:Selegiline vs. Females:Carbidopa + L-	101.2	88.09	13.14	10.01	6	6	1.855	30	
Males:Selegiline vs. Females:Selegiline	101.2	90.49	10.74	10.01	6	6	1.516	30	
Females:Vehicle vs. Females:Carbidopa + L-	87.1	88.09	-0.9883	10.01	6	6	0.1396	30	
Females:Vehicle vs. Females:Selegiline	87.1	90.49	-3.39	10.01	6	6	0.4787	30	
Females:Carbidopa + L-DOPA vs. Females:S	88.09	90.49	-2.402	10.01	6	6	0.3392	30	

Figure 3C

Table Analyzed	**Figure 3C** IR X drug							
Two-way ANOVA	Ordinary							
Alpha	0.05							
Source of Variation	% of total variatio	P value	P value summary	Significant?				
Interaction	23.73	<0.0001	****	Yes				
Sex	18.85	<0.0001	****	Yes				
Drug	32.77	<0.0001	****	Yes				
ANOVA table	SS	DF	MS	F (DFn, DFd)	P value			
Interaction	13352	2	6676	F (2, 30) = 14.44	P<0.0001			
Sex	10608	1	10608	F (1, 30) = 22.95	P<0.0001			
Drug	18438	2	9219	F (2, 30) = 19.94	P<0.0001			
Residual	13868	30	462.3					
Difference between row means								
Mean of Males	134.8							
Mean of Females	100.5							
Difference between means	34.33							
SE of difference	7.167							
95% CI of difference	19.69 to 48.97							
Data summary								
Number of columns (Drug)	3							
Number of rows (Sex)	2							
Number of values	36							
Number of families	1							
Number of comparisons per family	15							
Alpha	0.05							
Tukey's multiple comparisons test	Mean Diff.	95.00% CI of diff.	Below threshold?	Summary	Adjusted P Value			
Males:Vehicle vs. Males:Carbidopa + L-DOPA	-93.74	-131.5 to -55.99	Yes	****	<0.0001			
Males:Vehicle vs. Males:Selegiline	-10.86	-48.62 to 26.90	No	ns	0.9496			
Males:Vehicle vs. Females:Vehicle	0.695	-37.06 to 38.45	No	ns	>0.9999			
Males:Vehicle vs. Females:Carbidopa + L-DOPA	-5.488	-43.24 to 32.27	No	ns	0.9976			
Males:Vehicle vs. Females:Selegiline	3.183	-34.57 to 40.94	No	ns	0.9998			
Males:Carbidopa + L-DOPA vs. Males:Selegiline	82.88	45.13 to 120.6	Yes	****	<0.0001			
Males:Carbidopa + L-DOPA vs. Females:Vehicle	94.44	56.68 to 132.2	Yes	****	<0.0001			
Males:Carbidopa + L-DOPA vs. Females:Carbido	88.26	50.50 to 126.0	Yes	****	<0.0001			
Males:Carbidopa + L-DOPA vs. Females:Selegilir	96.93	59.17 to 134.7	Yes	****	<0.0001			
Males:Selegiline vs. Females:Vehicle	11.56	-26.20 to 49.31	No	ns	0.9353			
Males:Selegiline vs. Females:Carbidopa + L-DOF	5.372	-32.38 to 43.13	No	ns	0.9979			
Males:Selegiline vs. Females:Selegiline	14.04	-23.71 to 51.80	No	ns	0.8644			
Females:Vehicle vs. Females:Carbidopa + L-DOF	-6.183	-43.94 to 31.57	No	ns	0.9959			
Females:Vehicle vs. Females:Selegiline	2.488	-35.27 to 40.24	No	ns	>0.9999			
Females:Carbidopa + L-DOPA vs. Females:Seleg	8.672	-29.08 to 46.43	No	ns	0.9807			
Test details	Mean 1	Mean 2	Mean Diff.	SE of diff.	N1	N2	q	DF
Males:Vehicle vs. Males:Carbidopa + L-DOPA	99.93	193.7	-93.74	12.41	6	6	10.68	30
Males:Vehicle vs. Males:Selegiline	99.93	110.8	-10.86	12.41	6	6	1.237	30
Males:Vehicle vs. Females:Vehicle	99.93	99.24	0.695	12.41	6	6	0.07918	30
Males:Vehicle vs. Females:Carbidopa + L-DOPA	99.93	105.4	-5.488	12.41	6	6	0.6253	30
Males:Vehicle vs. Females:Selegiline	99.93	96.75	3.183	12.41	6	6	0.3627	30
Males:Carbidopa + L-DOPA vs. Males:Selegiline	193.7	110.8	82.88	12.41	6	6	9.443	30
Males:Carbidopa + L-DOPA vs. Females:Vehicle	193.7	99.24	94.44	12.41	6	6	10.76	30
Males:Carbidopa + L-DOPA vs. Females:Carbido	193.7	105.4	88.26	12.41	6	6	10.05	30
Males:Carbidopa + L-DOPA vs. Females:Selegilir	193.7	96.75	96.93	12.41	6	6	11.04	30
Males:Selegiline vs. Females:Vehicle	110.8	99.24	11.56	12.41	6	6	1.316	30
Males:Selegiline vs. Females:Carbidopa + L-DOF	110.8	105.4	5.372	12.41	6	6	0.612	30
Males:Selegiline vs. Females:Selegiline	110.8	96.75	14.04	12.41	6	6	1.6	30
Females:Vehicle vs. Females:Carbidopa + L-DOF	99.24	105.4	-6.183	12.41	6	6	0.7045	30
Females:Vehicle vs. Females:Selegiline	99.24	96.75	2.488	12.41	6	6	0.2835	30
Females:Carbidopa + L-DOPA vs. Females:Seleg	105.4	96.75	8.672	12.41	6	6	0.988	30

Figure 3D

Table Analyzed	**Figure 3D** CtrlX diet				
Two-way ANOVA	Ordinary				
Alpha	0.05				
Source of Variation	% of total variation	P value	P value summary	Significant?	
Interaction	4.149	0.2214	ns	No	
Sex	6.412	0.0346	*	Yes	
Diet	50.21	<0.0001	****	Yes	
ANOVA table	SS	DF	MS	F (DFn, DFd)	P value
Interaction	971.2	2	485.6	F (2, 30) = 1.586	P=0.2214
Sex	1501	1	1501	F (1, 30) = 4.903	P=0.0346
Diet	11753	2	5876	F (2, 30) = 19.20	P<0.0001
Residual	9184	30	306.1		
Difference between row means					
Mean of Males	115.4				
Mean of Females	102.5				
Difference between means	12.91				
SE of difference	5.832				
95% CI of difference	1.003 to 24.82				
Data summary					
Number of columns (Diet)	3				
Number of rows (Sex)	2				
Number of values	36				
Number of families	1				
Number of comparisons per family	15				
Alpha	0.05				
Tukey's multiple comparisons test	Mean Diff.	95.00% CI of diff.	Below threshold?	Summary	Adjusted P Value
Males:Iron Adequate (IA) vs. Males:Iron Deficient (ID)	48.23	17.50 to 78.95	Yes	***	0.0006
Males:Iron Adequate (IA) vs. Males:ID+IA	47.36	16.63 to 78.08	Yes	***	0.0007
Males:Iron Adequate (IA) vs. Females:Iron Adequate (IA)	26.09	-4.638 to 56.81	No	ns	0.133
Males:Iron Adequate (IA) vs. Females:Iron Deficient (ID)	60.19	29.46 to 90.91	Yes	****	<0.0001
Males:Iron Adequate (IA) vs. Females:ID+IA	48.05	17.33 to 78.78	Yes	***	0.0006
Males:Iron Deficient (ID) vs. Males:ID+IA	-0.8683	-31.59 to 29.86	No	ns	>0.9999
Males:Iron Deficient (ID) vs. Females:Iron Adequate (IA)	-22.14	-52.87 to 8.585	No	ns	0.2711
Males:Iron Deficient (ID) vs. Females:Iron Deficient (ID)	11.96	-18.77 to 42.69	No	ns	0.8409
Males:Iron Deficient (ID) vs. Females:ID+IA	-0.1733	-30.90 to 30.55	No	ns	>0.9999
Males:ID+IA vs. Females:Iron Adequate (IA)	-21.27	-52.00 to 9.453	No	ns	0.3115
Males:ID+IA vs. Females:Iron Deficient (ID)	12.83	-17.90 to 43.55	No	ns	0.7985
Males:ID+IA vs. Females:ID+IA	0.695	-30.03 to 31.42	No	ns	>0.9999
Females:Iron Adequate (IA) vs. Females:Iron Deficient (ID)	34.1	3.375 to 64.83	Yes	*	0.0228
Females:Iron Adequate (IA) vs. Females:ID+IA	21.97	-8.758 to 52.69	No	ns	0.2789
Females:Iron Deficient (ID) vs. Females:ID+IA	-12.13	-42.86 to 18.59	No	ns	0.8328

Test details	Mean 1	Mean 2	Mean Diff.	SE of diff.	N1	N2	q	DF
Males:Iron Adequate (IA) vs. Males:Iron Deficient (ID)	147.3	99.06	48.23	10.1	6	6	6.752	30
Males:Iron Adequate (IA) vs. Males:ID+IA	147.3	99.93	47.36	10.1	6	6	6.63	30
Males:Iron Adequate (IA) vs. Females:Iron Adequate (IA)	147.3	121.2	26.09	10.1	6	6	3.652	30
Males:Iron Adequate (IA) vs. Females:Iron Deficient (ID)	147.3	87.1	60.19	10.1	6	6	8.426	30
Males:Iron Adequate (IA) vs. Females:ID+IA	147.3	99.24	48.05	10.1	6	6	6.727	30
Males:Iron Deficient (ID) vs. Males:ID+IA	99.06	99.93	-0.8683	10.1	6	6	0.1216	30
Males:Iron Deficient (ID) vs. Females:Iron Adequate (IA)	99.06	121.2	-22.14	10.1	6	6	3.1	30
Males:Iron Deficient (ID) vs. Females:Iron Deficient (ID)	99.06	87.1	11.96	10.1	6	6	1.674	30
Males:Iron Deficient (ID) vs. Females:ID+IA	99.06	99.24	-0.1733	10.1	6	6	0.02427	30
Males:ID+IA vs. Females:Iron Adequate (IA)	99.93	121.2	-21.27	10.1	6	6	2.978	30
Males:ID+IA vs. Females:Iron Deficient (ID)	99.93	87.1	12.83	10.1	6	6	1.796	30
Males:ID+IA vs. Females:ID+IA	99.93	99.24	0.695	10.1	6	6	0.0973	30
Females:Iron Adequate (IA) vs. Females:Iron Deficient (ID)	121.2	87.1	34.1	10.1	6	6	4.774	30
Females:Iron Adequate (IA) vs. Females:ID+IA	121.2	99.24	21.97	10.1	6	6	3.075	30
Females:Iron Deficient (ID) vs. Females:ID+IA	87.1	99.24	-12.13	10.1	6	6	1.699	30

Figure 3E

Table Analyzed	**Figure 3E** LD X diets							
Two-way ANOVA	Ordinary							
Alpha	0.05							
Source of Variation	% of total variatic	P value	P value summary	Significant?				
Interaction	14.16	0.0015	**	Yes				
Sex	29.41	<0.0001	****	Yes				
Diet	30.41	<0.0001	****	Yes				
ANOVA table	SS	DF	MS	F (DFn, DFd)	P value			
Interaction	8911	2	4456	F (2, 30) = 8.166	P=0.0015			
Sex	18504	1	18504	F (1, 30) = 33.92	P<0.0001			
Diet	19134	2	9567	F (2, 30) = 17.53	P<0.0001			
Residual	16368	30	545.6					
Difference between row means								
Mean of Males	149.1							
Mean of Females	103.7							
Difference between means	45.34							
SE of difference	7.786							
95% CI of difference	29.44 to 61.24							
Data summary								
Number of columns (Diet)	3							
Number of rows (Sex)	2							
Number of values	36							
Number of families	1							
Number of comparisons per family	15							
Alpha	0.05							
Tukey's multiple comparisons test	Mean Diff.	95.00% CI of diff.	Below threshold'	Summary	Adjusted P Value			
Males:Iron Adequate (IA) vs. Males:Iron Deficier	49.95	8.930 to 90.97	Yes	*	0.0101			
Males:Iron Adequate (IA) vs. Males:ID+IA	-41.95	-82.97 to -0.9333	Yes	*	0.0427			
Males:Iron Adequate (IA) vs. Females:Iron Adec	34.09	-6.927 to 75.11	No	ns	0.148			
Males:Iron Adequate (IA) vs. Females:Iron Defic	63.63	22.61 to 104.7	Yes	***	0.0007			
Males:Iron Adequate (IA) vs. Females:ID+IA	46.3	5.285 to 87.32	Yes	*	0.0198			
Males:Iron Deficient (ID) vs. Males:ID+IA	-91.9	-132.9 to -50.88	Yes	****	<0.0001			
Males:Iron Deficient (ID) vs. Females:Iron Adequ	-15.86	-56.88 to 25.16	No	ns	0.8447			
Males:Iron Deficient (ID) vs. Females:Iron Defici	13.68	-27.34 to 54.70	No	ns	0.9094			
Males:Iron Deficient (ID) vs. Females:ID+IA	-3.645	-44.66 to 37.37	No	ns	0.9998			
Males:ID+IA vs. Females:Iron Adequate (IA)	76.04	35.02 to 117.1	Yes	****	<0.0001			
Males:ID+IA vs. Females:Iron Deficient (ID)	105.6	64.56 to 146.6	Yes	****	<0.0001			
Males:ID+IA vs. Females:ID+IA	88.26	47.24 to 129.3	Yes	****	<0.0001			
Females:Iron Adequate (IA) vs. Females:Iron De	29.54	-11.48 to 70.56	No	ns	0.2717			
Females:Iron Adequate (IA) vs. Females:ID+IA	12.21	-28.81 to 53.23	No	ns	0.9421			
Females:Iron Deficient (ID) vs. Females:ID+IA	-17.33	-58.35 to 23.69	No	ns	0.7907			
Test details	Mean 1	Mean 2	Mean Diff.	SE of diff.	N1	N2	q	DF
Males:Iron Adequate (IA) vs. Males:Iron Deficier	151.7	101.8	49.95	13.49	6	6	5.238	30
Males:Iron Adequate (IA) vs. Males:ID+IA	151.7	193.7	-41.95	13.49	6	6	4.399	30
Males:Iron Adequate (IA) vs. Females:Iron Adec	151.7	117.6	34.09	13.49	6	6	3.575	30
Males:Iron Adequate (IA) vs. Females:Iron Defic	151.7	88.09	63.63	13.49	6	6	6.673	30
Males:Iron Adequate (IA) vs. Females:ID+IA	151.7	105.4	46.3	13.49	6	6	4.856	30
Males:Iron Deficient (ID) vs. Males:ID+IA	101.8	193.7	-91.9	13.49	6	6	9.637	30
Males:Iron Deficient (ID) vs. Females:Iron Adequ	101.8	117.6	-15.86	13.49	6	6	1.663	30
Males:Iron Deficient (ID) vs. Females:Iron Defici	101.8	88.09	13.68	13.49	6	6	1.435	30
Males:Iron Deficient (ID) vs. Females:ID+IA	101.8	105.4	-3.645	13.49	6	6	0.3822	30
Males:ID+IA vs. Females:Iron Adequate (IA)	193.7	117.6	76.04	13.49	6	6	7.974	30
Males:ID+IA vs. Females:Iron Deficient (ID)	193.7	88.09	105.6	13.49	6	6	11.07	30
Males:ID+IA vs. Females:ID+IA	193.7	105.4	88.26	13.49	6	6	9.255	30
Females:Iron Adequate (IA) vs. Females:Iron De	117.6	88.09	29.54	13.49	6	6	3.098	30
Females:Iron Adequate (IA) vs. Females:ID+IA	117.6	105.4	12.21	13.49	6	6	1.281	30
Females:Iron Deficient (ID) vs. Females:ID+IA	88.09	105.4	-17.33	13.49	6	6	1.817	30

Figure 3F

Table Analyzed	**Figure 3F** Sel X diets							
Two-way ANOVA	Ordinary							
Alpha	0.05							
Source of Variation	% of total variation	P value	P value summar	Significant?				
Interaction	0.07208	0.9769	ns	No				
Sex	5.81	0.0615	ns	No				
Diet	47.93	<0.0001	****	Yes				
ANOVA table	SS	DF	MS	F (DFn, DFd)	P value			
Interaction	17.96	2	8.979	F (2, 30) = 0.02341	P=0.9769			
Sex	1447	1	1447	F (1, 30) = 3.774	P=0.0615			
Diet	11940	2	5970	F (2, 30) = 15.57	P<0.0001			
Residual	11507	30	383.6					
Difference between row means								
Mean of Males	118.8							
Mean of Females	106.1							
Difference between means	12.68							
SE of difference	6.528							
95% CI of difference	-0.6506 to 26.01							
Data summary								
Number of columns (Diet)	3							
Number of rows (Sex)	2							
Number of values	36							
Number of families	1							
Number of comparisons per family	15							
Alpha	0.05							
Tukey's multiple comparisons test	Mean Diff.	95.00% CI of diff.	Below threshold	Summary	Adjusted P Value			
Males:Iron Adequate (IA) vs. Males:Iron Deficient (ID)	43.24	8.850 to 77.63	Yes	**	0.0074			
Males:Iron Adequate (IA) vs. Males:ID+IA	33.68	-0.7133 to 68.07	No	ns	0.0576			
Males:Iron Adequate (IA) vs. Females:Iron Adequate (	13.27	-21.12 to 47.66	No	ns	0.8458			
Males:Iron Adequate (IA) vs. Females:Iron Deficient (I	53.98	19.59 to 88.37	Yes	***	0.0006			
Males:Iron Adequate (IA) vs. Females:ID+IA	47.72	13.33 to 82.11	Yes	**	0.0026			
Males:Iron Deficient (ID) vs. Males:ID+IA	-9.563	-43.95 to 24.83	No	ns	0.9562			
Males:Iron Deficient (ID) vs. Females:Iron Adequate (I	-29.98	-64.37 to 4.417	No	ns	0.1159			
Males:Iron Deficient (ID) vs. Females:Iron Deficient (ID	10.74	-23.66 to 45.13	No	ns	0.93			
Males:Iron Deficient (ID) vs. Females:ID+IA	4.48	-29.91 to 38.87	No	ns	0.9986			
Males:ID+IA vs. Females:Iron Adequate (IA)	-20.41	-54.80 to 13.98	No	ns	0.4777			
Males:ID+IA vs. Females:Iron Deficient (ID)	20.3	-14.09 to 54.69	No	ns	0.4838			
Males:ID+IA vs. Females:ID+IA	14.04	-20.35 to 48.43	No	ns	0.8128			
Females:Iron Adequate (IA) vs. Females:Iron Deficien	40.71	6.318 to 75.10	Yes	*	0.0131			
Females:Iron Adequate (IA) vs. Females:ID+IA	34.46	0.06340 to 68.85	Yes	*	0.0494			
Females:Iron Deficient (ID) vs. Females:ID+IA	-6.255	-40.65 to 28.14	No	ns	0.9933			
Test details	Mean 1	Mean 2	Mean Diff.	SE of diff.	N1	N2	q	DF
Males:Iron Adequate (IA) vs. Males:Iron Deficient (ID)	144.5	101.2	43.24	11.31	6	6	5.408	30
Males:Iron Adequate (IA) vs. Males:ID+IA	144.5	110.8	33.68	11.31	6	6	4.212	30
Males:Iron Adequate (IA) vs. Females:Iron Adequate (	144.5	131.2	13.27	11.31	6	6	1.659	30
Males:Iron Adequate (IA) vs. Females:Iron Deficient (I	144.5	90.49	53.98	11.31	6	6	6.751	30
Males:Iron Adequate (IA) vs. Females:ID+IA	144.5	96.75	47.72	11.31	6	6	5.969	30
Males:Iron Deficient (ID) vs. Males:ID+IA	101.2	110.8	-9.563	11.31	6	6	1.196	30
Males:Iron Deficient (ID) vs. Females:Iron Adequate (I	101.2	131.2	-29.98	11.31	6	6	3.749	30
Males:Iron Deficient (ID) vs. Females:Iron Deficient (ID	101.2	90.49	10.74	11.31	6	6	1.343	30
Males:Iron Deficient (ID) vs. Females:ID+IA	101.2	96.75	4.48	11.31	6	6	0.5603	30
Males:ID+IA vs. Females:Iron Adequate (IA)	110.8	131.2	-20.41	11.31	6	6	2.553	30
Males:ID+IA vs. Females:Iron Deficient (ID)	110.8	90.49	20.3	11.31	6	6	2.539	30
Males:ID+IA vs. Females:ID+IA	110.8	96.75	14.04	11.31	6	6	1.756	30
Females:Iron Adequate (IA) vs. Females:Iron Deficien	131.2	90.49	40.71	11.31	6	6	5.092	30
Females:Iron Adequate (IA) vs. Females:ID+IA	131.2	96.75	34.46	11.31	6	6	4.309	30
Females:Iron Deficient (ID) vs. Females:ID+IA	90.49	96.75	-6.255	11.31	6	6	0.7823	30

Figure 4B

Table Analyzed	**Figure 4B** Tfr1 WB Veh x diet				
Two-way ANOVA	Ordinary				
Alpha	0.05				
Source of Variation	% of total variation	P value	P value sun	Significant?	
Interaction	0.2713	0.8521	ns	No	
Sex	38.69	<0.0001	****	Yes	
Diet	51	<0.0001	****	Yes	
ANOVA table	SS	DF	MS	F (DFn, DFd)	P value
Interaction	0.003391	2	0.001696	F (2, 12) = 0.1621	P=0.8521
Sex	0.4837	1	0.4837	F (1, 12) = 46.25	P<0.0001
Diet	0.6376	2	0.3188	F (2, 12) = 30.48	P<0.0001
Residual	0.1255	12	0.01046		
Difference between row means					
Mean of Males	0.5598				
Mean of Females	0.8877				
Difference between means	-0.3278				
SE of difference	0.04821				
95% CI of difference	-0.4329 to -0.2228				
Data summary					
Number of columns (Diet)	3				
Number of rows (Sex)	2				
Number of values	18				
Number of families	1				
Number of comparisons per family	15				
Alpha	0.05				

Tukey's multiple comparisons test	Mean Diff.	95.00% CI of diff.	Below thres	Summary	Adjusted P Value
Males :Iron Adequate (IA) vs. Males :Iron Deficient (ID)	-0.307	-0.5875 to -0.02654	Yes	*	0.0293
Males :Iron Adequate (IA) vs. Males :Iron Repletion (IR)	-0.4264	-0.7068 to -0.1459	Yes	**	0.0027
Males :Iron Adequate (IA) vs. Females:Iron Adequate (IA)	-0.3137	-0.5941 to -0.03321	Yes	*	0.0256
Males :Iron Adequate (IA) vs. Females:Iron Deficient (ID)	-0.6106	-0.8911 to -0.3302	Yes	***	0.0001
Males :Iron Adequate (IA) vs. Females:Iron Repletion (IR)	-0.7926	-1.073 to -0.5121	Yes	****	<0.0001
Males :Iron Deficient (ID) vs. Males :Iron Repletion (IR)	-0.1194	-0.3998 to 0.1611	No	ns	0.7104
Males :Iron Deficient (ID) vs. Females:Iron Adequate (IA)	-0.006667	-0.2871 to 0.2738	No	ns	>0.9999
Males :Iron Deficient (ID) vs. Females:Iron Deficient (ID)	-0.3036	-0.5841 to -0.02317	Yes	*	0.0313
Males :Iron Deficient (ID) vs. Females:Iron Repletion (IR)	-0.4856	-0.7661 to -0.2051	Yes	***	0.0009
Males :Iron Repletion (IR) vs. Females:Iron Adequate (IA)	0.1127	-0.1678 to 0.3932	No	ns	0.7539
Males :Iron Repletion (IR) vs. Females:Iron Deficient (ID)	-0.1843	-0.4647 to 0.09619	No	ns	0.302
Males :Iron Repletion (IR) vs. Females:Iron Repletion (IR)	-0.3662	-0.6467 to -0.08577	Yes	**	0.0089
Females:Iron Adequate (IA) vs. Females:Iron Deficient (ID	-0.297	-0.5774 to -0.01651	Yes	*	0.0359
Females:Iron Adequate (IA) vs. Females:Iron Repletion (I	-0.4789	-0.7594 to -0.1985	Yes	**	0.001
Females:Iron Deficient (ID) vs. Females:Iron Repletion (IF	-0.182	-0.4624 to 0.09849	No	ns	0.3135

Test details	Mean 1	Mean 2	Mean Diff.	SE of diff.	N1	N2	q	DF
Males :Iron Adequate (IA) vs. Males :Iron Deficient (ID)	0.3154	0.6224	-0.307	0.0835	3	3	5.2	12
Males :Iron Adequate (IA) vs. Males :Iron Repletion (IR)	0.3154	0.7417	-0.4264	0.0835	3	3	7.221	12
Males :Iron Adequate (IA) vs. Females:Iron Adequate (IA)	0.3154	0.629	-0.3137	0.0835	3	3	5.313	12
Males :Iron Adequate (IA) vs. Females:Iron Deficient (ID)	0.3154	0.926	-0.6106	0.0835	3	3	10.34	12
Males :Iron Adequate (IA) vs. Females:Iron Repletion (IR)	0.3154	1.108	-0.7926	0.0835	3	3	13.42	12
Males :Iron Deficient (ID) vs. Males :Iron Repletion (IR)	0.6224	0.7417	-0.1194	0.0835	3	3	2.022	12
Males :Iron Deficient (ID) vs. Females:Iron Adequate (IA)	0.6224	0.629	-0.006667	0.0835	3	3	0.1129	12
Males :Iron Deficient (ID) vs. Females:Iron Deficient (ID)	0.6224	0.926	-0.3036	0.0835	3	3	5.143	12
Males :Iron Deficient (ID) vs. Females:Iron Repletion (IR)	0.6224	1.108	-0.4856	0.0835	3	3	8.225	12
Males :Iron Repletion (IR) vs. Females:Iron Adequate (IA)	0.7417	0.629	0.1127	0.0835	3	3	1.909	12
Males :Iron Repletion (IR) vs. Females:Iron Deficient (ID)	0.7417	0.926	-0.1843	0.0835	3	3	3.121	12
Males :Iron Repletion (IR) vs. Females:Iron Repletion (IR)	0.7417	1.108	-0.3662	0.0835	3	3	6.203	12
Females:Iron Adequate (IA) vs. Females:Iron Deficient (ID	0.629	0.926	-0.297	0.0835	3	3	5.03	12
Females:Iron Adequate (IA) vs. Females:Iron Repletion (I	0.629	1.108	-0.4789	0.0835	3	3	8.112	12
Females:Iron Deficient (ID) vs. Females:Iron Repletion (IF	0.926	1.108	-0.182	0.0835	3	3	3.082	12

Figure 4C

Table Analyzed	**Figure 4C** Tfr1 WB LD x diet							
Two-way ANOVA	Ordinary							
Alpha	0.05							
Source of Variation	% of total va	P value	P value sun	Significant?				
Interaction	30.2	0.0291	*	Yes				
Sex	1.216	0.5449	ns	No				
Diet	31	0.0271	*	Yes				
ANOVA table	SS	DF	MS	F (DFn, DFd)	P value			
Interaction	0.178	2	0.08901	F (2, 12) = 4.821	P=0.0291			
Sex	0.007168	1	0.007168	F (1, 12) = 0.3883	P=0.5449			
Diet	0.1827	2	0.09137	F (2, 12) = 4.950	P=0.0271			
Residual	0.2215	12	0.01846					
Difference between row means								
Mean of Males	0.8368							
Mean of Females	0.8767							
Difference between means	-0.03991							
SE of difference	0.06405							
95% CI of difference	-0.1795 to 0.09964							
Data summary								
Number of columns (Diet)	3							
Number of rows (Sex)	2							
Number of values	18							
Number of families	1							
Number of comparisons per family	15							
Alpha	0.05							
Tukey's multiple comparisons test	Mean Diff.	95.00% CI of diff.	Below thres	Summary	Adjusted P Value			
Males :Iron Adequate (IA) vs. Males :Iron Deficient (ID)	-0.2513	-0.6239 to 0.1213	No	ns	0.2787			
Males :Iron Adequate (IA) vs. Males :Iron Repletion (IR)	-0.4489	-0.8215 to -0.07627	Yes	*	0.0157			
Males :Iron Adequate (IA) vs. Females:Iron Adequate (IA)	-0.3209	-0.6936 to 0.05170	No	ns	0.1077			
Males :Iron Adequate (IA) vs. Females:Iron Deficient (ID)	-0.1611	-0.5338 to 0.2115	No	ns	0.6976			
Males :Iron Adequate (IA) vs. Females:Iron Repletion (IR)	-0.3379	-0.7105 to 0.03477	No	ns	0.0841			
Males :Iron Deficient (ID) vs. Males :Iron Repletion (IR)	-0.1976	-0.5702 to 0.1750	No	ns	0.5105			
Males :Iron Deficient (ID) vs. Females:Iron Adequate (IA)	-0.06963	-0.4423 to 0.3030	No	ns	0.9866			
Males :Iron Deficient (ID) vs. Females:Iron Deficient (ID)	0.09017	-0.2825 to 0.4628	No	ns	0.9596			
Males :Iron Deficient (ID) vs. Females:Iron Repletion (IR)	-0.08657	-0.4592 to 0.2861	No	ns	0.9658			
Males :Iron Repletion (IR) vs. Females:Iron Adequate (IA)	0.128	-0.2447 to 0.5006	No	ns	0.8498			
Males :Iron Repletion (IR) vs. Females:Iron Deficient (ID)	0.2878	-0.08487 to 0.6604	No	ns	0.1722			
Males :Iron Repletion (IR) vs. Females:Iron Repletion (IR)	0.111	-0.2616 to 0.4837	No	ns	0.909			
Females:Iron Adequate (IA) vs. Females:Iron Deficient (ID)	0.1598	-0.2128 to 0.5324	No	ns	0.7044			
Females:Iron Adequate (IA) vs. Females:Iron Repletion (IR)	-0.01693	-0.3896 to 0.3557	No	ns	>0.9999			
Females:Iron Deficient (ID) vs. Females:Iron Repletion (IR)	-0.1767	-0.5494 to 0.1959	No	ns	0.6174			
Test details	Mean 1	Mean 2	Mean Diff.	SE of diff.	N1	N2	q	DF
Males :Iron Adequate (IA) vs. Males :Iron Deficient (ID)	0.6034	0.8547	-0.2513	0.1109	3	3	3.203	12
Males :Iron Adequate (IA) vs. Males :Iron Repletion (IR)	0.6034	1.052	-0.4489	0.1109	3	3	5.722	12
Males :Iron Adequate (IA) vs. Females:Iron Adequate (IA)	0.6034	0.9243	-0.3209	0.1109	3	3	4.091	12
Males :Iron Adequate (IA) vs. Females:Iron Deficient (ID)	0.6034	0.7645	-0.1611	0.1109	3	3	2.054	12
Males :Iron Adequate (IA) vs. Females:Iron Repletion (IR)	0.6034	0.9412	-0.3379	0.1109	3	3	4.307	12
Males :Iron Deficient (ID) vs. Males :Iron Repletion (IR)	0.8547	1.052	-0.1976	0.1109	3	3	2.519	12
Males :Iron Deficient (ID) vs. Females:Iron Adequate (IA)	0.8547	0.9243	-0.06963	0.1109	3	3	0.8877	12
Males :Iron Deficient (ID) vs. Females:Iron Deficient (ID)	0.8547	0.7645	0.09017	0.1109	3	3	1.149	12
Males :Iron Deficient (ID) vs. Females:Iron Repletion (IR)	0.8547	0.9412	-0.08657	0.1109	3	3	1.104	12
Males :Iron Repletion (IR) vs. Females:Iron Adequate (IA)	1.052	0.9243	0.128	0.1109	3	3	1.631	12
Males :Iron Repletion (IR) vs. Females:Iron Deficient (ID)	1.052	0.7645	0.2878	0.1109	3	3	3.668	12
Males :Iron Repletion (IR) vs. Females:Iron Repletion (IR)	1.052	0.9412	0.111	0.1109	3	3	1.415	12
Females:Iron Adequate (IA) vs. Females:Iron Deficient (ID)	0.9243	0.7645	0.1598	0.1109	3	3	2.037	12
Females:Iron Adequate (IA) vs. Females:Iron Repletion (IR)	0.9243	0.9412	-0.01693	0.1109	3	3	0.2159	12
Females:Iron Deficient (ID) vs. Females:Iron Repletion (IR)	0.7645	0.9412	-0.1767	0.1109	3	3	2.253	12

Figure 4D

Table Analyzed	**Figure 4D** Tfr1 WB Sel x diet							
Two-way ANOVA	Ordinary							
Alpha	0.05							
Source of Variation	% of total varia	P value	P value sun	Significant?				
Interaction	0.7952	0.8685	ns	No				
Sex	16.47	0.0317	*	Yes				
Diet	49.28	0.0044	**	Yes				
ANOVA table	SS	DF	MS	F (DFn, DFd)	P value			
Interaction	0.00779	2	0.003895	F (2, 12) = 0.1426	P=0.8685			
Sex	0.1614	1	0.1614	F (1, 12) = 5.909	P=0.0317			
Diet	0.4828	2	0.2414	F (2, 12) = 8.838	P=0.0044			
Residual	0.3278	12	0.02731					
Difference between row means								
Mean of Males	0.808							
Mean of Females	0.9974							
Difference between means	-0.1894							
SE of difference	0.07791							
95% CI of difference	-0.3591 to -0.01963							
Data summary								
Number of columns (Diet)	3							
Number of rows (Sex)	2							
Number of values	18							
Number of families	1							
Number of comparisons per family	15							
Alpha	0.05							
Tukey's multiple comparisons test	Mean Diff.	95.00% CI of diff.	Below thres	Summary	Adjusted P Value			
Males :Iron Adequate (IA) vs. Males :Iron Deficient (ID)	-0.1517	-0.6050 to 0.3015	No	ns	0.8622			
Males :Iron Adequate (IA) vs. Males :Iron Repletion (IR)	0.2443	-0.2090 to 0.6976	No	ns	0.4944			
Males :Iron Adequate (IA) vs. Females:Iron Adequate (IA)	-0.1308	-0.5841 to 0.3224	No	ns	0.9191			
Males :Iron Adequate (IA) vs. Females:Iron Deficient (ID)	-0.3755	-0.8288 to 0.07775	No	ns	0.1284			
Males :Iron Adequate (IA) vs. Females:Iron Repletion (IR)	0.03077	-0.4225 to 0.4840	No	ns	0.9999			
Males :Iron Deficient (ID) vs. Males :Iron Repletion (IR)	0.396	-0.05722 to 0.8493	No	ns	0.1006			
Males :Iron Deficient (ID) vs. Females:Iron Adequate (IA)	0.0209	-0.4324 to 0.4742	No	ns	>0.9999			
Males :Iron Deficient (ID) vs. Females:Iron Deficient (ID)	-0.2238	-0.6770 to 0.2295	No	ns	0.58			
Males :Iron Deficient (ID) vs. Females:Iron Repletion (IR)	0.1825	-0.2708 to 0.6358	No	ns	0.7524			
Males :Iron Repletion (IR) vs. Females:Iron Adequate (IA)	-0.3751	-0.8284 to 0.07812	No	ns	0.1289			
Males :Iron Repletion (IR) vs. Females:Iron Deficient (ID)	-0.6198	-1.073 to -0.1665	Yes	**	0.0063			
Males :Iron Repletion (IR) vs. Females:Iron Repletion (IR)	-0.2135	-0.6668 to 0.2397	No	ns	0.6235			
Females:Iron Adequate (IA) vs. Females:Iron Deficient (ID	-0.2447	-0.6979 to 0.2086	No	ns	0.4929			
Females:Iron Adequate (IA) vs. Females:Iron Repletion (IF	0.1616	-0.2917 to 0.6149	No	ns	0.83			
Females:Iron Deficient (ID) vs. Females:Iron Repletion (IR	0.4063	-0.04698 to 0.8595	No	ns	0.089			
Test details	Mean 1	Mean 2	Mean Diff.	SE of diff.	N1	N2	q	DF
Males :Iron Adequate (IA) vs. Males :Iron Deficient (ID)	0.8388	0.9906	-0.1517	0.1349	3	3	1.59	12
Males :Iron Adequate (IA) vs. Males :Iron Repletion (IR)	0.8388	0.5945	0.2443	0.1349	3	3	2.56	12
Males :Iron Adequate (IA) vs. Females:Iron Adequate (IA)	0.8388	0.9697	-0.1308	0.1349	3	3	1.371	12
Males :Iron Adequate (IA) vs. Females:Iron Deficient (ID)	0.8388	1.214	-0.3755	0.1349	3	3	3.935	12
Males :Iron Adequate (IA) vs. Females:Iron Repletion (IR)	0.8388	0.8081	0.03077	0.1349	3	3	0.3224	12
Males :Iron Deficient (ID) vs. Males :Iron Repletion (IR)	0.9906	0.5945	0.396	0.1349	3	3	4.151	12
Males :Iron Deficient (ID) vs. Females:Iron Adequate (IA)	0.9906	0.9697	0.0209	0.1349	3	3	0.219	12
Males :Iron Deficient (ID) vs. Females:Iron Deficient (ID)	0.9906	1.214	-0.2238	0.1349	3	3	2.345	12
Males :Iron Deficient (ID) vs. Females:Iron Repletion (IR)	0.9906	0.8081	0.1825	0.1349	3	3	1.913	12
Males :Iron Repletion (IR) vs. Females:Iron Adequate (IA)	0.5945	0.9697	-0.3751	0.1349	3	3	3.932	12
Males :Iron Repletion (IR) vs. Females:Iron Deficient (ID)	0.5945	1.214	-0.6198	0.1349	3	3	6.496	12
Males :Iron Repletion (IR) vs. Females:Iron Repletion (IR)	0.5945	0.8081	-0.2135	0.1349	3	3	2.238	12
Females:Iron Adequate (IA) vs. Females:Iron Deficient (ID	0.9697	1.214	-0.2447	0.1349	3	3	2.564	12
Females:Iron Adequate (IA) vs. Females:Iron Repletion (IF	0.9697	0.8081	0.1616	0.1349	3	3	1.694	12
Females:Iron Deficient (ID) vs. Females:Iron Repletion (IR	1.214	0.8081	0.4063	0.1349	3	3	4.258	12

Figure 4E

Table Analyzed	**Figure 4E** FPN1 WB Veh x diet							
Two-way ANOVA	Ordinary							
Alpha	0.05							
Source of Variation	% of total variation	P value	P value sun	Significant?				
Interaction	21.68	0.0023	**	Yes				
Sex	35.73	<0.0001	****	Yes				
Diet	30.29	0.0006	***	Yes				
ANOVA table	SS	DF	MS	F (DFn, DFd)	P value			
Interaction	0.1104	2	0.05521	F (2, 12) = 10.57	P=0.0023			
Sex	0.182	1	0.182	F (1, 12) = 34.86	P<0.0001			
Diet	0.1543	2	0.07714	F (2, 12) = 14.77	P=0.0006			
Residual	0.06266	12	0.005222					
Difference between row means								
Mean of Males	0.7116							
Mean of Females	0.9127							
Difference between means	-0.2011							
SE of difference	0.03406							
95% CI of difference	-0.2753 to -0.1269							
Data summary								
Number of columns (Diet)	3							
Number of rows (Sex)	2							
Number of values	18							
Number of families	1							
Number of comparisons per family	15							
Alpha	0.05							
Tukey's multiple comparisons test	Mean Diff.	95.00% CI of diff.	Below thres	Summary	Adjusted P Value			
Males :Iron Adequate (IA) vs. Males :Iron Deficient (ID)	0.05827	-0.1399 to 0.2564	No	ns	0.9134			
Males :Iron Adequate (IA) vs. Males :Iron Repletion (IR)	0.004667	-0.1935 to 0.2028	No	ns	>0.9999			
Males :Iron Adequate (IA) vs. Females:Iron Adequate (IA)	-0.4197	-0.6179 to -0.2216	Yes	***	0.0001			
Males :Iron Adequate (IA) vs. Females:Iron Deficient (ID)	-0.06463	-0.2628 to 0.1335	No	ns	0.8741			
Males :Iron Adequate (IA) vs. Females:Iron Repletion (IR)	-0.05607	-0.2542 to 0.1421	No	ns	0.9251			
Males :Iron Deficient (ID) vs. Males :Iron Repletion (IR)	-0.0536	-0.2518 to 0.1446	No	ns	0.937			
Males :Iron Deficient (ID) vs. Females:Iron Adequate (IA)	-0.478	-0.6762 to -0.2798	Yes	****	<0.0001			
Males :Iron Deficient (ID) vs. Females:Iron Deficient (ID)	-0.1229	-0.3211 to 0.07528	No	ns	0.356			
Males :Iron Deficient (ID) vs. Females:Iron Repletion (IR)	-0.1143	-0.3125 to 0.08385	No	ns	0.4267			
Males :Iron Repletion (IR) vs. Females:Iron Adequate (IA)	-0.4244	-0.6226 to -0.2262	Yes	***	0.0001			
Males :Iron Repletion (IR) vs. Females:Iron Deficient (ID)	-0.0693	-0.2675 to 0.1289	No	ns	0.8405			
Males :Iron Repletion (IR) vs. Females:Iron Repletion (IR)	-0.06073	-0.2589 to 0.1374	No	ns	0.8991			
Females:Iron Adequate (IA) vs. Females:Iron Deficient (ID)	0.3551	0.1569 to 0.5533	Yes	***	0.0007			
Females:Iron Adequate (IA) vs. Females:Iron Repletion (IR	0.3637	0.1655 to 0.5618	Yes	***	0.0005			
Females:Iron Deficient (ID) vs. Females:Iron Repletion (IR)	0.008567	-0.1896 to 0.2067	No	ns	>0.9999			
Test details	Mean 1	Mean 2	Mean Diff.	SE of diff.	N1	N2	q	DF
Males :Iron Adequate (IA) vs. Males :Iron Deficient (ID)	0.7325	0.6743	0.05827	0.059	3	3	1.397	12
Males :Iron Adequate (IA) vs. Males :Iron Repletion (IR)	0.7325	0.7279	0.004667	0.059	3	3	0.1119	12
Males :Iron Adequate (IA) vs. Females:Iron Adequate (IA)	0.7325	1.152	-0.4197	0.059	3	3	10.06	12
Males :Iron Adequate (IA) vs. Females:Iron Deficient (ID)	0.7325	0.7972	-0.06463	0.059	3	3	1.549	12
Males :Iron Adequate (IA) vs. Females:Iron Repletion (IR)	0.7325	0.7886	-0.05607	0.059	3	3	1.344	12
Males :Iron Deficient (ID) vs. Males :Iron Repletion (IR)	0.6743	0.7279	-0.0536	0.059	3	3	1.285	12
Males :Iron Deficient (ID) vs. Females:Iron Adequate (IA)	0.6743	1.152	-0.478	0.059	3	3	11.46	12
Males :Iron Deficient (ID) vs. Females:Iron Deficient (ID)	0.6743	0.7972	-0.1229	0.059	3	3	2.946	12
Males :Iron Deficient (ID) vs. Females:Iron Repletion (IR)	0.6743	0.7886	-0.1143	0.059	3	3	2.74	12
Males :Iron Repletion (IR) vs. Females:Iron Adequate (IA)	0.7279	1.152	-0.4244	0.059	3	3	10.17	12
Males :Iron Repletion (IR) vs. Females:Iron Deficient (ID)	0.7279	0.7972	-0.0693	0.059	3	3	1.661	12
Males :Iron Repletion (IR) vs. Females:Iron Repletion (IR)	0.7279	0.7886	-0.06073	0.059	3	3	1.456	12
Females:Iron Adequate (IA) vs. Females:Iron Deficient (ID)	1.152	0.7972	0.3551	0.059	3	3	8.511	12
Females:Iron Adequate (IA) vs. Females:Iron Repletion (IR	1.152	0.7886	0.3637	0.059	3	3	8.717	12
Females:Iron Deficient (ID) vs. Females:Iron Repletion (IR)	0.7972	0.7886	0.008567	0.059	3	3	0.2053	12

Figure 4F

Table Analyzed	**Figure 4F** FPN1 WB LD x diet							
Two-way ANOVA	Ordinary							
Alpha	0.05							
Source of Variation	% of total variation	P value	P value sun	Significant?				
Interaction	33.43	0.0047	**	Yes				
Sex	5.947	0.1044	ns	No				
Diet	37.5	0.0031	**	Yes				
ANOVA table	SS	DF	MS	F (DFn, DFd)	P value			
Interaction	0.1406	2	0.0703	F (2, 12) = 8.676	P=0.0047			
Sex	0.02501	1	0.02501	F (1, 12) = 3.086	P=0.1044			
Diet	0.1577	2	0.07884	F (2, 12) = 9.730	P=0.0031			
Residual	0.09723	12	0.008103					
Difference between row means								
Mean of Males	0.8551							
Mean of Females	0.9296							
Difference between means	-0.07455							
SE of difference	0.04243							
95% CI of difference	-0.1670 to 0.01791							
Data summary								
Number of columns (Diet)	3							
Number of rows (Sex)	2							
Number of values	18							
Number of families	1							
Number of comparisons per family	15							
Alpha	0.05							
Tukey's multiple comparisons test	Mean Diff.	95.00% CI of diff.	Below thres	Summary	Adjusted P Value			
Males :Iron Adequate (IA) vs. Males :Iron Deficient (ID)	-0.1625	-0.4094 to 0.08438	No	ns	0.3003			
Males :Iron Adequate (IA) vs. Males :Iron Repletion (IR)	-0.4407	-0.6876 to -0.1938	Yes	***	0.0007			
Males :Iron Adequate (IA) vs. Females:Iron Adequate (IA	-0.2692	-0.5161 to -0.02233	Yes	*	0.03			
Males :Iron Adequate (IA) vs. Females:Iron Deficient (ID)	-0.2755	-0.5224 to -0.02866	Yes	*	0.0259			
Males :Iron Adequate (IA) vs. Females:Iron Repletion (IR	-0.2821	-0.5290 to -0.03523	Yes	*	0.0223			
Males :Iron Deficient (ID) vs. Males :Iron Repletion (IR)	-0.2782	-0.5251 to -0.03133	Yes	*	0.0244			
Males :Iron Deficient (ID) vs. Females:Iron Adequate (IA)	-0.1067	-0.3536 to 0.1402	No	ns	0.698			
Males :Iron Deficient (ID) vs. Females:Iron Deficient (ID)	-0.113	-0.3599 to 0.1338	No	ns	0.6491			
Males :Iron Deficient (ID) vs. Females:Iron Repletion (IR)	-0.1196	-0.3665 to 0.1273	No	ns	0.5977			
Males :Iron Repletion (IR) vs. Females:Iron Adequate (IA	0.1715	-0.07537 to 0.4184	No	ns	0.2531			
Males :Iron Repletion (IR) vs. Females:Iron Deficient (ID)	0.1652	-0.08171 to 0.4120	No	ns	0.2857			
Males :Iron Repletion (IR) vs. Females:Iron Repletion (IR	0.1586	-0.08827 to 0.4055	No	ns	0.3226			
Females:Iron Adequate (IA) vs. Females:Iron Deficient (II	-0.006333	-0.2532 to 0.2405	No	ns	>0.9999			
Females:Iron Adequate (IA) vs. Females:Iron Repletion (	-0.0129	-0.2598 to 0.2340	No	ns	>0.9999			
Females:Iron Deficient (ID) vs. Females:Iron Repletion (I	-0.006567	-0.2534 to 0.2403	No	ns	>0.9999			
Test details	Mean 1	Mean 2	Mean Diff.	SE of diff.	N1	N2	q	DF
Males :Iron Adequate (IA) vs. Males :Iron Deficient (ID)	0.654	0.8165	-0.1625	0.0735	3	3	3.127	12
Males :Iron Adequate (IA) vs. Males :Iron Repletion (IR)	0.654	1.095	-0.4407	0.0735	3	3	8.48	12
Males :Iron Adequate (IA) vs. Females:Iron Adequate (IA	0.654	0.9232	-0.2692	0.0735	3	3	5.18	12
Males :Iron Adequate (IA) vs. Females:Iron Deficient (ID)	0.654	0.9295	-0.2755	0.0735	3	3	5.302	12
Males :Iron Adequate (IA) vs. Females:Iron Repletion (IR	0.654	0.9361	-0.2821	0.0735	3	3	5.428	12
Males :Iron Deficient (ID) vs. Males :Iron Repletion (IR)	0.8165	1.095	-0.2782	0.0735	3	3	5.353	12
Males :Iron Deficient (ID) vs. Females:Iron Adequate (IA)	0.8165	0.9232	-0.1067	0.0735	3	3	2.053	12
Males :Iron Deficient (ID) vs. Females:Iron Deficient (ID)	0.8165	0.9295	-0.113	0.0735	3	3	2.175	12
Males :Iron Deficient (ID) vs. Females:Iron Repletion (IR)	0.8165	0.9361	-0.1196	0.0735	3	3	2.301	12
Males :Iron Repletion (IR) vs. Females:Iron Adequate (IA	1.095	0.9232	0.1715	0.0735	3	3	3.3	12
Males :Iron Repletion (IR) vs. Females:Iron Deficient (ID)	1.095	0.9295	0.1652	0.0735	3	3	3.178	12
Males :Iron Repletion (IR) vs. Females:Iron Repletion (IR	1.095	0.9361	0.1586	0.0735	3	3	3.052	12
Females:Iron Adequate (IA) vs. Females:Iron Deficient (II	0.9232	0.9295	-0.006333	0.0735	3	3	0.1219	12
Females:Iron Adequate (IA) vs. Females:Iron Repletion (	0.9232	0.9361	-0.0129	0.0735	3	3	0.2482	12
Females:Iron Deficient (ID) vs. Females:Iron Repletion (I	0.9295	0.9361	-0.006567	0.0735	3	3	0.1264	12

Figure 4G

Table Analyzed	**Figure 4G** FPN1 WB Sel x diet							
Two-way ANOVA	Ordinary							
Alpha	0.05							
Source of Variation	% of total variation	P value	P value sum	Significant?				
Interaction	15.92	0.0006	***	Yes				
Sex	28.16	<0.0001	****	Yes				
Diet	49.46	<0.0001	****	Yes				
ANOVA table	SS	DF	MS	F (DFn, DFd)	P value			
Interaction	0.1069	2	0.05345	F (2, 12) = 14.80	P=0.0006			
Sex	0.189	1	0.189	F (1, 12) = 52.34	P<0.0001			
Diet	0.3321	2	0.166	F (2, 12) = 45.97	P<0.0001			
Residual	0.04334	12	0.003612					
Difference between row means								
Mean of Males	1.124							
Mean of Females	0.919							
Difference between means	0.205							
SE of difference	0.02833							
95% CI of difference	0.1432 to 0.2667							
Data summary								
Number of columns (Diet)	3							
Number of rows (Sex)	2							
Number of values	18							
Number of families	1							
Number of comparisons per family	15							
Alpha	0.05							
Tukey's multiple comparisons test	Mean Diff.	95.00% CI of diff.	Below thres	Summary	Adjusted P Value			
Males :Iron Adequate (IA) vs. Males :Iron Deficient (ID)	0.2541	0.08925 to 0.4189	Yes	**	0.0024			
Males :Iron Adequate (IA) vs. Males :Iron Repletion (IR)	0.5165	0.3516 to 0.6813	Yes	****	<0.0001			
Males :Iron Adequate (IA) vs. Females:Iron Adequate (IA)	0.3693	0.2044 to 0.5341	Yes	****	<0.0001			
Males :Iron Adequate (IA) vs. Females:Iron Deficient (ID)	0.5009	0.3361 to 0.6657	Yes	****	<0.0001			
Males :Iron Adequate (IA) vs. Females:Iron Repletion (IR)	0.5152	0.3504 to 0.6801	Yes	****	<0.0001			
Males :Iron Deficient (ID) vs. Males :Iron Repletion (IR)	0.2624	0.09757 to 0.4272	Yes	**	0.0019			
Males :Iron Deficient (ID) vs. Females:Iron Adequate (IA)	0.1152	-0.04963 to 0.2800	No	ns	0.2481			
Males :Iron Deficient (ID) vs. Females:Iron Deficient (ID)	0.2468	0.08201 to 0.4117	Yes	**	0.0031			
Males :Iron Deficient (ID) vs. Females:Iron Repletion (IR)	0.2612	0.09634 to 0.4260	Yes	**	0.0019			
Males :Iron Repletion (IR) vs. Females:Iron Adequate (IA)	-0.1472	-0.3120 to 0.01763	No	ns	0.0906			
Males :Iron Repletion (IR) vs. Females:Iron Deficient (ID)	-0.01557	-0.1804 to 0.1493	No	ns	0.9994			
Males :Iron Repletion (IR) vs. Females:Iron Repletion (IR)	-0.001233	-0.1661 to 0.1636	No	ns	>0.9999			
Females:Iron Adequate (IA) vs. Females:Iron Deficient (ID	0.1316	-0.03319 to 0.2965	No	ns	0.1503			
Females:Iron Adequate (IA) vs. Females:Iron Repletion (II	0.146	-0.01886 to 0.3108	No	ns	0.0944			
Females:Iron Deficient (ID) vs. Females:Iron Repletion (IR	0.01433	-0.1505 to 0.1792	No	ns	0.9996			
Test details	Mean 1	Mean 2	Mean Diff.	SE of diff.	N1	N2	q	DF
Males :Iron Adequate (IA) vs. Males :Iron Deficient (ID)	1.381	1.127	0.2541	0.04907	3	3	7.322	12
Males :Iron Adequate (IA) vs. Males :Iron Repletion (IR)	1.381	0.8643	0.5165	0.04907	3	3	14.88	12
Males :Iron Adequate (IA) vs. Females:Iron Adequate (IA)	1.381	1.012	0.3693	0.04907	3	3	10.64	12
Males :Iron Adequate (IA) vs. Females:Iron Deficient (ID)	1.381	0.8799	0.5009	0.04907	3	3	14.44	12
Males :Iron Adequate (IA) vs. Females:Iron Repletion (IR)	1.381	0.8655	0.5152	0.04907	3	3	14.85	12
Males :Iron Deficient (ID) vs. Males :Iron Repletion (IR)	1.127	0.8643	0.2624	0.04907	3	3	7.562	12
Males :Iron Deficient (ID) vs. Females:Iron Adequate (IA)	1.127	1.012	0.1152	0.04907	3	3	3.32	12
Males :Iron Deficient (ID) vs. Females:Iron Deficient (ID)	1.127	0.8799	0.2468	0.04907	3	3	7.114	12
Males :Iron Deficient (ID) vs. Females:Iron Repletion (IR)	1.127	0.8655	0.2612	0.04907	3	3	7.527	12
Males :Iron Repletion (IR) vs. Females:Iron Adequate (IA)	0.8643	1.012	-0.1472	0.04907	3	3	4.242	12
Males :Iron Repletion (IR) vs. Females:Iron Deficient (ID)	0.8643	0.8799	-0.01557	0.04907	3	3	0.4486	12
Males :Iron Repletion (IR) vs. Females:Iron Repletion (IR)	0.8643	0.8655	-0.001233	0.04907	3	3	0.03554	12
Females:Iron Adequate (IA) vs. Females:Iron Deficient (ID	1.012	0.8799	0.1316	0.04907	3	3	3.794	12
Females:Iron Adequate (IA) vs. Females:Iron Repletion (II	1.012	0.8655	0.146	0.04907	3	3	4.207	12
Females:Iron Deficient (ID) vs. Females:Iron Repletion (IR	0.8799	0.8655	0.01433	0.04907	3	3	0.4131	12

Figure 5B

Table Analyzed	**Figure 5B** FTH1 WB Veh x diet							
Two-way ANOVA	Ordinary							
Alpha	0.05							
Source of Variation	% of total variation	P value	P value sum	Significant?				
Interaction	3.358	0.3108	ns	No				
Sex	77.88	<0.0001	****	Yes				
Diet	3.15	0.332	ns	No				
ANOVA table	SS	DF	MS	F (DFn, DFd)	P value			
Interaction	0.03876	2	0.01938	F (2, 12) = 1.290	P=0.3108			
Sex	0.8988	1	0.8988	F (1, 12) = 59.84	P<0.0001			
Diet	0.03636	2	0.01818	F (2, 12) = 1.210	P=0.3320			
Residual	0.1802	12	0.01502					
Difference between row means								
Mean of Males	0.634							
Mean of Females	1.081							
Difference between means	-0.4469							
SE of difference	0.05777							
95% CI of difference	-0.5728 to -0.3210							
Data summary								
Number of columns (Diet)	3							
Number of rows (Sex)	2							
Number of values	18							
Number of families	1							
Number of comparisons per family	15							
Alpha	0.05							
Tukey's multiple comparisons test	Mean Diff.	95.00% CI of diff.	Below thres	Summary	Adjusted P Value			
Males:Iron Adequate (IA) vs. Males:Iron Deficient (ID)	-0.0838	-0.4199 to 0.2523	No	ns	0.9543			
Males:Iron Adequate (IA) vs. Males:Iron Repletion (IR)	-0.2172	-0.5533 to 0.1189	No	ns	0.3172			
Males:Iron Adequate (IA) vs. Females:Iron Adequate (IA)	-0.5329	-0.8690 to -0.1968	Yes	**	0.0019			
Males:Iron Adequate (IA) vs. Females:Iron Deficient (ID)	-0.5736	-0.9097 to -0.2375	Yes	**	0.001			
Males:Iron Adequate (IA) vs. Females:Iron Repletion (IR)	-0.5353	-0.8714 to -0.1992	Yes	**	0.0019			
Males:Iron Deficient (ID) vs. Males:Iron Repletion (IR)	-0.1334	-0.4695 to 0.2027	No	ns	0.7627			
Males:Iron Deficient (ID) vs. Females:Iron Adequate (IA)	-0.4491	-0.7852 to -0.1130	Yes	**	0.0075			
Males:Iron Deficient (ID) vs. Females:Iron Deficient (ID)	-0.4898	-0.8259 to -0.1537	Yes	**	0.0038			
Males:Iron Deficient (ID) vs. Females:Iron Repletion (IR)	-0.4515	-0.7876 to -0.1154	Yes	**	0.0072			
Males:Iron Repletion (IR) vs. Females:Iron Adequate (IA)	-0.3157	-0.6518 to 0.02038	No	ns	0.0702			
Males:Iron Repletion (IR) vs. Females:Iron Deficient (ID)	-0.3564	-0.6925 to -0.02025	Yes	*	0.0356			
Males:Iron Repletion (IR) vs. Females:Iron Repletion (IR)	-0.3181	-0.6542 to 0.01805	No	ns	0.0675			
Females:Iron Adequate (IA) vs. Females:Iron Deficient (ID)	-0.04063	-0.3767 to 0.2955	No	ns	0.9982			
Females:Iron Adequate (IA) vs. Females:Iron Repletion (IR)	-0.002333	-0.3384 to 0.3338	No	ns	>0.9999			
Females:Iron Deficient (ID) vs. Females:Iron Repletion (IR)	0.0383	-0.2978 to 0.3744	No	ns	0.9986			
Test details	Mean 1	Mean 2	Mean Diff.	SE of diff.	N1	N2	q	DF
Males:Iron Adequate (IA) vs. Males:Iron Deficient (ID)	0.5337	0.6175	-0.0838	0.1001	3	3	1.184	12
Males:Iron Adequate (IA) vs. Males:Iron Repletion (IR)	0.5337	0.7509	-0.2172	0.1001	3	3	3.07	12
Males:Iron Adequate (IA) vs. Females:Iron Adequate (IA)	0.5337	1.067	-0.5329	0.1001	3	3	7.532	12
Males:Iron Adequate (IA) vs. Females:Iron Deficient (ID)	0.5337	1.107	-0.5736	0.1001	3	3	8.106	12
Males:Iron Adequate (IA) vs. Females:Iron Repletion (IR)	0.5337	1.069	-0.5353	0.1001	3	3	7.565	12
Males:Iron Deficient (ID) vs. Males:Iron Repletion (IR)	0.6175	0.7509	-0.1334	0.1001	3	3	1.885	12
Males:Iron Deficient (ID) vs. Females:Iron Adequate (IA)	0.6175	1.067	-0.4491	0.1001	3	3	6.348	12
Males:Iron Deficient (ID) vs. Females:Iron Deficient (ID)	0.6175	1.107	-0.4898	0.1001	3	3	6.922	12
Males:Iron Deficient (ID) vs. Females:Iron Repletion (IR)	0.6175	1.069	-0.4515	0.1001	3	3	6.381	12
Males:Iron Repletion (IR) vs. Females:Iron Adequate (IA)	0.7509	1.067	-0.3157	0.1001	3	3	4.462	12
Males:Iron Repletion (IR) vs. Females:Iron Deficient (ID)	0.7509	1.107	-0.3564	0.1001	3	3	5.036	12
Males:Iron Repletion (IR) vs. Females:Iron Repletion (IR)	0.7509	1.069	-0.3181	0.1001	3	3	4.495	12
Females:Iron Adequate (IA) vs. Females:Iron Deficient (ID)	1.067	1.107	-0.04063	0.1001	3	3	0.5743	12
Females:Iron Adequate (IA) vs. Females:Iron Repletion (IR)	1.067	1.069	-0.002333	0.1001	3	3	0.03298	12
Females:Iron Deficient (ID) vs. Females:Iron Repletion (IR)	1.107	1.069	0.0383	0.1001	3	3	0.5413	12

Figure 5C

Table Analyzed	**Figure 5C** FTH1 WB LD x diet				
Two-way ANOVA	Ordinary				
Alpha	0.05				
Source of Variation	% of total variation	P value	P value sun	Significant?	
Interaction	31.5	0.0085	**	Yes	
Sex	6.228	0.1157	ns	No	
Diet	36.29	0.0053	**	Yes	
ANOVA table	SS	DF	MS	F (DFn, DFd)	P value
Interaction	0.2306	2	0.1153	F (2, 12) = 7.274	P=0.0085
Sex	0.04558	1	0.04558	F (1, 12) = 2.876	P=0.1157
Diet	0.2656	2	0.1328	F (2, 12) = 8.379	P=0.0053
Residual	0.1902	12	0.01585		
Difference between row means					
Mean of Males	1.011				
Mean of Females	1.111				
Difference between means	-0.1006				
SE of difference	0.05934				
95% CI of difference	-0.2299 to 0.02865				
Data summary					
Number of columns (Diet)	3				
Number of rows (Sex)	2				
Number of values	18				
Number of families	1				
Number of comparisons per family	15				
Alpha	0.05				

Tukey's multiple comparisons test	Mean Diff.	95.00% CI of diff.	Below thres	Summary	Adjusted P Value
Males:Iron Adequate (IA) vs. Males:Iron Deficient (ID)	0.07283	-0.2724 to 0.4181	No	ns	0.9772
Males:Iron Adequate (IA) vs. Males:Iron Repletion (IR)	0.4379	0.09262 to 0.7831	Yes	*	0.0109
Males:Iron Adequate (IA) vs. Females:Iron Adequate (IA)	-0.08837	-0.4336 to 0.2569	No	ns	0.9493
Males:Iron Adequate (IA) vs. Females:Iron Deficient (ID)	0.2431	-0.1022 to 0.5883	No	ns	0.242
Males:Iron Adequate (IA) vs. Females:Iron Repletion (IR)	0.05407	-0.2912 to 0.3993	No	ns	0.9939
Males:Iron Deficient (ID) vs. Males:Iron Repletion (IR)	0.365	0.01978 to 0.7103	Yes	*	0.0362
Males:Iron Deficient (ID) vs. Females:Iron Adequate (IA)	-0.1612	-0.5064 to 0.1840	No	ns	0.6317
Males:Iron Deficient (ID) vs. Females:Iron Deficient (ID)	0.1702	-0.1750 to 0.5155	No	ns	0.5812
Males:Iron Deficient (ID) vs. Females:Iron Repletion (IR)	-0.01877	-0.3640 to 0.3265	No	ns	>0.9999
Males:Iron Repletion (IR) vs. Females:Iron Adequate (IA)	-0.5262	-0.8715 to -0.1810	Yes	**	0.0027
Males:Iron Repletion (IR) vs. Females:Iron Deficient (ID)	-0.1948	-0.5400 to 0.1504	No	ns	0.4488
Males:Iron Repletion (IR) vs. Females:Iron Repletion (IR)	-0.3838	-0.7290 to -0.0385	Yes	*	0.0266
Females:Iron Adequate (IA) vs. Females:Iron Deficient (ID)	0.3314	-0.01382 to 0.6767	No	ns	0.0626
Females:Iron Adequate (IA) vs. Females:Iron Repletion (IR)	0.1424	-0.2028 to 0.4877	No	ns	0.7345
Females:Iron Deficient (ID) vs. Females:Iron Repletion (IR)	-0.189	-0.5342 to 0.1562	No	ns	0.4789

Test details	Mean 1	Mean 2	Mean Diff.	SE of diff.	N1	N2	q	DF
Males:Iron Adequate (IA) vs. Males:Iron Deficient (ID)	1.181	1.108	0.07283	0.1028	3	3	1.002	12
Males:Iron Adequate (IA) vs. Males:Iron Repletion (IR)	1.181	0.7431	0.4379	0.1028	3	3	6.025	12
Males:Iron Adequate (IA) vs. Females:Iron Adequate (IA)	1.181	1.269	-0.08837	0.1028	3	3	1.216	12
Males:Iron Adequate (IA) vs. Females:Iron Deficient (ID)	1.181	0.9379	0.2431	0.1028	3	3	3.344	12
Males:Iron Adequate (IA) vs. Females:Iron Repletion (IR)	1.181	1.127	0.05407	0.1028	3	3	0.7439	12
Males:Iron Deficient (ID) vs. Males:Iron Repletion (IR)	1.108	0.7431	0.365	0.1028	3	3	5.022	12
Males:Iron Deficient (ID) vs. Females:Iron Adequate (IA)	1.108	1.269	-0.1612	0.1028	3	3	2.218	12
Males:Iron Deficient (ID) vs. Females:Iron Deficient (ID)	1.108	0.9379	0.1702	0.1028	3	3	2.342	12
Males:Iron Deficient (ID) vs. Females:Iron Repletion (IR)	1.108	1.127	-0.01877	0.1028	3	3	0.2582	12
Males:Iron Repletion (IR) vs. Females:Iron Adequate (IA)	0.7431	1.269	-0.5262	0.1028	3	3	7.24	12
Males:Iron Repletion (IR) vs. Females:Iron Deficient (ID)	0.7431	0.9379	-0.1948	0.1028	3	3	2.68	12
Males:Iron Repletion (IR) vs. Females:Iron Repletion (IR)	0.7431	1.127	-0.3838	0.1028	3	3	5.281	12
Females:Iron Adequate (IA) vs. Females:Iron Deficient (ID)	1.269	0.9379	0.3314	0.1028	3	3	4.56	12
Females:Iron Adequate (IA) vs. Females:Iron Repletion (IR)	1.269	1.127	0.1424	0.1028	3	3	1.96	12
Females:Iron Deficient (ID) vs. Females:Iron Repletion (IR)	0.9379	1.127	-0.189	0.1028	3	3	2.6	12

Figure 5D

Table Analyzed	**Figure 5D** FTH1 WB Sel x diet							
Two-way ANOVA	Ordinary							
Alpha	0.05							
Source of Variation	% of total variation	P value	P value sun	Significant?				
Interaction	14.12	0.1535	ns	No				
Sex	26.3	0.0143	*	Yes				
Diet	21.08	0.0728	ns	No				
ANOVA table	SS	DF	MS	F (DFn, DFd)	P value			
Interaction	0.1331	2	0.06653	F (2, 12) = 2.200	P=0.1535			
Sex	0.2479	1	0.2479	F (1, 12) = 8.198	P=0.0143			
Diet	0.1987	2	0.09933	F (2, 12) = 3.285	P=0.0728			
Residual	0.3629	12	0.03024					
Difference between row means								
Mean of Males	0.9071							
Mean of Females	1.142							
Difference between means	-0.2347							
SE of difference	0.08198							
95% CI of difference	-0.4133 to -0.05611							
Data summary								
Number of columns (Diet)	3							
Number of rows (Sex)	2							
Number of values	18							
Number of families	1							
Number of comparisons per family	15							
Alpha	0.05							
Tukey's multiple comparisons test	Mean Diff.	95.00% CI of diff.	Below thres	Summary	Adjusted P	Value		
Males:Iron Adequate (IA) vs. Males:Iron Deficient (ID)	0.1964	-0.2805 to 0.6733	No	ns	0.7359			
Males:Iron Adequate (IA) vs. Males:Iron Repletion (IR)	-0.05467	-0.5316 to 0.4223	No	ns	0.9986			
Males:Iron Adequate (IA) vs. Females:Iron Adequate (IA)	-0.411	-0.8879 to 0.06592	No	ns	0.1074			
Males:Iron Adequate (IA) vs. Females:Iron Deficient (ID)	-0.09527	-0.5722 to 0.3817	No	ns	0.982			
Males:Iron Adequate (IA) vs. Females:Iron Repletion (IR)	-0.05617	-0.5331 to 0.4208	No	ns	0.9984			
Males:Iron Deficient (ID) vs. Males:Iron Repletion (IR)	-0.2511	-0.7280 to 0.2259	No	ns	0.5177			
Males:Iron Deficient (ID) vs. Females:Iron Adequate (IA)	-0.6074	-1.084 to -0.1305	Yes	*	0.0106			
Males:Iron Deficient (ID) vs. Females:Iron Deficient (ID)	-0.2917	-0.7686 to 0.1853	No	ns	0.3694			
Males:Iron Deficient (ID) vs. Females:Iron Repletion (IR)	-0.2526	-0.7295 to 0.2244	No	ns	0.5118			
Males:Iron Repletion (IR) vs. Females:Iron Adequate (IA)	-0.3563	-0.8333 to 0.1206	No	ns	0.1956			
Males:Iron Repletion (IR) vs. Females:Iron Deficient (ID)	-0.0406	-0.5175 to 0.4363	No	ns	0.9997			
Males:Iron Repletion (IR) vs. Females:Iron Repletion (IR)	-0.0015	-0.4784 to 0.4754	No	ns	>0.9999			
Females:Iron Adequate (IA) vs. Females:Iron Deficient (ID)	0.3157	-0.1612 to 0.7927	No	ns	0.2951			
Females:Iron Adequate (IA) vs. Females:Iron Repletion (IR)	0.3548	-0.1221 to 0.8318	No	ns	0.1988			
Females:Iron Deficient (ID) vs. Females:Iron Repletion (IR)	0.0391	-0.4378 to 0.5160	No	ns	0.9997			
Test details	Mean 1	Mean 2	Mean Diff.	SE of diff.	N1	N2	q	DF
Males:Iron Adequate (IA) vs. Males:Iron Deficient (ID)	0.9543	0.7579	0.1964	0.142	3	3	1.956	12
Males:Iron Adequate (IA) vs. Males:Iron Repletion (IR)	0.9543	1.009	-0.05467	0.142	3	3	0.5445	12
Males:Iron Adequate (IA) vs. Females:Iron Adequate (IA)	0.9543	1.365	-0.411	0.142	3	3	4.094	12
Males:Iron Adequate (IA) vs. Females:Iron Deficient (ID)	0.9543	1.05	-0.09527	0.142	3	3	0.9489	12
Males:Iron Adequate (IA) vs. Females:Iron Repletion (IR)	0.9543	1.01	-0.05617	0.142	3	3	0.5594	12
Males:Iron Deficient (ID) vs. Males:Iron Repletion (IR)	0.7579	1.009	-0.2511	0.142	3	3	2.501	12
Males:Iron Deficient (ID) vs. Females:Iron Adequate (IA)	0.7579	1.365	-0.6074	0.142	3	3	6.05	12
Males:Iron Deficient (ID) vs. Females:Iron Deficient (ID)	0.7579	1.05	-0.2917	0.142	3	3	2.905	12
Males:Iron Deficient (ID) vs. Females:Iron Repletion (IR)	0.7579	1.01	-0.2526	0.142	3	3	2.516	12
Males:Iron Repletion (IR) vs. Females:Iron Adequate (IA)	1.009	1.365	-0.3563	0.142	3	3	3.549	12
Males:Iron Repletion (IR) vs. Females:Iron Deficient (ID)	1.009	1.05	-0.0406	0.142	3	3	0.4044	12
Males:Iron Repletion (IR) vs. Females:Iron Repletion (IR)	1.009	1.01	-0.0015	0.142	3	3	0.01494	12
Females:Iron Adequate (IA) vs. Females:Iron Deficient (ID)	1.365	1.05	0.3157	0.142	3	3	3.145	12
Females:Iron Adequate (IA) vs. Females:Iron Repletion (IR)	1.365	1.01	0.3548	0.142	3	3	3.534	12
Females:Iron Deficient (ID) vs. Females:Iron Repletion (IR)	1.05	1.01	0.0391	0.142	3	3	0.3894	12

Figure 5E

Table Analyzed	**Figure 5E** FTL WB Veh x diet							
Two-way ANOVA	Ordinary							
Alpha	0.05							
Source of Variation	% of total variation	P value	P value summar	Significant?				
Interaction	19.13	0.0028	**	Yes				
Sex	2.32	0.1461	ns	No				
Diet	67.02	<0.0001	****	Yes				
ANOVA table	SS	DF	MS	F (DFn, DFd)	P value			
Interaction	0.2217	2	0.1108	F (2, 12) = 9.953	P=0.0028			
Sex	0.02689	1	0.02689	F (1, 12) = 2.415	P=0.1461			
Diet	0.7768	2	0.3884	F (2, 12) = 34.88	P<0.0001			
Residual	0.1336	12	0.01114					
Difference between row means								
Mean of Males	1.018							
Mean of Females	0.9409							
Difference between means	0.0773							
SE of difference	0.04975							
95% CI of difference	-0.03108 to 0.1857							
Data summary								
Number of columns (Diet)	3							
Number of rows (Sex)	2							
Number of values	18							
Number of families	1							
Number of comparisons per family	15							
Alpha	0.05							
Tukey's multiple comparisons test	Mean Diff.	95.00% CI of diff.	Below threshold	Summary	Adjusted P Value			
Males:Iron Adequate (IA) vs. Males:Iron Deficient (ID)	0.6229	0.3335 to 0.9123	Yes	***	0.0001			
Males:Iron Adequate (IA) vs. Males:Iron Repletion (IR)	0.5831	0.2937 to 0.8725	Yes	***	0.0002			
Males:Iron Adequate (IA) vs. Females:Iron Adequate (IA)	0.3372	0.04779 to 0.6266	Yes	*	0.0196			
Males:Iron Adequate (IA) vs. Females:Iron Deficient (ID)	0.7226	0.4332 to 1.012	Yes	****	<0.0001			
Males:Iron Adequate (IA) vs. Females:Iron Repletion (IR)	0.378	0.08861 to 0.6674	Yes	**	0.0088			
Males:Iron Deficient (ID) vs. Males:Iron Repletion (IR)	-0.03979	-0.3292 to 0.2496	No	ns	0.9967			
Males:Iron Deficient (ID) vs. Females:Iron Adequate (IA)	-0.2857	-0.5751 to 0.003753	No	ns	0.0538			
Males:Iron Deficient (ID) vs. Females:Iron Deficient (ID)	0.09976	-0.1896 to 0.3892	No	ns	0.8479			
Males:Iron Deficient (ID) vs. Females:Iron Repletion (IR)	-0.2448	-0.5343 to 0.04457	No	ns	0.1169			
Males:Iron Repletion (IR) vs. Females:Iron Adequate (IA)	-0.2459	-0.5353 to 0.04354	No	ns	0.1147			
Males:Iron Repletion (IR) vs. Females:Iron Deficient (ID)	0.1396	-0.1499 to 0.4290	No	ns	0.6021			
Males:Iron Repletion (IR) vs. Females:Iron Repletion (IR)	-0.2051	-0.4945 to 0.08436	No	ns	0.2368			
Females:Iron Adequate (IA) vs. Females:Iron Deficient (ID)	0.3854	0.09601 to 0.6748	Yes	**	0.0077			
Females:Iron Adequate (IA) vs. Females:Iron Repletion (IR)	0.04081	-0.2486 to 0.3302	No	ns	0.9963			
Females:Iron Deficient (ID) vs. Females:Iron Repletion (IR)	-0.3446	-0.6340 to -0.05520	Yes	*	0.017			
Test details	Mean 1	Mean 2	Mean Diff.	SE of diff.	N1	N2	q	DF
Males:Iron Adequate (IA) vs. Males:Iron Deficient (ID)	1.42	0.7973	0.6229	0.08616	3	3	10.22	12
Males:Iron Adequate (IA) vs. Males:Iron Repletion (IR)	1.42	0.8371	0.5831	0.08616	3	3	9.57	12
Males:Iron Adequate (IA) vs. Females:Iron Adequate (IA)	1.42	1.083	0.3372	0.08616	3	3	5.535	12
Males:Iron Adequate (IA) vs. Females:Iron Deficient (ID)	1.42	0.6976	0.7226	0.08616	3	3	11.86	12
Males:Iron Adequate (IA) vs. Females:Iron Repletion (IR)	1.42	1.042	0.378	0.08616	3	3	6.205	12
Males:Iron Deficient (ID) vs. Males:Iron Repletion (IR)	0.7973	0.8371	-0.03979	0.08616	3	3	0.6531	12
Males:Iron Deficient (ID) vs. Females:Iron Adequate (IA)	0.7973	1.083	-0.2857	0.08616	3	3	4.689	12
Males:Iron Deficient (ID) vs. Females:Iron Deficient (ID)	0.7973	0.6976	0.09976	0.08616	3	3	1.637	12
Males:Iron Deficient (ID) vs. Females:Iron Repletion (IR)	0.7973	1.042	-0.2448	0.08616	3	3	4.019	12
Males:Iron Repletion (IR) vs. Females:Iron Adequate (IA)	0.8371	1.083	-0.2459	0.08616	3	3	4.036	12
Males:Iron Repletion (IR) vs. Females:Iron Deficient (ID)	0.8371	0.6976	0.1396	0.08616	3	3	2.291	12
Males:Iron Repletion (IR) vs. Females:Iron Repletion (IR)	0.8371	1.042	-0.2051	0.08616	3	3	3.366	12
Females:Iron Adequate (IA) vs. Females:Iron Deficient (ID)	1.083	0.6976	0.3854	0.08616	3	3	6.326	12
Females:Iron Adequate (IA) vs. Females:Iron Repletion (IR)	1.083	1.042	0.04081	0.08616	3	3	0.6699	12
Females:Iron Deficient (ID) vs. Females:Iron Repletion (IR)	0.6976	1.042	-0.3446	0.08616	3	3	5.656	12

Figure 5F

Table Analyzed	**Figure 5F** FTL WB LD x diet							
Two-way ANOVA	Ordinary							
Alpha	0.05							
Source of Variation	% of total variation	P value	P value sun	Significant?				
Interaction	5.24	0.3282	ns	No				
Sex	0.9372	0.5206	ns	No				
Diet	68.15	0.0004	***	Yes				
ANOVA table	SS	DF	MS	F (DFn, DFd)	P value			
Interaction	0.07019	2	0.0351	F (2, 12) = 1.224	P=0.3282			
Sex	0.01255	1	0.01255	F (1, 12) = 0.4380	P=0.5206			
Diet	0.9128	2	0.4564	F (2, 12) = 15.92	P=0.0004			
Residual	0.344	12	0.02866					
Difference between row means								
Mean of Males	0.9621							
Mean of Females	0.9093							
Difference between means	0.05282							
SE of difference	0.07981							
95% CI of difference	-0.1211 to 0.2267							
Data summary								
Number of columns (Diet)	3							
Number of rows (Sex)	2							
Number of values	18							
Number of families	1							
Number of comparisons per family	15							
Alpha	0.05							
Tukey's multiple comparisons test	Mean Diff.	95.00% CI of diff.	Below thres	Summary	Adjusted P Value			
Males:Iron Adequate (IA) vs. Males:Iron Deficient (ID)	0.3828	-0.08157 to 0.8471	No	ns	0.1312			
Males:Iron Adequate (IA) vs. Males:Iron Repletion (IR)	0.01688	-0.4474 to 0.4812	No	ns	>0.9999			
Males:Iron Adequate (IA) vs. Females:Iron Adequate (IA)	-0.1194	-0.5837 to 0.3450	No	ns	0.9484			
Males:Iron Adequate (IA) vs. Females:Iron Deficient (ID)	0.5558	0.09146 to 1.020	Yes	*	0.0164			
Males:Iron Adequate (IA) vs. Females:Iron Repletion (IR)	0.1217	-0.3427 to 0.5860	No	ns	0.9443			
Males:Iron Deficient (ID) vs. Males:Iron Repletion (IR)	-0.3659	-0.8302 to 0.09845	No	ns	0.1588			
Males:Iron Deficient (ID) vs. Females:Iron Adequate (IA)	-0.5021	-0.9664 to -0.03779	Yes	*	0.0316			
Males:Iron Deficient (ID) vs. Females:Iron Deficient (ID)	0.173	-0.2913 to 0.6374	No	ns	0.8042			
Males:Iron Deficient (ID) vs. Females:Iron Repletion (IR)	-0.2611	-0.7254 to 0.2032	No	ns	0.4522			
Males:Iron Repletion (IR) vs. Females:Iron Adequate (IA)	-0.1362	-0.6006 to 0.3281	No	ns	0.914			
Males:Iron Repletion (IR) vs. Females:Iron Deficient (ID)	0.5389	0.07458 to 1.003	Yes	*	0.0201			
Males:Iron Repletion (IR) vs. Females:Iron Repletion (IR)	0.1048	-0.3595 to 0.5691	No	ns	0.9697			
Females:Iron Adequate (IA) vs. Females:Iron Deficient (ID)	0.6751	0.2108 to 1.139	Yes	**	0.0039			
Females:Iron Adequate (IA) vs. Females:Iron Repletion (IR)	0.241	-0.2233 to 0.7053	No	ns	0.5315			
Females:Iron Deficient (ID) vs. Females:Iron Repletion (IR)	-0.4341	-0.8984 to 0.03020	No	ns	0.0719			
Test details	Mean 1	Mean 2	Mean Diff.	SE of diff.	N1	N2	q	DF
Males:Iron Adequate (IA) vs. Males:Iron Deficient (ID)	1.095	0.7125	0.3828	0.1382	3	3	3.916	12
Males:Iron Adequate (IA) vs. Males:Iron Repletion (IR)	1.095	1.078	0.01688	0.1382	3	3	0.1727	12
Males:Iron Adequate (IA) vs. Females:Iron Adequate (IA)	1.095	1.215	-0.1194	0.1382	3	3	1.221	12
Males:Iron Adequate (IA) vs. Females:Iron Deficient (ID)	1.095	0.5395	0.5558	0.1382	3	3	5.686	12
Males:Iron Adequate (IA) vs. Females:Iron Repletion (IR)	1.095	0.9736	0.1217	0.1382	3	3	1.245	12
Males:Iron Deficient (ID) vs. Males:Iron Repletion (IR)	0.7125	1.078	-0.3659	0.1382	3	3	3.743	12
Males:Iron Deficient (ID) vs. Females:Iron Adequate (IA)	0.7125	1.215	-0.5021	0.1382	3	3	5.137	12
Males:Iron Deficient (ID) vs. Females:Iron Deficient (ID)	0.7125	0.5395	0.173	0.1382	3	3	1.77	12
Males:Iron Deficient (ID) vs. Females:Iron Repletion (IR)	0.7125	0.9736	-0.2611	0.1382	3	3	2.671	12
Males:Iron Repletion (IR) vs. Females:Iron Adequate (IA)	1.078	1.215	-0.1362	0.1382	3	3	1.394	12
Males:Iron Repletion (IR) vs. Females:Iron Deficient (ID)	1.078	0.5395	0.5389	0.1382	3	3	5.513	12
Males:Iron Repletion (IR) vs. Females:Iron Repletion (IR)	1.078	0.9736	0.1048	0.1382	3	3	1.072	12
Females:Iron Adequate (IA) vs. Females:Iron Deficient (ID)	1.215	0.5395	0.6751	0.1382	3	3	6.907	12
Females:Iron Adequate (IA) vs. Females:Iron Repletion (IR)	1.215	0.9736	0.241	0.1382	3	3	2.466	12
Females:Iron Deficient (ID) vs. Females:Iron Repletion (IR)	0.5395	0.9736	-0.4341	0.1382	3	3	4.441	12

Figure 5G

Table Analyzed	**Figure 5G** FTL WB Sel x diet							
Two-way ANOVA	Ordinary							
Alpha	0.05							
Source of Variation	% of total variation	P value	P value summary	Significant?				
Interaction	10.33	0.1256	ns	No				
Sex	0.4005	0.6689	ns	No				
Diet	64.25	0.0005	***	Yes				
ANOVA table	SS	DF	MS	F (DFn, DFd)	P value			
Interaction	0.1386	2	0.06932	F (2, 12) = 2.479	P=0.1256			
Sex	0.005374	1	0.005374	F (1, 12) = 0.1922	P=0.6689			
Diet	0.8621	2	0.4311	F (2, 12) = 15.41	P=0.0005			
Residual	0.3356	12	0.02797					
Difference between row means								
Mean of Males	0.9062							
Mean of Females	0.8717							
Difference between means	0.03456							
SE of difference	0.07883							
95% CI of difference	-0.1372 to 0.2063							
Data summary								
Number of columns (Diet)	3							
Number of rows (Sex)	2							
Number of values	18							
Number of families	1							
Number of comparisons per family	15							
Alpha	0.05							
Tukey's multiple comparisons test	Mean Diff.	95.00% CI of diff.	Below threshold?	Summary	Adjusted P Value			
Males:Iron Adequate (IA) vs. Males:Iron Deficient (ID)	0.5881	0.1295 to 1.047	Yes	*	0.0101			
Males:Iron Adequate (IA) vs. Males:Iron Repletion (IR)	0.6383	0.1797 to 1.097	Yes	**	0.0055			
Males:Iron Adequate (IA) vs. Females:Iron Adequate (IA)	0.2388	-0.2198 to 0.6974	No	ns	0.5285			
Males:Iron Adequate (IA) vs. Females:Iron Deficient (ID)	0.6428	0.1841 to 1.101	Yes	**	0.0052			
Males:Iron Adequate (IA) vs. Females:Iron Repletion (IR)	0.4486	-0.01005 to 0.9072	No	ns	0.0565			
Males:Iron Deficient (ID) vs. Males:Iron Repletion (IR)	0.05021	-0.4084 to 0.5088	No	ns	0.9989			
Males:Iron Deficient (ID) vs. Females:Iron Adequate (IA)	-0.3493	-0.8080 to 0.1093	No	ns	0.1818			
Males:Iron Deficient (ID) vs. Females:Iron Deficient (ID)	0.05462	-0.4040 to 0.5133	No	ns	0.9983			
Males:Iron Deficient (ID) vs. Females:Iron Repletion (IR)	-0.1395	-0.5982 to 0.3191	No	ns	0.9017			
Males:Iron Repletion (IR) vs. Females:Iron Adequate (IA)	-0.3995	-0.8582 to 0.05909	No	ns	0.1021			
Males:Iron Repletion (IR) vs. Females:Iron Deficient (ID)	0.004413	-0.4542 to 0.4630	No	ns	>0.9999			
Males:Iron Repletion (IR) vs. Females:Iron Repletion (IR)	-0.1898	-0.6484 to 0.2689	No	ns	0.7324			
Females:Iron Adequate (IA) vs. Females:Iron Deficient (ID)	0.404	-0.05468 to 0.8626	No	ns	0.0969			
Females:Iron Adequate (IA) vs. Females:Iron Repletion (IR)	0.2098	-0.2488 to 0.6684	No	ns	0.65			
Females:Iron Deficient (ID) vs. Females:Iron Repletion (IR)	-0.1942	-0.6528 to 0.2645	No	ns	0.7146			
Test details	Mean 1	Mean 2	Mean Diff.	SE of diff.	N1	N2	q	DF
Males:Iron Adequate (IA) vs. Males:Iron Deficient (ID)	1.315	0.7269	0.5881	0.1365	3	3	6.091	12
Males:Iron Adequate (IA) vs. Males:Iron Repletion (IR)	1.315	0.6767	0.6383	0.1365	3	3	6.612	12
Males:Iron Adequate (IA) vs. Females:Iron Adequate (IA)	1.315	1.076	0.2388	0.1365	3	3	2.473	12
Males:Iron Adequate (IA) vs. Females:Iron Deficient (ID)	1.315	0.6723	0.6428	0.1365	3	3	6.657	12
Males:Iron Adequate (IA) vs. Females:Iron Repletion (IR)	1.315	0.8665	0.4486	0.1365	3	3	4.646	12
Males:Iron Deficient (ID) vs. Males:Iron Repletion (IR)	0.7269	0.6767	0.05021	0.1365	3	3	0.52	12
Males:Iron Deficient (ID) vs. Females:Iron Adequate (IA)	0.7269	1.076	-0.3493	0.1365	3	3	3.618	12
Males:Iron Deficient (ID) vs. Females:Iron Deficient (ID)	0.7269	0.6723	0.05462	0.1365	3	3	0.5658	12
Males:Iron Deficient (ID) vs. Females:Iron Repletion (IR)	0.7269	0.8665	-0.1395	0.1365	3	3	1.445	12
Males:Iron Repletion (IR) vs. Females:Iron Adequate (IA)	0.6767	1.076	-0.3995	0.1365	3	3	4.138	12
Males:Iron Repletion (IR) vs. Females:Iron Deficient (ID)	0.6767	0.6723	0.004413	0.1365	3	3	0.04571	12
Males:Iron Repletion (IR) vs. Females:Iron Repletion (IR)	0.6767	0.8665	-0.1898	0.1365	3	3	1.965	12
Females:Iron Adequate (IA) vs. Females:Iron Deficient (ID)	1.076	0.6723	0.404	0.1365	3	3	4.184	12
Females:Iron Adequate (IA) vs. Females:Iron Repletion (IR)	1.076	0.8665	0.2098	0.1365	3	3	2.173	12
Females:Iron Deficient (ID) vs. Females:Iron Repletion (IR)	0.6723	0.8665	-0.1942	0.1365	3	3	2.011	12

Figure 6B

Table Analyzed	**Figure 6B** LCN2 WB Veh x diet				
Two-way ANOVA	Ordinary				
Alpha	0.05				
Source of Variation	% of total variation	P value	P value sun	Significant?	
Interaction	8.924	0.3211	ns	No	
Sex	9.464	0.1293	ns	No	
Diet	38.8	0.0208	*	Yes	
ANOVA table	SS	DF	MS	F (DFn, DFd)	P value
Interaction	0.03412	2	0.01706	F (2, 12) = 1.251	P=0.3211
Sex	0.03619	1	0.03619	F (1, 12) = 2.653	P=0.1293
Diet	0.1484	2	0.07418	F (2, 12) = 5.437	P=0.0208
Residual	0.1637	12	0.01364		
Difference between row means					
Mean of Males	0.6701				
Mean of Females	0.5804				
Difference between means	0.08968				
SE of difference	0.05506				
95% CI of difference	-0.03029 to 0.2096				
Data summary					
Number of columns (Diet)	3				
Number of rows (Sex)	2				
Number of values	18				
Number of families	1				
Number of comparisons per family	15				
Alpha	0.05				

Tukey's multiple comparisons test	Mean Diff.	95.00% CI of diff.	Below thres	Summary	Adjusted P Value
Males :Iron Adequate (IA) vs. Males :Iron Deficient (ID)	0.1369	-0.1834 to 0.4573	No	ns	0.7069
Males :Iron Adequate (IA) vs. Males :Iron Repletion (IR)	0.1121	-0.2082 to 0.4324	No	ns	0.8401
Males :Iron Adequate (IA) vs. Females:Iron Adequate (IA)	0.006567	-0.3138 to 0.3269	No	ns	>0.9999
Males :Iron Adequate (IA) vs. Females:Iron Deficient (ID)	0.1895	-0.1309 to 0.5098	No	ns	0.4021
Males :Iron Adequate (IA) vs. Females:Iron Repletion (IR)	0.322	0.001694 to 0.6424	Yes	*	0.0485
Males :Iron Deficient (ID) vs. Males :Iron Repletion (IR)	-0.02483	-0.3452 to 0.2955	No	ns	0.9998
Males :Iron Deficient (ID) vs. Females:Iron Adequate (IA)	-0.1304	-0.4507 to 0.1900	No	ns	0.7447
Males :Iron Deficient (ID) vs. Females:Iron Deficient (ID)	0.05253	-0.2678 to 0.3729	No	ns	0.9925
Males :Iron Deficient (ID) vs. Females:Iron Repletion (IR)	0.1851	-0.1352 to 0.5054	No	ns	0.4251
Males :Iron Repletion (IR) vs. Females:Iron Adequate (IA)	-0.1055	-0.4259 to 0.2148	No	ns	0.8696
Males :Iron Repletion (IR) vs. Females:Iron Deficient (ID)	0.07737	-0.2430 to 0.3977	No	ns	0.9599
Males :Iron Repletion (IR) vs. Females:Iron Repletion (IR)	0.2099	-0.1104 to 0.5303	No	ns	0.3043
Females:Iron Adequate (IA) vs. Females:Iron Deficient (ID)	0.1829	-0.1374 to 0.5032	No	ns	0.437
Females:Iron Adequate (IA) vs. Females:Iron Repletion (IR)	0.3155	-0.004872 to 0.6358	No	ns	0.0545
Females:Iron Deficient (ID) vs. Females:Iron Repletion (IR)	0.1326	-0.1878 to 0.4529	No	ns	0.7322

Test details	Mean 1	Mean 2	Mean Diff.	SE of diff.	N1	N2	q	DF
Males :Iron Adequate (IA) vs. Males :Iron Deficient (ID)	0.7531	0.6162	0.1369	0.09537	3	3	2.031	12
Males :Iron Adequate (IA) vs. Males :Iron Repletion (IR)	0.7531	0.641	0.1121	0.09537	3	3	1.662	12
Males :Iron Adequate (IA) vs. Females:Iron Adequate (IA)	0.7531	0.7466	0.006567	0.09537	3	3	0.09738	12
Males :Iron Adequate (IA) vs. Females:Iron Deficient (ID)	0.7531	0.5637	0.1895	0.09537	3	3	2.81	12
Males :Iron Adequate (IA) vs. Females:Iron Repletion (IR)	0.7531	0.4311	0.322	0.09537	3	3	4.775	12
Males :Iron Deficient (ID) vs. Males :Iron Repletion (IR)	0.6162	0.641	-0.02483	0.09537	3	3	0.3682	12
Males :Iron Deficient (ID) vs. Females:Iron Adequate (IA)	0.6162	0.7466	-0.1304	0.09537	3	3	1.933	12
Males :Iron Deficient (ID) vs. Females:Iron Deficient (ID)	0.6162	0.5637	0.05253	0.09537	3	3	0.779	12
Males :Iron Deficient (ID) vs. Females:Iron Repletion (IR)	0.6162	0.4311	0.1851	0.09537	3	3	2.745	12
Males :Iron Repletion (IR) vs. Females:Iron Adequate (IA)	0.641	0.7466	-0.1055	0.09537	3	3	1.565	12
Males :Iron Repletion (IR) vs. Females:Iron Deficient (ID)	0.641	0.5637	0.07737	0.09537	3	3	1.147	12
Males :Iron Repletion (IR) vs. Females:Iron Repletion (IR)	0.641	0.4311	0.2099	0.09537	3	3	3.113	12
Females:Iron Adequate (IA) vs. Females:Iron Deficient (ID)	0.7466	0.5637	0.1829	0.09537	3	3	2.712	12
Females:Iron Adequate (IA) vs. Females:Iron Repletion (IR)	0.7466	0.4311	0.3155	0.09537	3	3	4.678	12
Females:Iron Deficient (ID) vs. Females:Iron Repletion (IR)	0.5637	0.4311	0.1326	0.09537	3	3	1.966	12

Figure 6C

Table Analyzed	**Figure 6C** LCN2 WB LD x diet						
Two-way ANOVA	Ordinary						
Alpha	0.05						
Source of Variation	% of total variation	P value	P value sun	Significant?			
Interaction	38.68	0.0008	***	Yes			
Sex	16.25	0.0055	**	Yes			
Diets	28.01	0.0029	**	Yes			
ANOVA table	SS	DF	MS	F (DFn, DFd)	P value		
Interaction	0.3373	2	0.1687	F (2, 12) = 13.60	P=0.0008		
Sex	0.1417	1	0.1417	F (1, 12) = 11.43	P=0.0055		
Diets	0.2443	2	0.1221	F (2, 12) = 9.851	P=0.0029		
Residual	0.1488	12	0.0124				
Difference between row means							
Mean of Males	0.7817						
Mean of Females	0.6043						
Difference between means	0.1775						
SE of difference	0.05249						
95% CI of difference	0.06310 to 0.2918						
Data summary							
Number of columns (Diets)	3						
Number of rows (Sex)	2						
Number of values	18						
Number of families	1						
Number of comparisons per family	15						
Alpha	0.05						
Tukey's multiple comparisons test	Mean Diff.	95.00% CI of diff.	Below thres	Summary	Adjusted P Value		
Males :Iron Adequate (IA) vs. Males :Iron Deficient (ID)	-0.0286	-0.3340 to 0.2768	No	ns	0.9995		
Males :Iron Adequate (IA) vs. Males :Iron Repletion (IR)	-0.541	-0.8464 to -0.2356	Yes	***	0.0007		
Males :Iron Adequate (IA) vs. Females:Iron Adequate (IA)	0.03013	-0.2753 to 0.3355	No	ns	0.9993		
Males :Iron Adequate (IA) vs. Females:Iron Deficient (ID)	-0.08757	-0.3930 to 0.2178	No	ns	0.9211		
Males :Iron Adequate (IA) vs. Females:Iron Repletion (IR)	0.02023	-0.2852 to 0.3256	No	ns	>0.9999		
Males :Iron Deficient (ID) vs. Males :Iron Repletion (IR)	-0.5124	-0.8178 to -0.2070	Yes	**	0.0012		
Males :Iron Deficient (ID) vs. Females:Iron Adequate (IA)	0.05873	-0.2467 to 0.3641	No	ns	0.9848		
Males :Iron Deficient (ID) vs. Females:Iron Deficient (ID)	-0.05897	-0.3644 to 0.2464	No	ns	0.9845		
Males :Iron Deficient (ID) vs. Females:Iron Repletion (IR)	0.04883	-0.2566 to 0.3542	No	ns	0.9933		
Males :Iron Repletion (IR) vs. Females:Iron Adequate (IA)	0.5711	0.2657 to 0.8765	Yes	***	0.0004		
Males :Iron Repletion (IR) vs. Females:Iron Deficient (ID)	0.4534	0.1480 to 0.7588	Yes	**	0.0033		
Males :Iron Repletion (IR) vs. Females:Iron Repletion (IR)	0.5612	0.2558 to 0.8666	Yes	***	0.0005		
Females:Iron Adequate (IA) vs. Females:Iron Deficient (ID)	-0.1177	-0.4231 to 0.1877	No	ns	0.7827		
Females:Iron Adequate (IA) vs. Females:Iron Repletion (IR)	-0.0099	-0.3153 to 0.2955	No	ns	>0.9999		
Females:Iron Deficient (ID) vs. Females:Iron Repletion (IR)	0.1078	-0.1976 to 0.4132	No	ns	0.8354		

Test details	Mean 1	Mean 2	Mean Diff.	SE of diff.	N1	N2	q	DF
Males :Iron Adequate (IA) vs. Males :Iron Deficient (ID)	0.5919	0.6205	-0.0286	0.09092	3	3	0.4449	12
Males :Iron Adequate (IA) vs. Males :Iron Repletion (IR)	0.5919	1.133	-0.541	0.09092	3	3	8.415	12
Males :Iron Adequate (IA) vs. Females:Iron Adequate (IA)	0.5919	0.5617	0.03013	0.09092	3	3	0.4687	12
Males :Iron Adequate (IA) vs. Females:Iron Deficient (ID)	0.5919	0.6794	-0.08757	0.09092	3	3	1.362	12
Males :Iron Adequate (IA) vs. Females:Iron Repletion (IR)	0.5919	0.5716	0.02023	0.09092	3	3	0.3147	12
Males :Iron Deficient (ID) vs. Males :Iron Repletion (IR)	0.6205	1.133	-0.5124	0.09092	3	3	7.97	12
Males :Iron Deficient (ID) vs. Females:Iron Adequate (IA)	0.6205	0.5617	0.05873	0.09092	3	3	0.9136	12
Males :Iron Deficient (ID) vs. Females:Iron Deficient (ID)	0.6205	0.6794	-0.05897	0.09092	3	3	0.9172	12
Males :Iron Deficient (ID) vs. Females:Iron Repletion (IR)	0.6205	0.5716	0.04883	0.09092	3	3	0.7596	12
Males :Iron Repletion (IR) vs. Females:Iron Adequate (IA)	1.133	0.5617	0.5711	0.09092	3	3	8.884	12
Males :Iron Repletion (IR) vs. Females:Iron Deficient (ID)	1.133	0.6794	0.4534	0.09092	3	3	7.053	12
Males :Iron Repletion (IR) vs. Females:Iron Repletion (IR)	1.133	0.5716	0.5612	0.09092	3	3	8.73	12
Females:Iron Adequate (IA) vs. Females:Iron Deficient (ID)	0.5617	0.6794	-0.1177	0.09092	3	3	1.831	12
Females:Iron Adequate (IA) vs. Females:Iron Repletion (IR)	0.5617	0.5716	-0.0099	0.09092	3	3	0.154	12
Females:Iron Deficient (ID) vs. Females:Iron Repletion (IR)	0.6794	0.5716	0.1078	0.09092	3	3	1.677	12

Figure 6D

Table Analyzed	**Figure 6D** LCN2 WB Sel x diet							
Two-way ANOVA	Ordinary							
Alpha	0.05							
Source of Variation	% of total variation	P value	P value sun	Significant?				
Interaction	39.09	0.0077	**	Yes				
Sex	14.37	0.037	*	Yes				
Diets	15.22	0.0929	ns	No				
ANOVA table	SS	DF	MS	F (DFn, DFd)	P value			
Interaction	0.08428	2	0.04214	F (2, 12) = 7.490	P=0.0077			
Sex	0.03098	1	0.03098	F (1, 12) = 5.505	P=0.0370			
Diets	0.03281	2	0.01641	F (2, 12) = 2.916	P=0.0929			
Residual	0.06752	12	0.005627					
Difference between row means								
Mean of Males	0.4745							
Mean of Females	0.5575							
Difference between means	-0.08297							
SE of difference	0.03536							
95% CI of difference	-0.1600 to -0.005923							
Data summary								
Number of columns (Diets)	3							
Number of rows (Sex)	2							
Number of values	18							
Number of families	1							
Number of comparisons per family	15							
Alpha	0.05							
Tukey's multiple comparisons test	Mean Diff.	95.00% CI of diff.	Below thres	Summary	Adjusted P Value			
Males :Iron Adequate (IA) vs. Males :Iron Deficient (ID)	0.2573	0.05155 to 0.4630	Yes	*	0.0121			
Males :Iron Adequate (IA) vs. Males :Iron Repletion (IR)	0.1696	-0.03612 to 0.3753	No	ns	0.1312			
Males :Iron Adequate (IA) vs. Females:Iron Adequate (IA)	0.1091	-0.09659 to 0.3149	No	ns	0.5101			
Males :Iron Adequate (IA) vs. Females:Iron Deficient (ID)	0.05783	-0.1479 to 0.2636	No	ns	0.9268			
Males :Iron Adequate (IA) vs. Females:Iron Repletion (IR)	0.011	-0.1947 to 0.2167	No	ns	>0.9999			
Males :Iron Deficient (ID) vs. Males :Iron Repletion (IR)	-0.08767	-0.2934 to 0.1181	No	ns	0.7094			
Males :Iron Deficient (ID) vs. Females:Iron Adequate (IA)	-0.1481	-0.3539 to 0.05759	No	ns	0.2238			
Males :Iron Deficient (ID) vs. Females:Iron Deficient (ID)	-0.1994	-0.4052 to 0.006286	No	ns	0.0593			
Males :Iron Deficient (ID) vs. Females:Iron Repletion (IR)	-0.2463	-0.4520 to -0.04055	Yes	*	0.0164			
Males :Iron Repletion (IR) vs. Females:Iron Adequate (IA)	-0.06047	-0.2662 to 0.1453	No	ns	0.9135			
Males :Iron Repletion (IR) vs. Females:Iron Deficient (ID)	-0.1118	-0.3175 to 0.09395	No	ns	0.4865			
Males :Iron Repletion (IR) vs. Females:Iron Repletion (IR)	-0.1586	-0.3643 to 0.04712	No	ns	0.1734			
Females:Iron Adequate (IA) vs. Females:Iron Deficient (ID)	-0.0513	-0.2570 to 0.1544	No	ns	0.9543			
Females:Iron Adequate (IA) vs. Females:Iron Repletion (IR)	-0.09813	-0.3039 to 0.1076	No	ns	0.6121			
Females:Iron Deficient (ID) vs. Females:Iron Repletion (IR)	-0.04683	-0.2526 to 0.1589	No	ns	0.9686			
Test details	Mean 1	Mean 2	Mean Diff.	SE of diff.	N1	N2	q	DF
Males :Iron Adequate (IA) vs. Males :Iron Deficient (ID)	0.6168	0.3596	0.2573	0.06125	3	3	5.94	12
Males :Iron Adequate (IA) vs. Males :Iron Repletion (IR)	0.6168	0.4472	0.1696	0.06125	3	3	3.916	12
Males :Iron Adequate (IA) vs. Females:Iron Adequate (IA)	0.6168	0.5077	0.1091	0.06125	3	3	2.52	12
Males :Iron Adequate (IA) vs. Females:Iron Deficient (ID)	0.6168	0.559	0.05783	0.06125	3	3	1.335	12
Males :Iron Adequate (IA) vs. Females:Iron Repletion (IR)	0.6168	0.6058	0.011	0.06125	3	3	0.254	12
Males :Iron Deficient (ID) vs. Males :Iron Repletion (IR)	0.3596	0.4472	-0.08767	0.06125	3	3	2.024	12
Males :Iron Deficient (ID) vs. Females:Iron Adequate (IA)	0.3596	0.5077	-0.1481	0.06125	3	3	3.421	12
Males :Iron Deficient (ID) vs. Females:Iron Deficient (ID)	0.3596	0.559	-0.1994	0.06125	3	3	4.605	12
Males :Iron Deficient (ID) vs. Females:Iron Repletion (IR)	0.3596	0.6058	-0.2463	0.06125	3	3	5.686	12
Males :Iron Repletion (IR) vs. Females:Iron Adequate (IA)	0.4472	0.5077	-0.06047	0.06125	3	3	1.396	12
Males :Iron Repletion (IR) vs. Females:Iron Deficient (ID)	0.4472	0.559	-0.1118	0.06125	3	3	2.581	12
Males :Iron Repletion (IR) vs. Females:Iron Repletion (IR)	0.4472	0.6058	-0.1586	0.06125	3	3	3.662	12
Females:Iron Adequate (IA) vs. Females:Iron Deficient (ID)	0.5077	0.559	-0.0513	0.06125	3	3	1.185	12
Females:Iron Adequate (IA) vs. Females:Iron Repletion (IR)	0.5077	0.6058	-0.09813	0.06125	3	3	2.266	12
Females:Iron Deficient (ID) vs. Females:Iron Repletion (IR)	0.559	0.6058	-0.04683	0.06125	3	3	1.081	12

Figure 6E

Table Analyzed	**Figure 6E** LCN-R WB Veh x diet							
Two-way ANOVA	Ordinary							
Alpha	0.05							
Source of Variation	% of total variation	P value	P value sun	Significant?				
Interaction	3.254	0.2494	ns	No				
Sex	79.06	<0.0001	****	Yes				
Diet	0.9544	0.6409	ns	No				
ANOVA table	SS (Type III)	DF	MS	F (DFn, DFd)	P value			
Interaction	0.04105	2	0.02052	F (2, 11) = 1.580	P=0.2494			
Sex	0.9972	1	0.9972	F (1, 11) = 76.76	P<0.0001			
Diet	0.01204	2	0.006019	F (2, 11) = 0.4633	P=0.6409			
Residual	0.1429	11	0.01299					
Difference between row means								
Predicted (LS) mean of Males	0.6955							
Predicted (LS) mean of Females	1.186							
Difference between predicted means	-0.49							
SE of difference	0.05592							
95% CI of difference	-0.6131 to -0.3669							
Data summary								
Number of columns (Diet)	3							
Number of rows (Sex)	2							
Number of values	17							
Number of families	1							
Number of comparisons per family	15							
Alpha	0.05							
Tukey's multiple comparisons test	Predicted (LS) mear	95.00% CI of diff.	Below thres	Summary	Adjusted P Value			
Males:Iron Adequate (IA) vs. Males:Iron Deficient (ID)	-0.07997	-0.3973 to 0.2374	No	ns	0.9488			
Males:Iron Adequate (IA) vs. Males:Iron Repletion (IR)	-0.191	-0.5458 to 0.1638	No	ns	0.4835			
Males:Iron Adequate (IA) vs. Females:Iron Adequate (IA)	-0.6094	-0.9268 to -0.2920	Yes	***	0.0004			
Males:Iron Adequate (IA) vs. Females:Iron Deficient (ID)	-0.5792	-0.8966 to -0.2619	Yes	***	0.0007			
Males:Iron Adequate (IA) vs. Females:Iron Repletion (IR)	-0.5523	-0.8696 to -0.2349	Yes	**	0.001			
Males:Iron Deficient (ID) vs. Males:Iron Repletion (IR)	-0.111	-0.4659 to 0.2438	No	ns	0.8845			
Males:Iron Deficient (ID) vs. Females:Iron Adequate (IA)	-0.5294	-0.8468 to -0.2121	Yes	**	0.0015			
Males:Iron Deficient (ID) vs. Females:Iron Deficient (ID)	-0.4993	-0.8166 to -0.1819	Yes	**	0.0024			
Males:Iron Deficient (ID) vs. Females:Iron Repletion (IR)	-0.4723	-0.7897 to -0.1549	Yes	**	0.0036			
Males:Iron Repletion (IR) vs. Females:Iron Adequate (IA)	-0.4184	-0.7732 to -0.06356	Yes	*	0.0188			
Males:Iron Repletion (IR) vs. Females:Iron Deficient (ID)	-0.3882	-0.7431 to -0.03339	Yes	*	0.0299			
Males:Iron Repletion (IR) vs. Females:Iron Repletion (IR)	-0.3613	-0.7161 to -0.006423	Yes	*	0.0453			
Females:Iron Adequate (IA) vs. Females:Iron Deficient (ID)	0.03017	-0.2872 to 0.3475	No	ns	0.9994			
Females:Iron Adequate (IA) vs. Females:Iron Repletion (IR)	0.05713	-0.2602 to 0.3745	No	ns	0.9876			
Females:Iron Deficient (ID) vs. Females:Iron Repletion (IR)	0.02697	-0.2904 to 0.3443	No	ns	0.9996			
Test details	Predicted (LS) mear	Predicted (LS) mean 2	Predicted (L	SE of diff.	N1	N2	q	DF
Males:Iron Adequate (IA) vs. Males:Iron Deficient (ID)	0.6052	0.6852	-0.07997	0.09306	3	3	1.215	11
Males:Iron Adequate (IA) vs. Males:Iron Repletion (IR)	0.6052	0.7962	-0.191	0.104	3	2	2.596	11
Males:Iron Adequate (IA) vs. Females:Iron Adequate (IA)	0.6052	1.215	-0.6094	0.09306	3	3	9.261	11
Males:Iron Adequate (IA) vs. Females:Iron Deficient (ID)	0.6052	1.184	-0.5792	0.09306	3	3	8.802	11
Males:Iron Adequate (IA) vs. Females:Iron Repletion (IR)	0.6052	1.157	-0.5523	0.09306	3	3	8.392	11
Males:Iron Deficient (ID) vs. Males:Iron Repletion (IR)	0.6852	0.7962	-0.111	0.104	3	2	1.509	11
Males:Iron Deficient (ID) vs. Females:Iron Adequate (IA)	0.6852	1.215	-0.5294	0.09306	3	3	8.045	11
Males:Iron Deficient (ID) vs. Females:Iron Deficient (ID)	0.6852	1.184	-0.4993	0.09306	3	3	7.587	11
Males:Iron Deficient (ID) vs. Females:Iron Repletion (IR)	0.6852	1.157	-0.4723	0.09306	3	3	7.177	11
Males:Iron Repletion (IR) vs. Females:Iron Adequate (IA)	0.7962	1.215	-0.4184	0.104	2	3	5.687	11
Males:Iron Repletion (IR) vs. Females:Iron Deficient (ID)	0.7962	1.184	-0.3882	0.104	2	3	5.277	11
Males:Iron Repletion (IR) vs. Females:Iron Repletion (IR)	0.7962	1.157	-0.3613	0.104	2	3	4.91	11
Females:Iron Adequate (IA) vs. Females:Iron Deficient (ID)	1.215	1.184	0.03017	0.09306	3	3	0.4584	11
Females:Iron Adequate (IA) vs. Females:Iron Repletion (IR)	1.215	1.157	0.05713	0.09306	3	3	0.8682	11
Females:Iron Deficient (ID) vs. Females:Iron Repletion (IR)	1.184	1.157	0.02697	0.09306	3	3	0.4098	11

Figure 6F

Table Analyzed	**Figure 6F** LCN-R WB LD x diet						
Two-way ANOVA	Ordinary						
Alpha	0.05						
Source of Variation	% of total variation	P value	P value sun	Significant?			
Interaction	13.65	0.2433	ns	No			
Sex	30.47	0.0205	*	Yes			
Diet	4.463	0.6068	ns	No			
ANOVA table	SS	DF	MS	F (DFn, DFd)	P value		
Interaction	0.03468	2	0.01734	F (2, 12) = 1.594	P=0.2433		
Sex	0.07741	1	0.07741	F (1, 12) = 7.114	P=0.0205		
Diet	0.01134	2	0.005668	F (2, 12) = 0.5209	P=0.6068		
Residual	0.1306	12	0.01088				
Difference between row means							
Mean of Males	0.7195						
Mean of Females	0.8507						
Difference between means	-0.1312						
SE of difference	0.04917						
95% CI of difference	-0.2383 to -0.02401						
Data summary							
Number of columns (Diet)	3						
Number of rows (Sex)	2						
Number of values	18						
Number of families	1						
Number of comparisons per family	15						
Alpha	0.05						
Tukey's multiple comparisons test	Mean Diff.	95.00% CI of diff.	Below thres	Summary	Adjusted P Value		
Males:Iron Adequate (IA) vs. Males:Iron Deficient (ID)	-0.1495	-0.4356 to 0.1366	No	ns	0.5252		
Males:Iron Adequate (IA) vs. Males:Iron Repletion (IR)	-0.0105	-0.2966 to 0.2756	No	ns	>0.9999		
Males:Iron Adequate (IA) vs. Females:Iron Adequate (IA)	-0.187	-0.4731 to 0.09909	No	ns	0.3067		
Males:Iron Adequate (IA) vs. Females:Iron Deficient (ID)	-0.1567	-0.4428 to 0.1294	No	ns	0.4786		
Males:Iron Adequate (IA) vs. Females:Iron Repletion (IR)	-0.2098	-0.4959 to 0.07632	No	ns	0.2097		
Males:Iron Deficient (ID) vs. Males:Iron Repletion (IR)	0.139	-0.1471 to 0.4251	No	ns	0.5953		
Males:Iron Deficient (ID) vs. Females:Iron Adequate (IA)	-0.03753	-0.3236 to 0.2486	No	ns	0.9973		
Males:Iron Deficient (ID) vs. Females:Iron Deficient (ID)	-0.0072	-0.2933 to 0.2789	No	ns	>0.9999		
Males:Iron Deficient (ID) vs. Females:Iron Repletion (IR)	-0.0603	-0.3464 to 0.2258	No	ns	0.9773		
Males:Iron Repletion (IR) vs. Females:Iron Adequate (IA)	-0.1765	-0.4626 to 0.1096	No	ns	0.361		
Males:Iron Repletion (IR) vs. Females:Iron Deficient (ID)	-0.1462	-0.4323 to 0.1399	No	ns	0.547		
Males:Iron Repletion (IR) vs. Females:Iron Repletion (IR)	-0.1993	-0.4854 to 0.08682	No	ns	0.2509		
Females:Iron Adequate (IA) vs. Females:Iron Deficient (ID)	0.03033	-0.2558 to 0.3164	No	ns	0.999		
Females:Iron Adequate (IA) vs. Females:Iron Repletion (IR)	-0.02277	-0.3089 to 0.2633	No	ns	0.9998		
Females:Iron Deficient (ID) vs. Females:Iron Repletion (IR)	-0.0531	-0.3392 to 0.2330	No	ns	0.987		
Test details	Mean 1	Mean 2	Mean Diff.	SE of diff.	N1	N2	q	DF
Males:Iron Adequate (IA) vs. Males:Iron Deficient (ID)	0.6662	0.8157	-0.1495	0.08517	3	3	2.482	12
Males:Iron Adequate (IA) vs. Males:Iron Repletion (IR)	0.6662	0.6767	-0.0105	0.08517	3	3	0.1743	12
Males:Iron Adequate (IA) vs. Females:Iron Adequate (IA)	0.6662	0.8532	-0.187	0.08517	3	3	3.105	12
Males:Iron Adequate (IA) vs. Females:Iron Deficient (ID)	0.6662	0.8229	-0.1567	0.08517	3	3	2.601	12
Males:Iron Adequate (IA) vs. Females:Iron Repletion (IR)	0.6662	0.876	-0.2098	0.08517	3	3	3.483	12
Males:Iron Deficient (ID) vs. Males:Iron Repletion (IR)	0.8157	0.6767	0.139	0.08517	3	3	2.307	12
Males:Iron Deficient (ID) vs. Females:Iron Adequate (IA)	0.8157	0.8532	-0.03753	0.08517	3	3	0.6232	12
Males:Iron Deficient (ID) vs. Females:Iron Deficient (ID)	0.8157	0.8229	-0.0072	0.08517	3	3	0.1195	12
Males:Iron Deficient (ID) vs. Females:Iron Repletion (IR)	0.8157	0.876	-0.0603	0.08517	3	3	1.001	12
Males:Iron Repletion (IR) vs. Females:Iron Adequate (IA)	0.6767	0.8532	-0.1765	0.08517	3	3	2.931	12
Males:Iron Repletion (IR) vs. Females:Iron Deficient (ID)	0.6767	0.8229	-0.1462	0.08517	3	3	2.427	12
Males:Iron Repletion (IR) vs. Females:Iron Repletion (IR)	0.6767	0.876	-0.1993	0.08517	3	3	3.309	12
Females:Iron Adequate (IA) vs. Females:Iron Deficient (ID)	0.8532	0.8229	0.03033	0.08517	3	3	0.5037	12
Females:Iron Adequate (IA) vs. Females:Iron Repletion (IR)	0.8532	0.876	-0.02277	0.08517	3	3	0.378	12
Females:Iron Deficient (ID) vs. Females:Iron Repletion (IR)	0.8229	0.876	-0.0531	0.08517	3	3	0.8817	12

Figure 6G

Table Analyzed	**Figure 6G** LCN-R WB Sel x diet							
Two-way ANOVA	Ordinary							
Alpha	0.05							
Source of Variation	% of total variation	P value	P value sun	Significant?				
Interaction	0.03581	0.9946	ns	No				
Sex	42.02	0.004	**	Yes				
Diet	17.92	0.1086	ns	No				
ANOVA table	SS	DF	MS	F (DFn, DFd)	P value			
Interaction	0.0001147	2	5.73E-05	F (2, 12) = 0.005368	P=0.9946			
Sex	0.1345	1	0.1345	F (1, 12) = 12.60	P=0.0040			
Diet	0.05738	2	0.02869	F (2, 12) = 2.686	P=0.1086			
Residual	0.1282	12	0.01068					
Difference between row means								
Mean of Males	0.9682							
Mean of Females	0.7953							
Difference between means	0.1729							
SE of difference	0.04872							
95% CI of difference	0.06677 to 0.2791							
Data summary								
Number of columns (Diet)	3							
Number of rows (Sex)	2							
Number of values	18							
Number of families	1							
Number of comparisons per family	15							
Alpha	0.05							
Tukey's multiple comparisons test	Mean Diff.	95.00% CI of diff.	Below thres	Summary	Adjusted P Value			
Males:Iron Adequate (IA) vs. Males:Iron Deficient (ID)	-0.01213	-0.2956 to 0.2713	No	ns	>0.9999			
Males:Iron Adequate (IA) vs. Males:Iron Repletion (IR)	-0.1227	-0.4061 to 0.1607	No	ns	0.6967			
Males:Iron Adequate (IA) vs. Females:Iron Adequate (IA)	0.1698	-0.1137 to 0.4532	No	ns	0.3896			
Males:Iron Adequate (IA) vs. Females:Iron Deficient (ID)	0.1679	-0.1155 to 0.4513	No	ns	0.4005			
Males:Iron Adequate (IA) vs. Females:Iron Repletion (IR)	0.04623	-0.2372 to 0.3297	No	ns	0.9927			
Males:Iron Deficient (ID) vs. Males:Iron Repletion (IR)	-0.1106	-0.3940 to 0.1729	No	ns	0.7746			
Males:Iron Deficient (ID) vs. Females:Iron Adequate (IA)	0.1819	-0.1015 to 0.4653	No	ns	0.3235			
Males:Iron Deficient (ID) vs. Females:Iron Deficient (ID)	0.18	-0.1034 to 0.4635	No	ns	0.3332			
Males:Iron Deficient (ID) vs. Females:Iron Repletion (IR)	0.05837	-0.2251 to 0.3418	No	ns	0.9795			
Males:Iron Repletion (IR) vs. Females:Iron Adequate (IA)	0.2925	0.009049 to 0.5759	Yes	*	0.0418			
Males:Iron Repletion (IR) vs. Females:Iron Deficient (ID)	0.2906	0.007182 to 0.5740	Yes	*	0.0433			
Males:Iron Repletion (IR) vs. Females:Iron Repletion (IR)	0.1689	-0.1145 to 0.4524	No	ns	0.3945			
Females:Iron Adequate (IA) vs. Females:Iron Deficient (ID)	-0.001867	-0.2853 to 0.2816	No	ns	>0.9999			
Females:Iron Adequate (IA) vs. Females:Iron Repletion (IR)	-0.1235	-0.4070 to 0.1599	No	ns	0.6911			
Females:Iron Deficient (ID) vs. Females:Iron Repletion (IR)	-0.1217	-0.4051 to 0.1618	No	ns	0.7035			
Test details	Mean 1	Mean 2	Mean Diff.	SE of diff.	N1	N2	q	DF
Males:Iron Adequate (IA) vs. Males:Iron Deficient (ID)	0.9233	0.9354	-0.01213	0.08438	3	3	0.2034	12
Males:Iron Adequate (IA) vs. Males:Iron Repletion (IR)	0.9233	1.046	-0.1227	0.08438	3	3	2.057	12
Males:Iron Adequate (IA) vs. Females:Iron Adequate (IA)	0.9233	0.7535	0.1698	0.08438	3	3	2.845	12
Males:Iron Adequate (IA) vs. Females:Iron Deficient (ID)	0.9233	0.7554	0.1679	0.08438	3	3	2.814	12
Males:Iron Adequate (IA) vs. Females:Iron Repletion (IR)	0.9233	0.8771	0.04623	0.08438	3	3	0.7749	12
Males:Iron Deficient (ID) vs. Males:Iron Repletion (IR)	0.9354	1.046	-0.1106	0.08438	3	3	1.853	12
Males:Iron Deficient (ID) vs. Females:Iron Adequate (IA)	0.9354	0.7535	0.1819	0.08438	3	3	3.049	12
Males:Iron Deficient (ID) vs. Females:Iron Deficient (ID)	0.9354	0.7554	0.18	0.08438	3	3	3.017	12
Males:Iron Deficient (ID) vs. Females:Iron Repletion (IR)	0.9354	0.8771	0.05837	0.08438	3	3	0.9783	12
Males:Iron Repletion (IR) vs. Females:Iron Adequate (IA)	1.046	0.7535	0.2925	0.08438	3	3	4.902	12
Males:Iron Repletion (IR) vs. Females:Iron Deficient (ID)	1.046	0.7554	0.2906	0.08438	3	3	4.871	12
Males:Iron Repletion (IR) vs. Females:Iron Repletion (IR)	1.046	0.8771	0.1689	0.08438	3	3	2.831	12
Females:Iron Adequate (IA) vs. Females:Iron Deficient (ID)	0.7535	0.7554	-0.001867	0.08438	3	3	0.03129	12
Females:Iron Adequate (IA) vs. Females:Iron Repletion (IR)	0.7535	0.8771	-0.1235	0.08438	3	3	2.07	12
Females:Iron Deficient (ID) vs. Females:Iron Repletion (IR)	0.7554	0.8771	-0.1217	0.08438	3	3	2.039	12

Figure 6H

Table Analyzed	**Figure 6H** GFAP WB Veh x diet							
Two-way ANOVA	Ordinary							
Alpha	0.05							
Source of Variation	% of total variation	P value	P value sun	Significant?				
Interaction	32.01	0.0337	*	Yes				
Sex	2.539	0.4117	ns	No				
Diet	23.33	0.071	ns	No				
ANOVA table	SS	DF	MS	F (DFn, DFd)	P value			
Interaction	0.08539	2	0.04269	F (2, 12) = 4.559	P=0.0337			
Sex	0.006774	1	0.006774	F (1, 12) = 0.7235	P=0.4117			
Diet	0.06225	2	0.03112	F (2, 12) = 3.324	P=0.0710			
Residual	0.1124	12	0.009364					
Difference between row means								
Mean of Males	0.8639							
Mean of Females	0.9027							
Difference between means	-0.0388							
SE of difference	0.04562							
95% CI of difference	-0.1382 to 0.06059							
Data summary								
Number of columns (Diet)	3							
Number of rows (Sex)	2							
Number of values	18							
Number of families	1							
Number of comparisons per family	15							
Alpha	0.05							
Tukey's multiple comparisons test	Mean Diff.	95.00% CI of diff.	Below thres	Summary	Adjusted P Value			
Males :Iron Adequate (IA) vs. Males :Iron Deficient (ID)	0.1265	-0.1389 to 0.3919	No	ns	0.6126			
Males :Iron Adequate (IA) vs. Males :Iron Repletion (IR)	0.0693	-0.1961 to 0.3347	No	ns	0.9451			
Males :Iron Adequate (IA) vs. Females:Iron Adequate (IA)	0.131	-0.1344 to 0.3964	No	ns	0.5804			
Males :Iron Adequate (IA) vs. Females:Iron Deficient (ID)	0.0856	-0.1798 to 0.3510	No	ns	0.8789			
Males :Iron Adequate (IA) vs. Females:Iron Repletion (IR)	-0.1371	-0.4025 to 0.1283	No	ns	0.536			
Males :Iron Deficient (ID) vs. Males :Iron Repletion (IR)	-0.05723	-0.3226 to 0.2082	No	ns	0.975			
Males :Iron Deficient (ID) vs. Females:Iron Adequate (IA)	0.004433	-0.2610 to 0.2698	No	ns	>0.9999			
Males :Iron Deficient (ID) vs. Females:Iron Deficient (ID)	-0.04093	-0.3063 to 0.2245	No	ns	0.9944			
Males :Iron Deficient (ID) vs. Females:Iron Repletion (IR)	-0.2637	-0.5291 to 0.001722	No	ns	0.0519			
Males :Iron Repletion (IR) vs. Females:Iron Adequate (IA)	0.06167	-0.2037 to 0.3271	No	ns	0.9658			
Males :Iron Repletion (IR) vs. Females:Iron Deficient (ID)	0.0163	-0.2491 to 0.2817	No	ns	>0.9999			
Males :Iron Repletion (IR) vs. Females:Iron Repletion (IR)	-0.2064	-0.4718 to 0.05896	No	ns	0.1673			
Females:Iron Adequate (IA) vs. Females:Iron Deficient (ID)	-0.04537	-0.3108 to 0.2200	No	ns	0.991			
Females:Iron Adequate (IA) vs. Females:Iron Repletion (IR)	-0.2681	-0.5335 to -0.002711	Yes	*	0.0472			
Females:Iron Deficient (ID) vs. Females:Iron Repletion (IR)	-0.2227	-0.4881 to 0.04266	No	ns	0.1212			
Test details	Mean 1	Mean 2	Mean Diff.	SE of diff.	N1	N2	q	DF
Males :Iron Adequate (IA) vs. Males :Iron Deficient (ID)	0.9291	0.8026	0.1265	0.07901	3	3	2.265	12
Males :Iron Adequate (IA) vs. Males :Iron Repletion (IR)	0.9291	0.8598	0.0693	0.07901	3	3	1.24	12
Males :Iron Adequate (IA) vs. Females:Iron Adequate (IA)	0.9291	0.7982	0.131	0.07901	3	3	2.344	12
Males :Iron Adequate (IA) vs. Females:Iron Deficient (ID)	0.9291	0.8435	0.0856	0.07901	3	3	1.532	12
Males :Iron Adequate (IA) vs. Females:Iron Repletion (IR)	0.9291	1.066	-0.1371	0.07901	3	3	2.455	12
Males :Iron Deficient (ID) vs. Males :Iron Repletion (IR)	0.8026	0.8598	-0.05723	0.07901	3	3	1.024	12
Males :Iron Deficient (ID) vs. Females:Iron Adequate (IA)	0.8026	0.7982	0.004433	0.07901	3	3	0.07935	12
Males :Iron Deficient (ID) vs. Females:Iron Deficient (ID)	0.8026	0.8435	-0.04093	0.07901	3	3	0.7327	12
Males :Iron Deficient (ID) vs. Females:Iron Repletion (IR)	0.8026	1.066	-0.2637	0.07901	3	3	4.719	12
Males :Iron Repletion (IR) vs. Females:Iron Adequate (IA)	0.8598	0.7982	0.06167	0.07901	3	3	1.104	12
Males :Iron Repletion (IR) vs. Females:Iron Deficient (ID)	0.8598	0.8435	0.0163	0.07901	3	3	0.2918	12
Males :Iron Repletion (IR) vs. Females:Iron Repletion (IR)	0.8598	1.066	-0.2064	0.07901	3	3	3.695	12
Females:Iron Adequate (IA) vs. Females:Iron Deficient (ID)	0.7982	0.8435	-0.04537	0.07901	3	3	0.812	12
Females:Iron Adequate (IA) vs. Females:Iron Repletion (IR)	0.7982	1.066	-0.2681	0.07901	3	3	4.799	12
Females:Iron Deficient (ID) vs. Females:Iron Repletion (IR)	0.8435	1.066	-0.2227	0.07901	3	3	3.987	12

Figure 6I

Table Analyzed	**Figure 6I** GFAP WB LD x diet							
Two-way ANOVA	Ordinary							
Alpha	0.05							
Source of Variation	% of total variation	P value	P value sun	Significant?				
Interaction	6.587	0.3816	ns	No				
Sex	6.678	0.1711	ns	No				
Diet	48.92	0.0069	**	Yes				
ANOVA table	SS	DF	MS	F (DFn, DFd)	P value			
Interaction	0.03007	2	0.01504	F (2, 12) = 1.045	P=0.3816			
Sex	0.03049	1	0.03049	F (1, 12) = 2.119	P=0.1711			
Diet	0.2233	2	0.1117	F (2, 12) = 7.761	P=0.0069			
Residual	0.1726	12	0.01439					
Difference between row means								
Mean of Males	0.8443							
Mean of Females	0.9266							
Difference between means	-0.08231							
SE of difference	0.05654							
95% CI of difference	-0.2055 to 0.04088							
Data summary								
Number of columns (Diet)	3							
Number of rows (Sex)	2							
Number of values	18							
Number of families	1							
Number of comparisons per family	15							
Alpha	0.05							
Tukey's multiple comparisons test	Mean Diff.	95.00% CI of diff.	Below thres	Summary	Adjusted P Value			
Males :Iron Adequate (IA) vs. Males :Iron Deficient (ID)	0.1542	-0.1748 to 0.4832	No	ns	0.6281			
Males :Iron Adequate (IA) vs. Males :Iron Repletion (IR)	-0.1812	-0.5101 to 0.1478	No	ns	0.4729			
Males :Iron Adequate (IA) vs. Females:Iron Adequate (IA)	-0.005833	-0.3348 to 0.3231	No	ns	>0.9999			
Males :Iron Adequate (IA) vs. Females:Iron Deficient (ID)	-0.04143	-0.3704 to 0.2875	No	ns	0.9978			
Males :Iron Adequate (IA) vs. Females:Iron Repletion (IR)	-0.2266	-0.5556 to 0.1023	No	ns	0.2602			
Males :Iron Deficient (ID) vs. Males :Iron Repletion (IR)	-0.3354	-0.6643 to -0.006414	Yes	*	0.0448			
Males :Iron Deficient (ID) vs. Females:Iron Adequate (IA)	-0.16	-0.4890 to 0.1689	No	ns	0.5938			
Males :Iron Deficient (ID) vs. Females:Iron Deficient (ID)	-0.1956	-0.5246 to 0.1333	No	ns	0.3967			
Males :Iron Deficient (ID) vs. Females:Iron Repletion (IR)	-0.3808	-0.7098 to -0.05188	Yes	*	0.0205			
Males :Iron Repletion (IR) vs. Females:Iron Adequate (IA)	0.1753	-0.1536 to 0.5043	No	ns	0.5054			
Males :Iron Repletion (IR) vs. Females:Iron Deficient (ID)	0.1397	-0.1892 to 0.4687	No	ns	0.7119			
Males :Iron Repletion (IR) vs. Females:Iron Repletion (IR)	-0.04547	-0.3744 to 0.2835	No	ns	0.9966			
Females:Iron Adequate (IA) vs. Females:Iron Deficient (ID)	-0.0356	-0.3646 to 0.2934	No	ns	0.9989			
Females:Iron Adequate (IA) vs. Females:Iron Repletion (IR)	-0.2208	-0.5498 to 0.1082	No	ns	0.2828			
Females:Iron Deficient (ID) vs. Females:Iron Repletion (IR)	-0.1852	-0.5142 to 0.1438	No	ns	0.451			
Test details	Mean 1	Mean 2	Mean Diff.	SE of diff.	N1	N2	q	DF
Males :Iron Adequate (IA) vs. Males :Iron Deficient (ID)	0.8353	0.6811	0.1542	0.09793	3	3	2.227	12
Males :Iron Adequate (IA) vs. Males :Iron Repletion (IR)	0.8353	1.016	-0.1812	0.09793	3	3	2.616	12
Males :Iron Adequate (IA) vs. Females:Iron Adequate (IA)	0.8353	0.8411	-0.005833	0.09793	3	3	0.08424	12
Males :Iron Adequate (IA) vs. Females:Iron Deficient (ID)	0.8353	0.8767	-0.04143	0.09793	3	3	0.5983	12
Males :Iron Adequate (IA) vs. Females:Iron Repletion (IR)	0.8353	1.062	-0.2266	0.09793	3	3	3.273	12
Males :Iron Deficient (ID) vs. Males :Iron Repletion (IR)	0.6811	1.016	-0.3354	0.09793	3	3	4.843	12
Males :Iron Deficient (ID) vs. Females:Iron Adequate (IA)	0.6811	0.8411	-0.16	0.09793	3	3	2.311	12
Males :Iron Deficient (ID) vs. Females:Iron Deficient (ID)	0.6811	0.8767	-0.1956	0.09793	3	3	2.825	12
Males :Iron Deficient (ID) vs. Females:Iron Repletion (IR)	0.6811	1.062	-0.3808	0.09793	3	3	5.499	12
Males :Iron Repletion (IR) vs. Females:Iron Adequate (IA)	1.016	0.8411	0.1753	0.09793	3	3	2.532	12
Males :Iron Repletion (IR) vs. Females:Iron Deficient (ID)	1.016	0.8767	0.1397	0.09793	3	3	2.018	12
Males :Iron Repletion (IR) vs. Females:Iron Repletion (IR)	1.016	1.062	-0.04547	0.09793	3	3	0.6566	12
Females:Iron Adequate (IA) vs. Females:Iron Deficient (ID)	0.8411	0.8767	-0.0356	0.09793	3	3	0.5141	12
Females:Iron Adequate (IA) vs. Females:Iron Repletion (IR)	0.8411	1.062	-0.2208	0.09793	3	3	3.188	12
Females:Iron Deficient (ID) vs. Females:Iron Repletion (IR)	0.8767	1.062	-0.1852	0.09793	3	3	2.674	12

Figure 6J

Table Analyzed	**Figure 6J** GFAP WB Sel x diet							
Two-way ANOVA	Ordinary							
Alpha	0.05							
Source of Variation	% of total variation	P value	P value sun	Significant?				
Interaction	39.75	0.0324	*	Yes				
Sex	7.739	0.2043	ns	No				
Diet	0.9767	0.8935	ns	No				
ANOVA table	SS	DF	MS	F (DFn, DFd)	P value			
Interaction	0.2291	2	0.1145	F (2, 12) = 4.628	P=0.0324			
Sex	0.0446	1	0.0446	F (1, 12) = 1.802	P=0.2043			
Diet	0.005629	2	0.002814	F (2, 12) = 0.1137	P=0.8935			
Residual	0.297	12	0.02475					
Difference between row means								
Mean of Males	0.9734							
Mean of Females	1.073							
Difference between means	-0.09956							
SE of difference	0.07416							
95% CI of difference	-0.2611 to 0.06203							
Data summary								
Number of columns (Diet)	3							
Number of rows (Sex)	2							
Number of values	18							
Number of families	1							
Number of comparisons per family	15							
Alpha	0.05							
Tukey's multiple comparisons test	Mean Diff.	95.00% CI of diff.	Below thres	Summary	Adjusted P Value			
Males :Iron Adequate (IA) vs. Males :Iron Deficient (ID)	0.01493	-0.4165 to 0.4464	No	ns	>0.9999			
Males :Iron Adequate (IA) vs. Males :Iron Repletion (IR)	-0.2427	-0.6741 to 0.1888	No	ns	0.452			
Males :Iron Adequate (IA) vs. Females:Iron Adequate (IA)	-0.2016	-0.6331 to 0.2299	No	ns	0.631			
Males :Iron Adequate (IA) vs. Females:Iron Deficient (ID)	-0.2954	-0.7269 to 0.1360	No	ns	0.2654			
Males :Iron Adequate (IA) vs. Females:Iron Repletion (IR)	-0.02937	-0.4608 to 0.4021	No	ns	0.9999			
Males :Iron Deficient (ID) vs. Males :Iron Repletion (IR)	-0.2576	-0.6891 to 0.1739	No	ns	0.3928			
Males :Iron Deficient (ID) vs. Females:Iron Adequate (IA)	-0.2165	-0.6480 to 0.2149	No	ns	0.5643			
Males :Iron Deficient (ID) vs. Females:Iron Deficient (ID)	-0.3104	-0.7418 to 0.1211	No	ns	0.2246			
Males :Iron Deficient (ID) vs. Females:Iron Repletion (IR)	-0.0443	-0.4758 to 0.3872	No	ns	0.9992			
Males :Iron Repletion (IR) vs. Females:Iron Adequate (IA)	0.04107	-0.3904 to 0.4725	No	ns	0.9994			
Males :Iron Repletion (IR) vs. Females:Iron Deficient (ID)	-0.05277	-0.4842 to 0.3787	No	ns	0.9981			
Males :Iron Repletion (IR) vs. Females:Iron Repletion (IR)	0.2133	-0.2182 to 0.6448	No	ns	0.5787			
Females:Iron Adequate (IA) vs. Females:Iron Deficient (ID)	-0.09383	-0.5253 to 0.3376	No	ns	0.9741			
Females:Iron Adequate (IA) vs. Females:Iron Repletion (IR)	0.1722	-0.2592 to 0.6037	No	ns	0.7586			
Females:Iron Deficient (ID) vs. Females:Iron Repletion (IR)	0.2661	-0.1654 to 0.6975	No	ns	0.3614			
Test details	Mean 1	Mean 2	Mean Diff.	SE of diff.	N1	N2	q	DF
Males :Iron Adequate (IA) vs. Males :Iron Deficient (ID)	0.8975	0.8826	0.01493	0.1285	3	3	0.1644	12
Males :Iron Adequate (IA) vs. Males :Iron Repletion (IR)	0.8975	1.14	-0.2427	0.1285	3	3	2.672	12
Males :Iron Adequate (IA) vs. Females:Iron Adequate (IA)	0.8975	1.099	-0.2016	0.1285	3	3	2.22	12
Males :Iron Adequate (IA) vs. Females:Iron Deficient (ID)	0.8975	1.193	-0.2954	0.1285	3	3	3.253	12
Males :Iron Adequate (IA) vs. Females:Iron Repletion (IR)	0.8975	0.9269	-0.02937	0.1285	3	3	0.3233	12
Males :Iron Deficient (ID) vs. Males :Iron Repletion (IR)	0.8826	1.14	-0.2576	0.1285	3	3	2.836	12
Males :Iron Deficient (ID) vs. Females:Iron Adequate (IA)	0.8826	1.099	-0.2165	0.1285	3	3	2.384	12
Males :Iron Deficient (ID) vs. Females:Iron Deficient (ID)	0.8826	1.193	-0.3104	0.1285	3	3	3.417	12
Males :Iron Deficient (ID) vs. Females:Iron Repletion (IR)	0.8826	0.9269	-0.0443	0.1285	3	3	0.4877	12
Males :Iron Repletion (IR) vs. Females:Iron Adequate (IA)	1.14	1.099	0.04107	0.1285	3	3	0.4521	12
Males :Iron Repletion (IR) vs. Females:Iron Deficient (ID)	1.14	1.193	-0.05277	0.1285	3	3	0.5809	12
Males :Iron Repletion (IR) vs. Females:Iron Repletion (IR)	1.14	0.9269	0.2133	0.1285	3	3	2.348	12
Females:Iron Adequate (IA) vs. Females:Iron Deficient (ID)	1.099	1.193	-0.09383	0.1285	3	3	1.033	12
Females:Iron Adequate (IA) vs. Females:Iron Repletion (IR)	1.099	0.9269	0.1722	0.1285	3	3	1.896	12
Females:Iron Deficient (ID) vs. Females:Iron Repletion (IR)	1.193	0.9269	0.2661	0.1285	3	3	2.929	12

Figure 7B

Table Analyzed	**Figure 7B** CAT WB Veh x diet							
Two-way ANOVA	Ordinary							
Alpha	0.05							
Source of Variation	% of total variation	P value	P value sun	Significant?				
Interaction	5.827	0.4076	ns	No				
Sex	28.37	0.0097	**	Yes				
Diet	29.68	0.0274	*	Yes				
ANOVA table	SS	DF	MS	F (DFn, DFd)	P value			
Interaction	0.01533	2	0.007666	F (2, 12) = 0.9680	P=0.4076			
Sex	0.07465	1	0.07465	F (1, 12) = 9.427	P=0.0097			
Diet	0.0781	2	0.03905	F (2, 12) = 4.931	P=0.0274			
Residual	0.09503	12	0.007919					
Difference between row means								
Mean of Males	1.007							
Mean of Females	1.136							
Difference between means	-0.1288							
SE of difference	0.04195							
95% CI of difference	-0.2202 to -0.03740							
Data summary								
Number of columns (Diet)	3							
Number of rows (Sex)	2							
Number of values	18							
Number of families	1							
Number of comparisons per family	15							
Alpha	0.05							
Tukey's multiple comparisons test	Mean Diff.	95.00% CI of diff.	Below thres	Summary	Adjusted P Value			
Males :Iron Adequate (IA) vs. Males :Iron Deficient (ID)	0.08177	-0.1623 to 0.3258	No	ns	0.8619			
Males :Iron Adequate (IA) vs. Males :Iron Repletion (IR)	0.006533	-0.2375 to 0.2506	No	ns	>0.9999			
Males :Iron Adequate (IA) vs. Females:Iron Adequate (IA)	-0.1586	-0.4027 to 0.08546	No	ns	0.312			
Males :Iron Adequate (IA) vs. Females:Iron Deficient (ID)	0.03453	-0.2095 to 0.2786	No	ns	0.9962			
Males :Iron Adequate (IA) vs. Females:Iron Repletion (IR)	-0.174	-0.4181 to 0.07002	No	ns	0.2316			
Males :Iron Deficient (ID) vs. Males :Iron Repletion (IR)	-0.07523	-0.3193 to 0.1688	No	ns	0.897			
Males :Iron Deficient (ID) vs. Females:Iron Adequate (IA)	-0.2404	-0.4844 to 0.003691	No	ns	0.0544			
Males :Iron Deficient (ID) vs. Females:Iron Deficient (ID)	-0.04723	-0.2913 to 0.1968	No	ns	0.9843			
Males :Iron Deficient (ID) vs. Females:Iron Repletion (IR)	-0.2558	-0.4999 to -0.01174	Yes	*	0.0381			
Males :Iron Repletion (IR) vs. Females:Iron Adequate (IA)	-0.1651	-0.4092 to 0.07892	No	ns	0.2758			
Males :Iron Repletion (IR) vs. Females:Iron Deficient (ID)	0.028	-0.2161 to 0.2721	No	ns	0.9986			
Males :Iron Repletion (IR) vs. Females:Iron Repletion (IR)	-0.1806	-0.4246 to 0.06349	No	ns	0.2029			
Females:Iron Adequate (IA) vs. Females:Iron Deficient (ID)	0.1931	-0.05092 to 0.4372	No	ns	0.1561			
Females:Iron Adequate (IA) vs. Females:Iron Repletion (IR)	-0.01543	-0.2595 to 0.2286	No	ns	>0.9999			
Females:Iron Deficient (ID) vs. Females:Iron Repletion (IR)	-0.2086	-0.4526 to 0.03549	No	ns	0.1116			
Test details	Mean 1	Mean 2	Mean Diff.	SE of diff.	N1	N2	q	DF
Males :Iron Adequate (IA) vs. Males :Iron Deficient (ID)	1.037	0.955	0.08177	0.07266	3	3	1.591	12
Males :Iron Adequate (IA) vs. Males :Iron Repletion (IR)	1.037	1.03	0.006533	0.07266	3	3	0.1272	12
Males :Iron Adequate (IA) vs. Females:Iron Adequate (IA)	1.037	1.195	-0.1586	0.07266	3	3	3.087	12
Males :Iron Adequate (IA) vs. Females:Iron Deficient (ID)	1.037	1.002	0.03453	0.07266	3	3	0.6721	12
Males :Iron Adequate (IA) vs. Females:Iron Repletion (IR)	1.037	1.211	-0.174	0.07266	3	3	3.387	12
Males :Iron Deficient (ID) vs. Males :Iron Repletion (IR)	0.955	1.03	-0.07523	0.07266	3	3	1.464	12
Males :Iron Deficient (ID) vs. Females:Iron Adequate (IA)	0.955	1.195	-0.2404	0.07266	3	3	4.678	12
Males :Iron Deficient (ID) vs. Females:Iron Deficient (ID)	0.955	1.002	-0.04723	0.07266	3	3	0.9193	12
Males :Iron Deficient (ID) vs. Females:Iron Repletion (IR)	0.955	1.211	-0.2558	0.07266	3	3	4.979	12
Males :Iron Repletion (IR) vs. Females:Iron Adequate (IA)	1.03	1.195	-0.1651	0.07266	3	3	3.214	12
Males :Iron Repletion (IR) vs. Females:Iron Deficient (ID)	1.03	1.002	0.028	0.07266	3	3	0.545	12
Males :Iron Repletion (IR) vs. Females:Iron Repletion (IR)	1.03	1.211	-0.1806	0.07266	3	3	3.514	12
Females:Iron Adequate (IA) vs. Females:Iron Deficient (ID)	1.195	1.002	0.1931	0.07266	3	3	3.759	12
Females:Iron Adequate (IA) vs. Females:Iron Repletion (IR)	1.195	1.211	-0.01543	0.07266	3	3	0.3004	12
Females:Iron Deficient (ID) vs. Females:Iron Repletion (IR)	1.002	1.211	-0.2086	0.07266	3	3	4.059	12

Figure 7C

Table Analyzed	**Figure 7C** CAT WB LD x diet							
Two-way ANOVA	Ordinary							
Alpha	0.05							
Source of Variation	% of total variation	P value	P value sun	Significant?				
Interaction	13.42	0.2214	ns	No				
Sex	0.6133	0.6992	ns	No				
Diet	38.99	0.0266	*	Yes				
ANOVA table	SS	DF	MS	F (DFn, DFd)	P value			
Interaction	0.08383	2	0.04192	F (2, 12) = 1.714	P=0.2214			
Sex	0.003831	1	0.003831	F (1, 12) = 0.1567	P=0.6992			
Diet	0.2435	2	0.1218	F (2, 12) = 4.979	P=0.0266			
Residual	0.2934	12	0.02445					
Difference between row means								
Mean of Males	0.9592							
Mean of Females	0.9884							
Difference between means	-0.02918							
SE of difference	0.07372							
95% CI of difference	-0.1898 to 0.1314							
Data summary								
Number of columns (Diet)	3							
Number of rows (Sex)	2							
Number of values	18							
Number of families	1							
Number of comparisons per family	15							
Alpha	0.05							
Tukey's multiple comparisons test	Mean Diff.	95.00% CI of diff.	Below thres	Summary	Adjusted P Value			
Males :Iron Adequate (IA) vs. Males :Iron Deficient (ID)	0.1644	-0.2644 to 0.5933	No	ns	0.7861			
Males :Iron Adequate (IA) vs. Males :Iron Repletion (IR)	0.4289	6.678e-005 to 0.8578	Yes	*	0.05			
Males :Iron Adequate (IA) vs. Females:Iron Adequate (IA)	0.06807	-0.3608 to 0.4969	No	ns	0.9936			
Males :Iron Adequate (IA) vs. Females:Iron Deficient (ID)	0.231	-0.1978 to 0.6599	No	ns	0.4949			
Males :Iron Adequate (IA) vs. Females:Iron Repletion (IR)	0.2067	-0.2221 to 0.6356	No	ns	0.6024			
Males :Iron Deficient (ID) vs. Males :Iron Repletion (IR)	0.2645	-0.1644 to 0.6934	No	ns	0.3613			
Males :Iron Deficient (ID) vs. Females:Iron Adequate (IA)	-0.09637	-0.5252 to 0.3325	No	ns	0.9702			
Males :Iron Deficient (ID) vs. Females:Iron Deficient (ID)	0.0666	-0.3623 to 0.4955	No	ns	0.9942			
Males :Iron Deficient (ID) vs. Females:Iron Repletion (IR)	0.0423	-0.3866 to 0.4712	No	ns	0.9993			
Males :Iron Repletion (IR) vs. Females:Iron Adequate (IA)	-0.3609	-0.7897 to 0.06800	No	ns	0.1198			
Males :Iron Repletion (IR) vs. Females:Iron Deficient (ID)	-0.1979	-0.6268 to 0.2310	No	ns	0.6422			
Males :Iron Repletion (IR) vs. Females:Iron Repletion (IR)	-0.2222	-0.6511 to 0.2067	No	ns	0.5334			
Females:Iron Adequate (IA) vs. Females:Iron Deficient (ID)	0.163	-0.2659 to 0.5918	No	ns	0.7919			
Females:Iron Adequate (IA) vs. Females:Iron Repletion (IR)	0.1387	-0.2902 to 0.5675	No	ns	0.8779			
Females:Iron Deficient (ID) vs. Females:Iron Repletion (IR)	-0.0243	-0.4532 to 0.4046	No	ns	>0.9999			
Test details	Mean 1	Mean 2	Mean Diff.	SE of diff.	N1	N2	q	DF
Males :Iron Adequate (IA) vs. Males :Iron Deficient (ID)	1.157	0.9926	0.1644	0.1277	3	3	1.821	12
Males :Iron Adequate (IA) vs. Males :Iron Repletion (IR)	1.157	0.7281	0.4289	0.1277	3	3	4.751	12
Males :Iron Adequate (IA) vs. Females:Iron Adequate (IA)	1.157	1.089	0.06807	0.1277	3	3	0.7539	12
Males :Iron Adequate (IA) vs. Females:Iron Deficient (ID)	1.157	0.926	0.231	0.1277	3	3	2.559	12
Males :Iron Adequate (IA) vs. Females:Iron Repletion (IR)	1.157	0.9503	0.2067	0.1277	3	3	2.29	12
Males :Iron Deficient (ID) vs. Males :Iron Repletion (IR)	0.9926	0.7281	0.2645	0.1277	3	3	2.93	12
Males :Iron Deficient (ID) vs. Females:Iron Adequate (IA)	0.9926	1.089	-0.09637	0.1277	3	3	1.067	12
Males :Iron Deficient (ID) vs. Females:Iron Deficient (ID)	0.9926	0.926	0.0666	0.1277	3	3	0.7377	12
Males :Iron Deficient (ID) vs. Females:Iron Repletion (IR)	0.9926	0.9503	0.0423	0.1277	3	3	0.4685	12
Males :Iron Repletion (IR) vs. Females:Iron Adequate (IA)	0.7281	1.089	-0.3609	0.1277	3	3	3.997	12
Males :Iron Repletion (IR) vs. Females:Iron Deficient (ID)	0.7281	0.926	-0.1979	0.1277	3	3	2.192	12
Males :Iron Repletion (IR) vs. Females:Iron Repletion (IR)	0.7281	0.9503	-0.2222	0.1277	3	3	2.461	12
Females:Iron Adequate (IA) vs. Females:Iron Deficient (ID)	1.089	0.926	0.163	0.1277	3	3	1.805	12
Females:Iron Adequate (IA) vs. Females:Iron Repletion (IR)	1.089	0.9503	0.1387	0.1277	3	3	1.536	12
Females:Iron Deficient (ID) vs. Females:Iron Repletion (IR)	0.926	0.9503	-0.0243	0.1277	3	3	0.2692	12

Figure 7D

Table Analyzed	**Figure 7D** CAT WB Sel x diet							
Two-way ANOVA	Ordinary							
Alpha	0.05							
Source of Variation	% of total variation	P value	P value sun	Significant?				
Interaction	4.952	0.3246	ns	No				
Sex	1.913	0.3475	ns	No				
Diet	69.12	0.0003	***	Yes				
ANOVA table	SS	DF	MS	F (DFn, DFd)	P value			
Interaction	0.008126	2	0.004063	F (2, 12) = 1.238	P=0.3246			
Sex	0.003139	1	0.003139	F (1, 12) = 0.9561	P=0.3475			
Diet	0.1134	2	0.05671	F (2, 12) = 17.27	P=0.0003			
Residual	0.0394	12	0.003283					
Difference between row means								
Mean of Males	0.8314							
Mean of Females	0.8049							
Difference between means	0.02641							
SE of difference	0.02701							
95% CI of difference	-0.03244 to 0.08526							
Data summary								
Number of columns (Diet)	3							
Number of rows (Sex)	2							
Number of values	18							
Number of families	1							
Number of comparisons per family	15							
Alpha	0.05							
Tukey's multiple comparisons test	Mean Diff.	95.00% CI of diff.	Below thres	Summary	Adjusted P Value			
Males :Iron Adequate (IA) vs. Males :Iron Deficient (ID)	0.1744	0.01726 to 0.3315	Yes	*	0.0269			
Males :Iron Adequate (IA) vs. Males :Iron Repletion (IR)	0.1032	-0.05391 to 0.2604	No	ns	0.3021			
Males :Iron Adequate (IA) vs. Females:Iron Adequate (IA)	-0.01033	-0.1675 to 0.1468	No	ns	>0.9999			
Males :Iron Adequate (IA) vs. Females:Iron Deficient (ID)	0.178	0.02086 to 0.3351	Yes	*	0.0236			
Males :Iron Adequate (IA) vs. Females:Iron Repletion (IR)	0.1892	0.03206 to 0.3463	Yes	*	0.0157			
Males :Iron Deficient (ID) vs. Males :Iron Repletion (IR)	-0.07117	-0.2283 to 0.08597	No	ns	0.6587			
Males :Iron Deficient (ID) vs. Females:Iron Adequate (IA)	-0.1847	-0.3419 to -0.02759	Yes	*	0.0185			
Males :Iron Deficient (ID) vs. Females:Iron Deficient (ID)	0.0036	-0.1535 to 0.1607	No	ns	>0.9999			
Males :Iron Deficient (ID) vs. Females:Iron Repletion (IR)	0.0148	-0.1423 to 0.1719	No	ns	0.9995			
Males :Iron Repletion (IR) vs. Females:Iron Adequate (IA)	-0.1136	-0.2707 to 0.04357	No	ns	0.2209			
Males :Iron Repletion (IR) vs. Females:Iron Deficient (ID)	0.07477	-0.08237 to 0.2319	No	ns	0.6145			
Males :Iron Repletion (IR) vs. Females:Iron Repletion (IR)	0.08597	-0.07117 to 0.2431	No	ns	0.4796			
Females:Iron Adequate (IA) vs. Females:Iron Deficient (ID)	0.1883	0.03119 to 0.3455	Yes	*	0.0162			
Females:Iron Adequate (IA) vs. Females:Iron Repletion (IR)	0.1995	0.04239 to 0.3567	Yes	*	0.0109			
Females:Iron Deficient (ID) vs. Females:Iron Repletion (IR)	0.0112	-0.1459 to 0.1683	No	ns	0.9999			
Test details	Mean 1	Mean 2	Mean Diff.	SE of diff.	N1	N2	q	DF
Males :Iron Adequate (IA) vs. Males :Iron Deficient (ID)	0.9239	0.7495	0.1744	0.04678	3	3	5.272	12
Males :Iron Adequate (IA) vs. Males :Iron Repletion (IR)	0.9239	0.8207	0.1032	0.04678	3	3	3.121	12
Males :Iron Adequate (IA) vs. Females:Iron Adequate (IA)	0.9239	0.9342	-0.01033	0.04678	3	3	0.3124	12
Males :Iron Adequate (IA) vs. Females:Iron Deficient (ID)	0.9239	0.7459	0.178	0.04678	3	3	5.381	12
Males :Iron Adequate (IA) vs. Females:Iron Repletion (IR)	0.9239	0.7347	0.1892	0.04678	3	3	5.719	12
Males :Iron Deficient (ID) vs. Males :Iron Repletion (IR)	0.7495	0.8207	-0.07117	0.04678	3	3	2.151	12
Males :Iron Deficient (ID) vs. Females:Iron Adequate (IA)	0.7495	0.9342	-0.1847	0.04678	3	3	5.584	12
Males :Iron Deficient (ID) vs. Females:Iron Deficient (ID)	0.7495	0.7459	0.0036	0.04678	3	3	0.1088	12
Males :Iron Deficient (ID) vs. Females:Iron Repletion (IR)	0.7495	0.7347	0.0148	0.04678	3	3	0.4474	12
Males :Iron Repletion (IR) vs. Females:Iron Adequate (IA)	0.8207	0.9342	-0.1136	0.04678	3	3	3.433	12
Males :Iron Repletion (IR) vs. Females:Iron Deficient (ID)	0.8207	0.7459	0.07477	0.04678	3	3	2.26	12
Males :Iron Repletion (IR) vs. Females:Iron Repletion (IR)	0.8207	0.7347	0.08597	0.04678	3	3	2.599	12
Females:Iron Adequate (IA) vs. Females:Iron Deficient (ID)	0.9342	0.7459	0.1883	0.04678	3	3	5.693	12
Females:Iron Adequate (IA) vs. Females:Iron Repletion (IR)	0.9342	0.7347	0.1995	0.04678	3	3	6.032	12
Females:Iron Deficient (ID) vs. Females:Iron Repletion (IR)	0.7459	0.7347	0.0112	0.04678	3	3	0.3386	12

Figure 7E

Table Analyzed	**Figure 7E** SOD2 WB Veh x diet							
Two-way ANOVA	Ordinary							
Alpha	0.05							
Source of Variation	% of total variation	P value	P value sun	Significant?				
Interaction	3.316	0.4614	ns	No				
Sex	33.72	0.0015	**	Yes				
Diet	38.86	0.0031	**	Yes				
ANOVA table	SS	DF	MS	F (DFn, DFd)	P value			
Interaction	0.02132	2	0.01066	F (2, 12) = 0.8255	P=0.4614			
Sex	0.2167	1	0.2167	F (1, 12) = 16.79	P=0.0015			
Diet	0.2498	2	0.1249	F (2, 12) = 9.673	P=0.0031			
Residual	0.1549	12	0.01291					
Difference between row means								
Mean of Males	1.039							
Mean of Females	1.258							
Difference between means	-0.2195							
SE of difference	0.05356							
95% CI of difference	-0.3362 to -0.1028							
Data summary								
Number of columns (Diet)	3							
Number of rows (Sex)	2							
Number of values	18							
Number of families	1							
Number of comparisons per family	15							
Alpha	0.05							
Tukey's multiple comparisons test	Mean Diff.	95.00% CI of diff.	Below thres	Summary	Adjusted P Value			
Males :Iron Adequate (IA) vs. Males :Iron Deficient (ID)	0.03697	-0.2746 to 0.3486	No	ns	0.9984			
Males :Iron Adequate (IA) vs. Males :Iron Repletion (IR)	-0.2449	-0.5565 to 0.06668	No	ns	0.1604			
Males :Iron Adequate (IA) vs. Females:Iron Adequate (IA)	-0.3167	-0.6283 to -0.005050	Yes	*	0.0456			
Males :Iron Adequate (IA) vs. Females:Iron Deficient (ID)	-0.1295	-0.4411 to 0.1821	No	ns	0.729			
Males :Iron Adequate (IA) vs. Females:Iron Repletion (IR)	-0.4202	-0.7318 to -0.1086	Yes	**	0.007			
Males :Iron Deficient (ID) vs. Males :Iron Repletion (IR)	-0.2819	-0.5935 to 0.02972	No	ns	0.085			
Males :Iron Deficient (ID) vs. Females:Iron Adequate (IA)	-0.3536	-0.6652 to -0.04202	Yes	*	0.0233			
Males :Iron Deficient (ID) vs. Females:Iron Deficient (ID)	-0.1665	-0.4781 to 0.1451	No	ns	0.5032			
Males :Iron Deficient (ID) vs. Females:Iron Repletion (IR)	-0.4572	-0.7688 to -0.1456	Yes	**	0.0036			
Males :Iron Repletion (IR) vs. Females:Iron Adequate (IA)	-0.07173	-0.3833 to 0.2399	No	ns	0.9671			
Males :Iron Repletion (IR) vs. Females:Iron Deficient (ID)	0.1154	-0.1962 to 0.4270	No	ns	0.8078			
Males :Iron Repletion (IR) vs. Females:Iron Repletion (IR)	-0.1753	-0.4869 to 0.1363	No	ns	0.452			
Females:Iron Adequate (IA) vs. Females:Iron Deficient (ID)	0.1872	-0.1244 to 0.4988	No	ns	0.387			
Females:Iron Adequate (IA) vs. Females:Iron Repletion (IR)	-0.1035	-0.4151 to 0.2081	No	ns	0.8658			
Females:Iron Deficient (ID) vs. Females:Iron Repletion (IR)	-0.2907	-0.6023 to 0.02092	No	ns	0.0728			
Test details	Mean 1	Mean 2	Mean Diff.	SE of diff.	N1	N2	q	DF
Males :Iron Adequate (IA) vs. Males :Iron Deficient (ID)	0.9694	0.9324	0.03697	0.09277	3	3	0.5635	12
Males :Iron Adequate (IA) vs. Males :Iron Repletion (IR)	0.9694	1.214	-0.2449	0.09277	3	3	3.734	12
Males :Iron Adequate (IA) vs. Females:Iron Adequate (IA)	0.9694	1.286	-0.3167	0.09277	3	3	4.827	12
Males :Iron Adequate (IA) vs. Females:Iron Deficient (ID)	0.9694	1.099	-0.1295	0.09277	3	3	1.974	12
Males :Iron Adequate (IA) vs. Females:Iron Repletion (IR)	0.9694	1.39	-0.4202	0.09277	3	3	6.405	12
Males :Iron Deficient (ID) vs. Males :Iron Repletion (IR)	0.9324	1.214	-0.2819	0.09277	3	3	4.297	12
Males :Iron Deficient (ID) vs. Females:Iron Adequate (IA)	0.9324	1.286	-0.3536	0.09277	3	3	5.391	12
Males :Iron Deficient (ID) vs. Females:Iron Deficient (ID)	0.9324	1.099	-0.1665	0.09277	3	3	2.538	12
Males :Iron Deficient (ID) vs. Females:Iron Repletion (IR)	0.9324	1.39	-0.4572	0.09277	3	3	6.969	12
Males :Iron Repletion (IR) vs. Females:Iron Adequate (IA)	1.214	1.286	-0.07173	0.09277	3	3	1.093	12
Males :Iron Repletion (IR) vs. Females:Iron Deficient (ID)	1.214	1.099	0.1154	0.09277	3	3	1.76	12
Males :Iron Repletion (IR) vs. Females:Iron Repletion (IR)	1.214	1.39	-0.1753	0.09277	3	3	2.672	12
Females:Iron Adequate (IA) vs. Females:Iron Deficient (ID)	1.286	1.099	0.1872	0.09277	3	3	2.853	12
Females:Iron Adequate (IA) vs. Females:Iron Repletion (IR)	1.286	1.39	-0.1035	0.09277	3	3	1.578	12
Females:Iron Deficient (ID) vs. Females:Iron Repletion (IR)	1.099	1.39	-0.2907	0.09277	3	3	4.431	12

Figure 7F

Table Analyzed	**Figure 7F** SOD2 WB LD x diet							
Two-way ANOVA	Ordinary							
Alpha	0.05							
Source of Variation	% of total variation	P value	P value sun	Significant?				
Interaction	26.44	0.0313	*	Yes				
Sex	5.754	0.1786	ns	No				
Diet	33.98	0.0154	*	Yes				
ANOVA table	SS	DF	MS	F (DFn, DFd)	P value			
Interaction	0.2752	2	0.1376	F (2, 12) = 4.690	P=0.0313			
Sex	0.05989	1	0.05989	F (1, 12) = 2.041	P=0.1786			
Diet	0.3537	2	0.1768	F (2, 12) = 6.027	P=0.0154			
Residual	0.3521	12	0.02934					
Difference between row means								
Mean of Males	1.055							
Mean of Females	1.171							
Difference between means	-0.1154							
SE of difference	0.08075							
95% CI of difference	-0.2913 to 0.06057							
Data summary								
Number of columns (Diet)	3							
Number of rows (Sex)	2							
Number of values	18							
Number of families	1							
Number of comparisons per family	15							
Alpha	0.05							
Tukey's multiple comparisons test	Mean Diff.	95.00% CI of diff.	Below thres	Summary	Adjusted P Value			
Males :Iron Adequate (IA) vs. Males :Iron Deficient (ID)	-0.01883	-0.4886 to 0.4509	No	ns	>0.9999			
Males :Iron Adequate (IA) vs. Males :Iron Repletion (IR)	0.4714	0.001651 to 0.9412	Yes	*	0.049			
Males :Iron Adequate (IA) vs. Females:Iron Adequate (IA)	-0.145	-0.6148 to 0.3247	No	ns	0.8964			
Males :Iron Adequate (IA) vs. Females:Iron Deficient (ID)	0.1824	-0.2873 to 0.6522	No	ns	0.7776			
Males :Iron Adequate (IA) vs. Females:Iron Repletion (IR)	0.0691	-0.4007 to 0.5389	No	ns	0.9955			
Males :Iron Deficient (ID) vs. Males :Iron Repletion (IR)	0.4903	0.02048 to 0.9600	Yes	*	0.0391			
Males :Iron Deficient (ID) vs. Females:Iron Adequate (IA)	-0.1262	-0.5960 to 0.3436	No	ns	0.9386			
Males :Iron Deficient (ID) vs. Females:Iron Deficient (ID)	0.2013	-0.2685 to 0.6710	No	ns	0.7051			
Males :Iron Deficient (ID) vs. Females:Iron Repletion (IR)	0.08793	-0.3818 to 0.5577	No	ns	0.9865			
Males :Iron Repletion (IR) vs. Females:Iron Adequate (IA)	-0.6165	-1.086 to -0.1467	Yes	**	0.0086			
Males :Iron Repletion (IR) vs. Females:Iron Deficient (ID)	-0.289	-0.7588 to 0.1808	No	ns	0.3637			
Males :Iron Repletion (IR) vs. Females:Iron Repletion (IR)	-0.4023	-0.8721 to 0.06745	No	ns	0.1105			
Females:Iron Adequate (IA) vs. Females:Iron Deficient (ID)	0.3275	-0.1423 to 0.7972	No	ns	0.2503			
Females:Iron Adequate (IA) vs. Females:Iron Repletion (IR)	0.2141	-0.2556 to 0.6839	No	ns	0.653			
Females:Iron Deficient (ID) vs. Females:Iron Repletion (IR)	-0.1133	-0.5831 to 0.3564	No	ns	0.9601			
Test details	Mean 1	Mean 2	Mean Diff.	SE of diff.	N1	N2	q	DF
Males :Iron Adequate (IA) vs. Males :Iron Deficient (ID)	1.206	1.225	-0.01883	0.1399	3	3	0.1904	12
Males :Iron Adequate (IA) vs. Males :Iron Repletion (IR)	1.206	0.7346	0.4714	0.1399	3	3	4.767	12
Males :Iron Adequate (IA) vs. Females:Iron Adequate (IA)	1.206	1.351	-0.145	0.1399	3	3	1.467	12
Males :Iron Adequate (IA) vs. Females:Iron Deficient (ID)	1.206	1.024	0.1824	0.1399	3	3	1.845	12
Males :Iron Adequate (IA) vs. Females:Iron Repletion (IR)	1.206	1.137	0.0691	0.1399	3	3	0.6987	12
Males :Iron Deficient (ID) vs. Males :Iron Repletion (IR)	1.225	0.7346	0.4903	0.1399	3	3	4.957	12
Males :Iron Deficient (ID) vs. Females:Iron Adequate (IA)	1.225	1.351	-0.1262	0.1399	3	3	1.276	12
Males :Iron Deficient (ID) vs. Females:Iron Deficient (ID)	1.225	1.024	0.2013	0.1399	3	3	2.035	12
Males :Iron Deficient (ID) vs. Females:Iron Repletion (IR)	1.225	1.137	0.08793	0.1399	3	3	0.8891	12
Males :Iron Repletion (IR) vs. Females:Iron Adequate (IA)	0.7346	1.351	-0.6165	0.1399	3	3	6.233	12
Males :Iron Repletion (IR) vs. Females:Iron Deficient (ID)	0.7346	1.024	-0.289	0.1399	3	3	2.922	12
Males :Iron Repletion (IR) vs. Females:Iron Repletion (IR)	0.7346	1.137	-0.4023	0.1399	3	3	4.068	12
Females:Iron Adequate (IA) vs. Females:Iron Deficient (ID)	1.351	1.024	0.3275	0.1399	3	3	3.311	12
Females:Iron Adequate (IA) vs. Females:Iron Repletion (IR)	1.351	1.137	0.2141	0.1399	3	3	2.165	12
Females:Iron Deficient (ID) vs. Females:Iron Repletion (IR)	1.024	1.137	-0.1133	0.1399	3	3	1.146	12

Figure 7G

Table Analyzed	**Figure 7G** SOD2 WB Sel x diet							
Two-way ANOVA	Ordinary							
Alpha	0.05							
Source of Variation	% of total variation	P value	P value sun	Significant?				
Interaction	35.8	0.0007	***	Yes				
Sex	8.062	0.0262	*	Yes				
Diet	41.07	0.0004	***	Yes				
ANOVA table	SS	DF	MS	F (DFn, DFd)	P value			
Interaction	0.1603	2	0.08015	F (2, 12) = 14.25	P=0.0007			
Sex	0.0361	1	0.0361	F (1, 12) = 6.420	P=0.0262			
Diet	0.1839	2	0.09195	F (2, 12) = 16.35	P=0.0004			
Residual	0.06748	12	0.005623					
Difference between row means								
Mean of Males	1.109							
Mean of Females	1.02							
Difference between means	0.08957							
SE of difference	0.03535							
95% CI of difference	0.01254 to 0.1666							
Data summary								
Number of columns (Diet)	3							
Number of rows (Sex)	2							
Number of values	18							
Number of families	1							
Number of comparisons per family	15							
Alpha	0.05							
Tukey's multiple comparisons test	Mean Diff.	95.00% CI of diff.	Below thres	Summary	Adjusted P Value			
Males :Iron Adequate (IA) vs. Males :Iron Deficient (ID)	0.04113	-0.1645 to 0.2468	No	ns	0.9819			
Males :Iron Adequate (IA) vs. Males :Iron Repletion (IR)	-0.005	-0.2107 to 0.2007	No	ns	>0.9999			
Males :Iron Adequate (IA) vs. Females:Iron Adequate (IA)	-0.1722	-0.3779 to 0.03343	No	ns	0.1224			
Males :Iron Adequate (IA) vs. Females:Iron Deficient (ID)	0.2165	0.01087 to 0.4222	Yes	*	0.0371			
Males :Iron Adequate (IA) vs. Females:Iron Repletion (IR)	0.2605	0.05487 to 0.4662	Yes	*	0.011			
Males :Iron Deficient (ID) vs. Males :Iron Repletion (IR)	-0.04613	-0.2518 to 0.1595	No	ns	0.9705			
Males :Iron Deficient (ID) vs. Females:Iron Adequate (IA)	-0.2134	-0.4190 to -0.007704	Yes	*	0.0405			
Males :Iron Deficient (ID) vs. Females:Iron Deficient (ID)	0.1754	-0.03026 to 0.3811	No	ns	0.1127			
Males :Iron Deficient (ID) vs. Females:Iron Repletion (IR)	0.2194	0.01374 to 0.4251	Yes	*	0.0343			
Males :Iron Repletion (IR) vs. Females:Iron Adequate (IA)	-0.1672	-0.3729 to 0.03843	No	ns	0.1392			
Males :Iron Repletion (IR) vs. Females:Iron Deficient (ID)	0.2215	0.01587 to 0.4272	Yes	*	0.0323			
Males :Iron Repletion (IR) vs. Females:Iron Repletion (IR)	0.2655	0.05987 to 0.4712	Yes	**	0.0096			
Females:Iron Adequate (IA) vs. Females:Iron Deficient (ID)	0.3888	0.1831 to 0.5944	Yes	***	0.0004			
Females:Iron Adequate (IA) vs. Females:Iron Repletion (IR)	0.4328	0.2271 to 0.6384	Yes	***	0.0001			
Females:Iron Deficient (ID) vs. Females:Iron Repletion (IR)	0.044	-0.1617 to 0.2497	No	ns	0.9758			
Test details	Mean 1	Mean 2	Mean Diff.	SE of diff.	N1	N2	q	DF
Males :Iron Adequate (IA) vs. Males :Iron Deficient (ID)	1.122	1.08	0.04113	0.06123	3	3	0.9501	12
Males :Iron Adequate (IA) vs. Males :Iron Repletion (IR)	1.122	1.127	-0.005	0.06123	3	3	0.1155	12
Males :Iron Adequate (IA) vs. Females:Iron Adequate (IA)	1.122	1.294	-0.1722	0.06123	3	3	3.978	12
Males :Iron Adequate (IA) vs. Females:Iron Deficient (ID)	1.122	0.905	0.2165	0.06123	3	3	5.001	12
Males :Iron Adequate (IA) vs. Females:Iron Repletion (IR)	1.122	0.861	0.2605	0.06123	3	3	6.018	12
Males :Iron Deficient (ID) vs. Males :Iron Repletion (IR)	1.08	1.127	-0.04613	0.06123	3	3	1.066	12
Males :Iron Deficient (ID) vs. Females:Iron Adequate (IA)	1.08	1.294	-0.2134	0.06123	3	3	4.928	12
Males :Iron Deficient (ID) vs. Females:Iron Deficient (ID)	1.08	0.905	0.1754	0.06123	3	3	4.051	12
Males :Iron Deficient (ID) vs. Females:Iron Repletion (IR)	1.08	0.861	0.2194	0.06123	3	3	5.068	12
Males :Iron Repletion (IR) vs. Females:Iron Adequate (IA)	1.127	1.294	-0.1672	0.06123	3	3	3.863	12
Males :Iron Repletion (IR) vs. Females:Iron Deficient (ID)	1.127	0.905	0.2215	0.06123	3	3	5.117	12
Males :Iron Repletion (IR) vs. Females:Iron Repletion (IR)	1.127	0.861	0.2655	0.06123	3	3	6.133	12
Females:Iron Adequate (IA) vs. Females:Iron Deficient (ID)	1.294	0.905	0.3888	0.06123	3	3	8.979	12
Females:Iron Adequate (IA) vs. Females:Iron Repletion (IR)	1.294	0.861	0.4328	0.06123	3	3	9.996	12
Females:Iron Deficient (ID) vs. Females:Iron Repletion (IR)	0.905	0.861	0.044	0.06123	3	3	1.016	12
